# NHC‐Terphenyl Radicals and Anions: Tuning Stability and Redox Properties via Substituent Patterning

**DOI:** 10.1002/anie.202520260

**Published:** 2025-10-22

**Authors:** Henric Steffenfauseweh, Yury V. Vishnevskiy, Beate Neumann, Hans‐Georg Stammler, Bas de Bruin, Rajendra S. Ghadwal

**Affiliations:** ^1^ Molecular Inorganic Chemistry and Catalysis Inorganic and Structural Chemistry Center for Molecular Materials Faculty of Chemistry Universität Bielefeld Universitätsstrasse 25 D‐33615 Bielefeld Germany; ^2^ University of Amsterdam (UvA) Faculty of Science Van 't Hoff Institute for Molecular Sciences (HIMS) Homogeneous and Supramolecular Catalysis Group Science Park 904 Amsterdam 1098 XH The Netherlands

**Keywords:** C–H Activation, *N*‐heterocyclic carbene, Stable radicals and anions, Structures

## Abstract

Herein, we report the influence of C2‐terphenyl substitution patterns (i.e., *p*‐terphenyl versus *m*‐terphenyl) on the redox behavior and stability of the corresponding radicals and anions derived from *N*‐heterocyclic carbenes (NHCs). Three well‐known NHCs; SIPr (**1a**), IPr (**1b**), and Me‐IPr (**1c**) (SIPr = C{N(Dipp)CH_2_}_2_, IPr = C{N(Dipp)CH}_2_; Me‐IPr = C{N(Dipp)CCH_3_}_2_; Dipp = 2,6‐*i*Pr_2_C_6_H_3_); were functionalized at the C2 position using 4‐bromo‐*p*‐terphenyl (*p*‐TerBr) and 5′‐bromo‐*m*‐terphenyl (*m*‐TerBr) under nickel catalysis, yielding the corresponding cations [(NHC)*p*‐Ter]Br (**2a**–**c**) and [(NHC)*m*‐Ter]Br (**3a**–**c**), respectively. Cyclic voltammetry (CV) measurements of **2a**–**c** reveal two distinct reversible redox events, while **3a**–**c** exhibit one reversible and one irreversible or quasi‐reversible wave. Reduction of **2a**–**c** and **3a**–**c** with KC_8_ readily affords stable radicals [(NHC)*p*‐Ter]^●^ (**4a**–**c**) and [(NHC‐*m*‐Ter]^●^ (**5a**–**c**), isolated as crystalline solids. Further reduction of **4a**–**c** produces diamagnetic anions [(NHC)*p*‐Ter]K (**6a**–**c‐K**), consistent with the electrochemical data. In contrast, **5b** and **5c** are unreactive toward KC_8_ under similar conditions, while **5a** (derived from the more electrophilic NHC **1a**) can be reduced to the corresponding anion [(SIPr)*m*‐Ter]K (**7a‐K**). Selected compounds have been characterized by spectroscopic techniques and single‐crystal X‐ray diffraction, with computational studies supporting the experimental findings. The results highlight how the NHC and the C2‐terphenyl substituent influence the properties and stability of the resulting species.

## Graphical abstract

Two series of C2‐terphenylated derivatives of three *N*‐heterocyclic carbenes (NHCs) have been reported. The *para*‐terphenylated species exhibit three stable (reversible) redox states (e.g., [**2b**]^+^, [**2b**]^●^ and [**2b**]^−^), while for the *meta*‐terphenylated derivatives only cationic and neutral states (i.e., [**3b**]^+^ and [**3b**]^●^) are reversible.

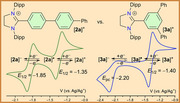

## Introduction

Organic compounds capable of maneuver between multiple redox states are of growing importance in modern materials science, with promising applications in data^[^ ^1^, ^2^, ^3^ ^]^ and energy storage,^[^ ^4^, ^5^, ^6^, ^7^, ^8^, ^9^, ^10^ ^]^ quantum information technologies,^[^ ^11^, ^12^ ^]^ and biomedical fields.^[^ ^13^, ^14^ ^]^ Among these, stable organic radicals serve as key molecular building blocks for optoelectronic^[^ ^15^, ^16^, ^17^, ^18^, ^19^, ^20^, ^21^, ^22^, ^23^, ^24^, ^25^, ^26^, ^27^ ^]^ and energy‐related materials,^[^ ^28^, ^29^ ^]^ owing to their open‐shell electronic structures, which impart valuable electronic, magnetic, and optical properties.^[^ ^30^, ^31^, ^32^ ^]^ Traditionally, radicals in organic chemistry have been viewed as highly reactive intermediates.^[^ ^33^ ^]^ The first stable organic radical, the so‐called trityl radical Ph_3_C^●^, was isolated by Gomberg in 1900.^[^ ^34^ ^]^ Since then, numerous radicals have been synthesized—most featuring halogenated substituents on the carbon center or redox‐active heteroatoms such as nitrogen, oxygen, or sulfur.^[^ ^35^, ^36^, ^37^ ^]^ However, truly carbon‐centered stable radicals remained rare.^[^ ^38^, ^39^, ^40^ ^]^ Recent studies nonetheless emphasized that achieving stability in organic radicals requires carefully designed molecules that provide both steric protection and/or spin delocalization.^[^ ^38^, ^39^, ^40^ ^]^ This insight led to the development of thermally stable carbon‐centered radicals based on polycyclic aromatic hydrocarbons^[^ ^40^, ^41^, ^42^, ^43^, ^44^ ^]^ and *N*‐heterocyclic carbenes (NHCs),^[^ ^45^, ^46^, ^47^, ^48^, ^49^, ^50^ ^]^ many of which were successfully isolated in the late 2010s. Among stable singlet carbenes,^[^ ^51^, ^52^, ^53^, ^54^ ^]^ the use of cyclic alkyl amino carbenes (cAACs)^[^ ^55^, ^56^, ^57^, ^58^ ^]^ with remarkable electrophilic characteristics in the stabilization of radicals and related open‐shell species is noteworthy.^[^ ^55^, ^56^, ^57^, ^58^ ^]^

**Figure 1:**
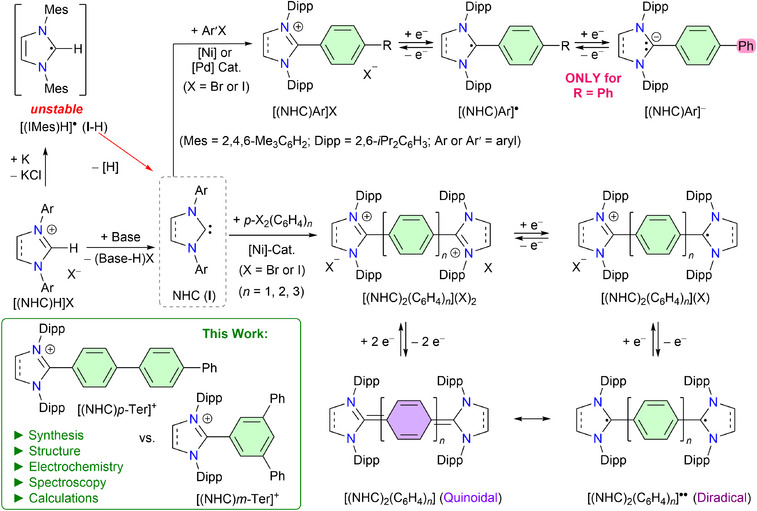
Schematic illustration of [(NHC)H]X, [(IMes)H]^●^, and NHC (**I**). NHC‐derived stable radicals [(NHC)Ar]^●^, anions [(NHC)Ar]^−^, radical cations [(NHC)_2_(C_6_H_4_)*_n_*]^●+^, and diradical(oid)s [(NHC)_2_(C_6_H_4_)*_n_*]^●●^.

In 2004, Clyburne et al., attempted synthesis of the radical **I**‐H (Figure 1) by reducing the corresponding 1,3‐imidazolium salt [(IMes)H]Cl with potassium in boiling THF.^[^ ^59^, ^60^ ^]^ **I**‐H was found to be unstable and decomposed to form the free NHC (IMes). This outcome was in agreement with the cyclic voltammogram (CV) of [(IMes)H]Cl, which exhibits an irreversible reduction at a high negative potential (*E*_pc_ = −2.38 V versus SCE, *E*_pc_ = cathodic peak potential).^[^ ^59^ ^]^ In contrast, the CVs of the related C2‐arylated 1,3‐imidazoli(ni)um salts [(NHC)Ar]X, which can be obtained by the direct C2‐arylation of NHCs (**I**) under Ni or Pd catalysis,^[^ ^61^, ^62^ ^]^ show one reversible redox process at a lower negative potential (> −2 V versus Ag/Ag^+^) related to the [(NHC)Ar]^+^/[(NHC)Ar]^●^ redox couple.^[^ ^63^ ^]^ Consistently, the radicals [(NHC)Ar]^●^ are isolable as stable crystalline solids. Moreover, the reduction potential (*E*_1/2_) of [(NHC)Ar]^+^ can be readily adjusted by varying the NHC and/or the C2‐aryl group. Also, the applications of such NHC‐based radicals in optoelectronic materials have been started to evolve.^[^ ^64^, ^65^, ^66^, ^67^, ^68^ ^]^

Expectedly, related dicationic compounds [(NHC)_2_(C_6_H_4_)*_n_*](X)_2_ with a phenylene (*n* = 1),^[^ ^69^, ^70^ ^]^ biphenylene (*n* = 2),^[^ ^69^, ^70^, ^71^, ^72^ ^]^ or terphenylene (*n* = 3)^[^ ^73^ ^]^ spacer show additional stable redox states and can be reduced to stable radical cations [(NHC)_2_(C_6_H_4_)*_n_*]^●+^ and diradical(oid)s [(NHC)_2_(C_6_H_4_)*_n_*]. The latter (see the diradical form) can also be considered as NHC‐derivatives of *para*‐quinodimethane (*p*‐QDM) (see the quinoidal form).^[^ ^74^ ^]^ The stability of these radical and diradical species is largely attributed to the delocalization of the unpaired electron over the C2‐aryl or the π‐spacer moiety (C_6_H_4_)*_n_*. Remarkably, a further extension of the C2‐aryl group at the *para* position by an aryl substituent such as biphenyl (Bp) in [(NHC)Bp]X results in the introduction of a new redox state as evident by their CVs, which show two reversible redox processes. The first corresponds to the [(NHC)Bp]^+^/[(NHC)Bp]^●^ and the second to the [(NHC)Bp]^●^/[(NHC)Bp]^−^. In fact, both radicals [(NHC)Bp]^●^ and anions [(NHC)Bp]^−^ are isolable stable compounds.^[^ ^75^ ^]^ Thus, the larger size of the C2‐substituent (i.e., Bp) provides more room for the delocalization of the unpaired electron and can also accommodate an extra electron, giving rise to anions. We therefore prompted to introduce an additional phenyl group to the C2‐aryl substituent in the [(NHC)Ar] framework and explore the structure and properties of related NHC‐derivatives.

Herein, we present synthesis of two classes of terphenylated systems namely [(NHC)*p*‐Ter]Br (**2a**, **2b**, **2c**) and [(NHC)*m*‐Ter]Br (**3a**, **3b**, **3c**) with three distinct NHCs namely SIPr (**1a**), IPr (**1b**), and Me‐IPr (**1c**) (SIPr = C{N(Dipp)CH_2_}_2_, IPr = C{N(Dipp)CH}_2_; Me‐IPr = C{N(Dipp)CCH_3_}_2_; Dipp = 2,6‐*i*Pr_2_C_6_H_3_) and report their structures, electrochemistry, and the corresponding radicals and anions.

## Results and Discussions

**Scheme 1:**
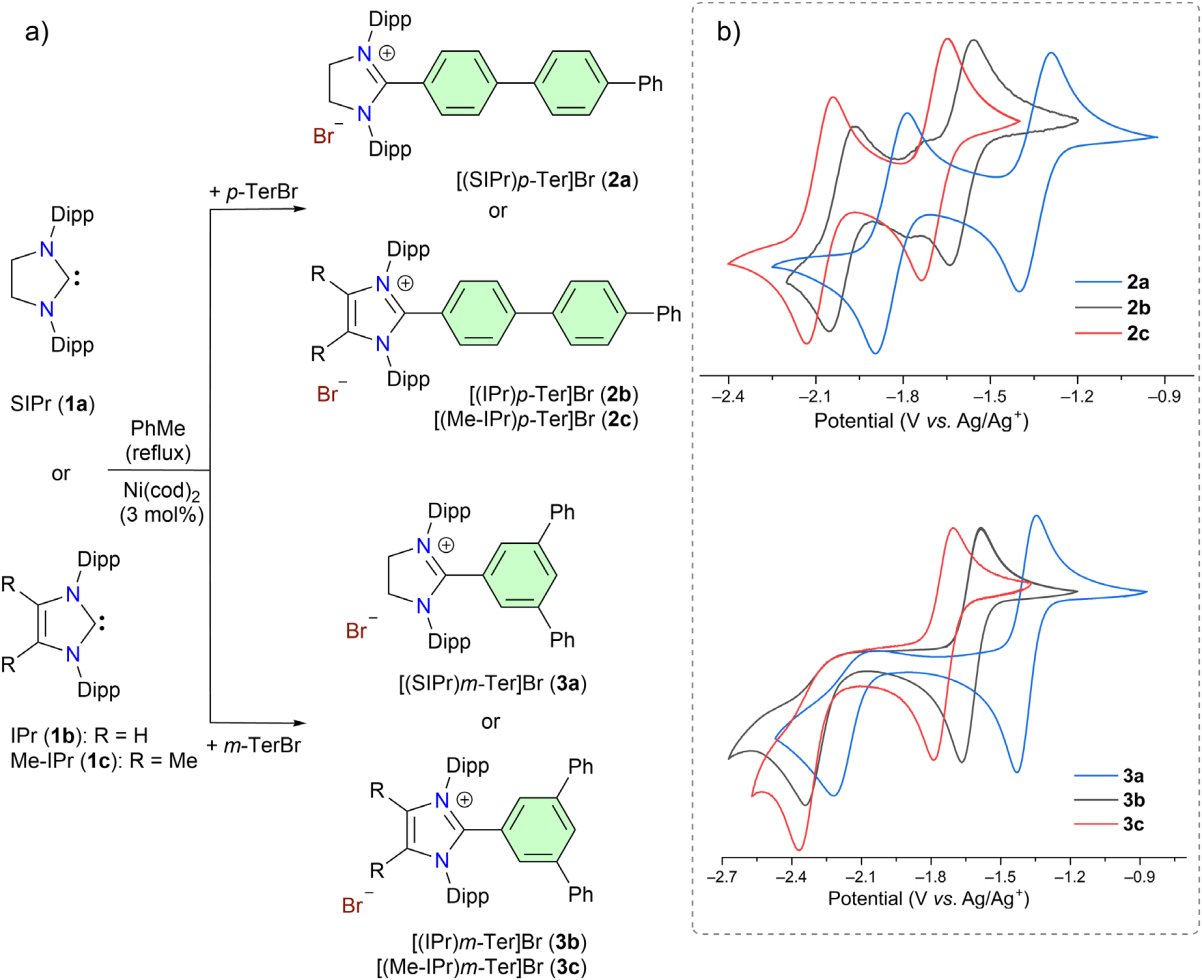
a) Synthesis of C2‐*p*‐terphenylated (**2a, 2b**, and **2c**) and C2‐*m*‐terphenylated (**3a, 3b**, and **3c**) 1,3‐imidazoli(ni)um salts from classical NHCs **1a, 1b**, and **1c**. b) Cyclic voltammograms (CVs) of **2a**, **2b**, **2c** and **3a**, **3b**, **3c** (in CH_3_CN, 0.1 M *n*Bu_4_NPF_6_, 100 mV s^−1^, versus Ag/Ag^+^).

**Figure 2:**
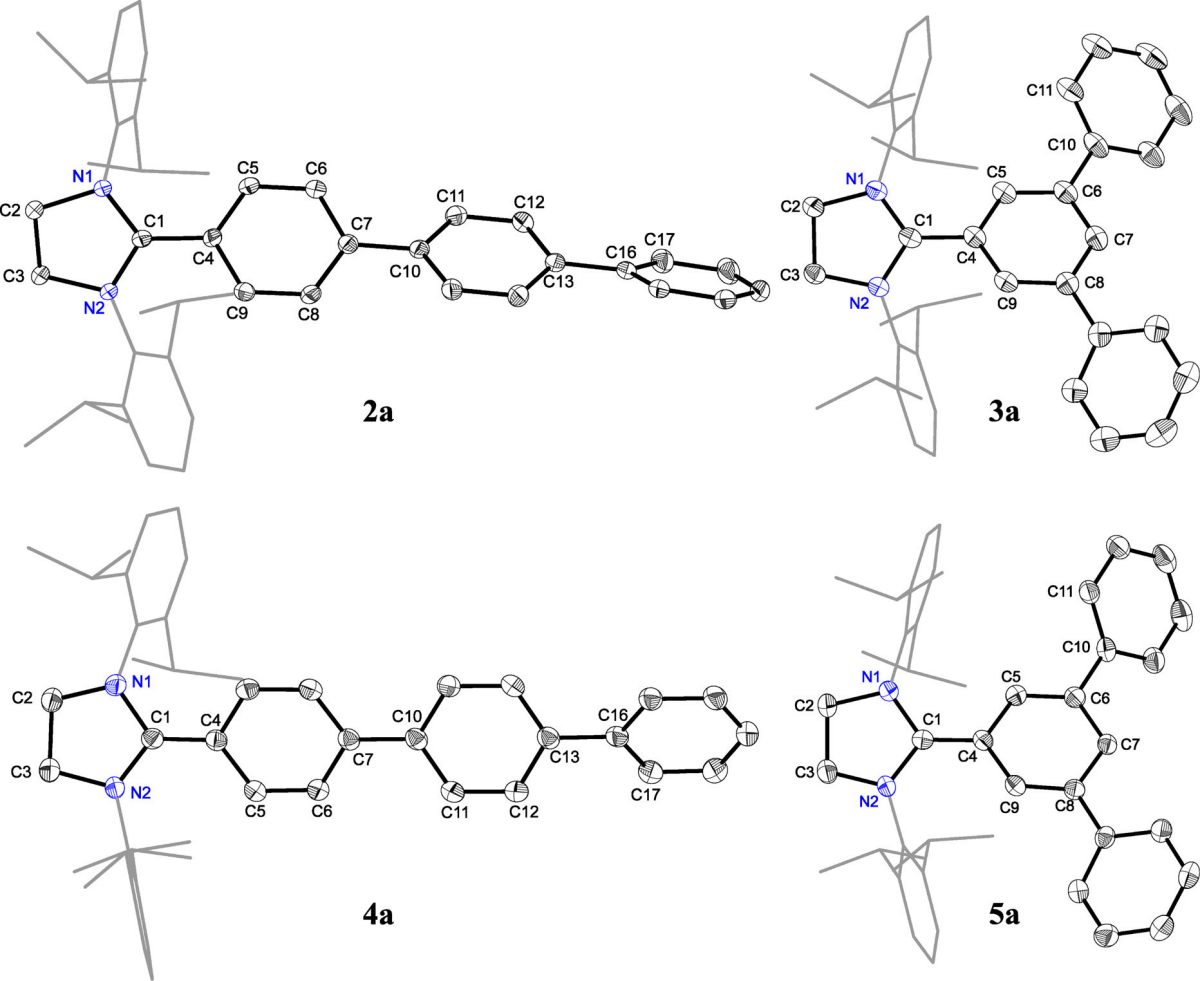
Solid‐state molecular structures of **2a**, **3a**, **4a**, and **5a**. Hydrogen atoms (and bromide anions for **2a** and **3a**) are omitted and Dipp groups are shown as wireframes for clarity. Thermal ellipsoids are set at 50% probability.

The desired 1,3‐imidazoli(ni)um bromides [(SIPr)*p*‐Ter]Br (**2a**), [(IPr)*p*‐Ter]Br (**2b**), [(Me‐IPr)*p‐*Ter]Br (**2c**), [(SIPr)*m*‐Ter]Br (**3a**), [(IPr)*m*‐Ter]Br (**3b**), and [(Me‐IPr)*m*‐Ter]Br (**3c**) (Scheme 1a) were prepared by the direct C2‐arylation of NHCs (i.e., SIPr (**1a**) = C{N(Dipp)CH_2_}_2_, IPr (**1b**) = C{N(Dipp)CH}_2_, Me‐IPr (**1c**) = C{N(Dipp)CCH_3_}_2_; Dipp = 2,6‐*i*Pr_2_C_6_H_3_) with an appropriate aryl bromide (4‐Bromo‐*p*‐terphenyl = *p*‐TerBr or 5′‐Bromo‐*m*‐terphenyl = *m*‐TerBr) using 3 mol% of Ni(cod)_2_ (cod = cyclooctadiene).^[^ ^61^ ^]^ Compounds **2a**–**c** and **3a**–**c** are colorless air‐stable crystalline solids. The ^1^H and ^13^C{^1^H} NMR spectra of **2a**–**c** and **3a**–**c** exhibit expected signals (see, Figures S5–S22), which are in line with those of known C2‐arylated 1,3‐imidazol(ni)um salts.^[^ ^61^, ^62^, ^63^, ^75^ ^]^ Single crystal X‐ray diffraction (sc‐XRD) analyses of **2a** and **3a** (Figure 2) show the expected atom connectivity (see below).^[^ ^76^ ^]^

The CVs of **2a**–**c** (Scheme 1b) show two reversible redox processes, which are similar to those of [(NHC)Bp]Br.^[^ ^75^ ^]^ The first redox event at *E*_1/2 _= −1.35 (for **2a**), −1.60 (for **2b**), and −1.70 V (for **2c**) (versus Ag/Ag^+^) is in line with the electrophilic characteristics of NHCs (**1a** > **1b** ≥ **1c**). A similar trend for the second reversible redox wave is also observed for **2a**–**c** [*E*_1/2_ = −1.85 (for **2a**), −2.00 (for **2b**), −2.10 V (for **2c**)]. The *E*_1/2_ values for the first and second redox events for **2a**–**c** are comparable to those of [(NHC)Bp]Br (NHC = SIPr: −1.31, −1.92 V; IPr: −1.51, −2.02 V, Me‐IPr: −1.59, −2.09 V).^[^ ^75^ ^]^ Like **2a**–**c**, the CVs of **3a**, **3b**, and **3c** (Scheme 1b) also show one reversible redox event at *E*_1/2_ = −1.40, −1.60, and −1.75 V (versus Ag/Ag^+^), respectively. However, the second reduction event at *E*_pc_ = −2.20, −2.30, and −2.35 V (*E*_pc_ = cathodic peak potential) for **3a**, **3b**, and **3c**, respectively, is irreversible. The outcomes of CVs studies suggest that one‐electron reduction of **2a**–**c** and **3a**–**c** to the corresponding neutral radicals should be feasible for all, but their further reductions to the corresponding anions seem more likely for the *p*‐terphenyl derivatives, i.e., [(NHC)*p*‐Ter]^−^.

**Scheme 2:**
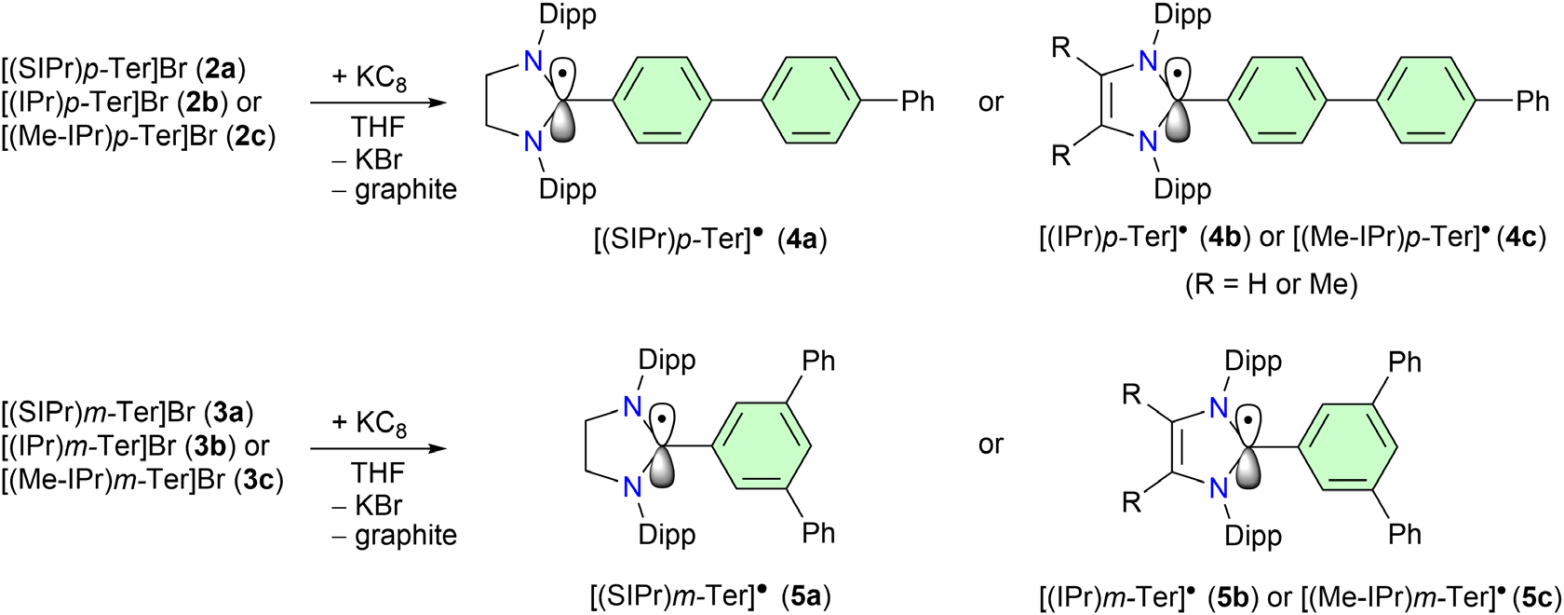
KC_8_ reductions of **2a**–**c** and **3a**–**c** to the radicals **4a**–**c** and **5a**–**c**, respectively.

Treatments of **2a**, **2b**, and **2c** with one equivalent of potassium graphite (KC_8_) yield the radicals, [(SIPr)*p*‐Ter]^●^ (**4a**) (green), [(IPr)*p*‐Ter]^●^ (**4b**) (green), and [(Me‐IPr)*p*‐Ter]^●^ (**4c**) (yellow‐green) as crystalline solids, respectively (Scheme 2). Similarly, reactions of **3a**, **3b**, and **3c** with one equivalent of KC_8_ afford crystalline radicals [(SIPr)*m*‐Ter]^●^ (**5a**) (green), [(IPr)*m*‐Ter]^●^ (**5b**) (green), and [(Me‐IPr)*m‐*Ter]^●^ (**5c**) (yellow‐green), respectively. Compounds **4a**–**c** and **5a**–**c** are stable at room temperature under an inert gas atmosphere and are NMR silent. The radicals **4a**–**c** and **5a**–**c** have been characterized by UV–vis and EPR spectroscopy as well as by sc‐XRD (Figure 2).

**Scheme 3:**
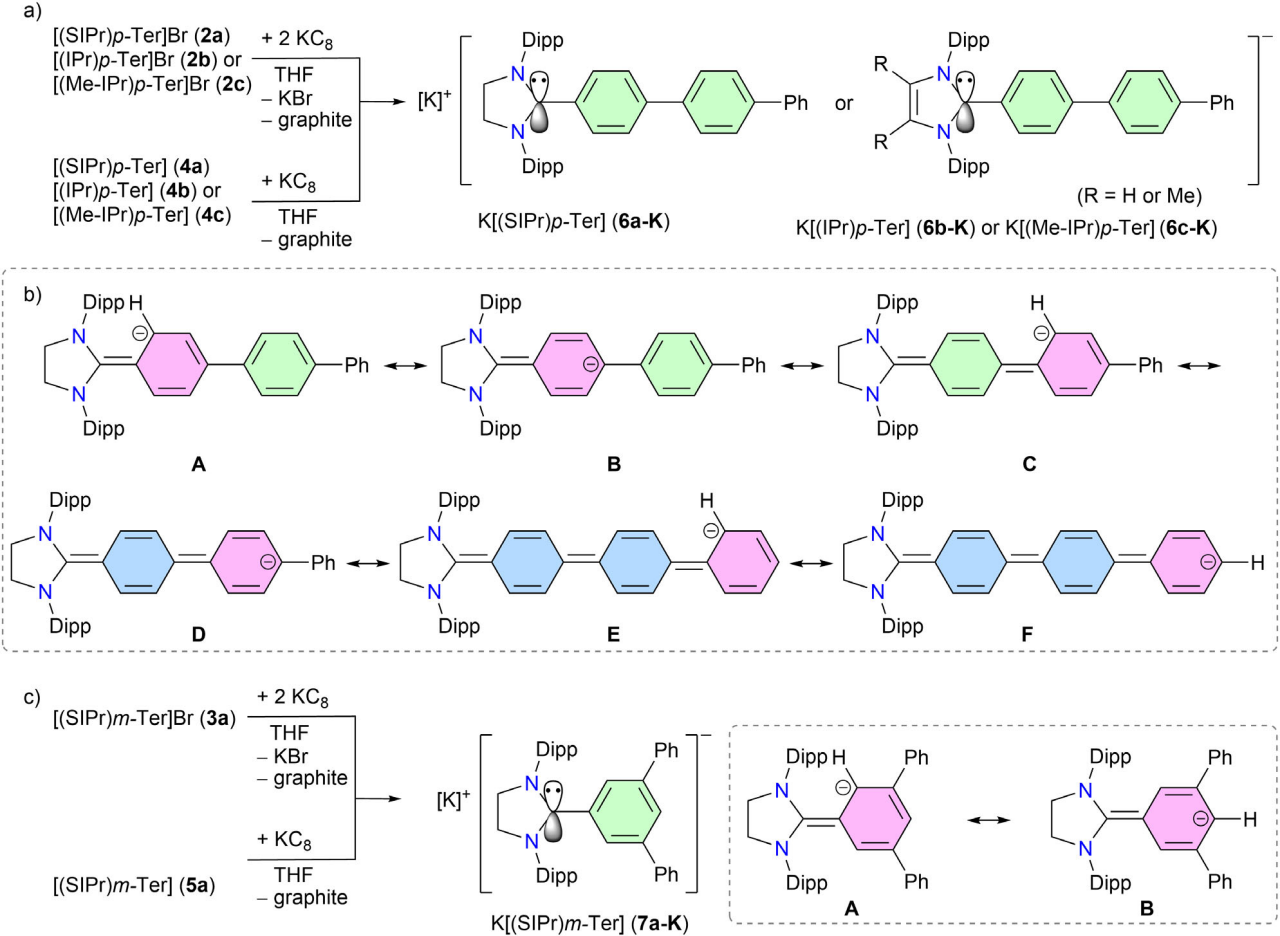
a) KC_8_ reductions of **2a**–**c** or **4a**–**c** to the anions **6a**–**c‐K**, respectively. b) Selected resonance forms (**A**–**F**) of the anion [**6a**]^−^. c) KC_8_ reductions of **3a** or **5a** to the anion **7a‐K** (with selected resonance forms **A** and **B**).

According to the CVs of **2a**–**c** (Scheme 1b), further 1e‐reductions of **4a**–**c** or direct two‐electron reductions of **2a**–**c** to the corresponding anions seem viable. Indeed, treatments of **2a**–**c** (or **4a**–**c**) with two equivalents (or one equivalent) of KC_8_ yields the anions K[(NHC)*p*‐Ter] (**6a**–**c‐K**) (Scheme 3a). Compounds **6a**–**c‐K** are extremely reactive and slowly decay in THF to partially form the corresponding radicals, making their characterization by NMR spectroscopy difficult. This is not surprising considering their strong tendency to be oxidized [cf. *E*_1/2_ = −1.85 (for **2a**), −2.00 (for **2b**), −2.10 V (for **2c**)]. This problem can be overcome by adding a small pinch of KC_8_ (∼ 2 mg for 0.5 mL THF‐*d*_8_ solution) into a NMR sample of **6a**–**c–K**. Compounds **6a**–**c–K** are diamagnetic and show well‐resolved NMR signals (Figures S23–S28). The ^1^H NMR spectra of **6a‐K** and **6c‐K** show expected signals for the NHC and *p*‐terphenyl moieties, which are high‐field shifted relative to those of **2a** and **2c**. In particular, the high‐field shifting of ^1^H NMR signals for the *p*‐terphenyl is more distinct and relates to NHC‐based *p*‐QDM derivatives.^[^ ^69^ ^]^ Compound **6a‐K** is rather stable and can be isolated as a blue crystalline solid. The stability of **6a**–**c‐K,** like radicals **4a**–**c** as well as previously reported anions K[(NHC)Bp],^[^ ^75^ ^]^ can be attributed to the delocalization of the electron lone pair over the C2‐*p*‐Ter group (see selected resonance structures **A**–**F**, Scheme 3b). DFT calculations further support this analysis (see below).

The CVs of **3a**, **3b**, and **3c** (Scheme 1b) feature a second irreversible reduction at a rather higher negative potential, *E*_pc_ = −2.20, −2.30, and − 2.35 V, respectively, relative to that of **2a**–**c**. Consistently, no reaction between a THF‐*d*_8_ solution **5b** or **5c** with KC_8_ was observed at room temperature as evident by no change in green color as well as in the ^1^H NMR spectrum. However, a green THF‐*d*_8_ solution of **5a** immediately turned deep blue on addition of KC_8_, suggesting the formation of the anion K[(SIPr)*m*‐Ter] (**7a‐K**). The ^1^H and ^13^C{^1^H} NMR spectra measured for the same sample reveal diamagnetic feature of **7a**‐**K**, which compare well with those of **6a‐K** and **6c‐K**. It is worth noting that the ^1^H NMR signals for the C_6_H_5_ protons of the *m*‐terphenyl group of **7a‐K** (6.92–6.96 ppm) appear in the aromatic region and are nearly comparable to those of **3a** (6.91–7.34 ppm in CDCl_3_). This indicates that the electron lone pair in **7a‐K** delocalizes only over the central C_6_H_3_‐ring (cf. resonance structure **A** and **B** in Scheme 3c) and both *m*‐C_6_H_5_ rings remain unchanged. This is further corroborated by DFT calculations (see below). The lower reduction potentials (**2a**, **3a**) and greater stability of the SIPr‐derivatives (**4a**, **5a** and **6a‐K**, **7a‐K**) than those of IPr‐ and Me‐IPr‐compounds (respectively, **2b**,**c**/**3b**,**c**; **4b**,**c/5b**,**c** and **6b**,**c‐K**, **7b**,**c‐K**) may be rationalized considering superior π‐acceptor property of SIPr with respect to that of IPr and Me‐IPr.^[^ ^77^, ^78^, ^79^ ^]^ Thus, the extent of π‐delocalization and hence the properties of derived molecules can be controlled by varying the nature of NHCs.^[^ ^80^ ^]^ Also, the formation of **4b**,**c** and **5b,c** (featuring 7π electron C_3_N_2_‐ring) from **2b**,**c** and **3b**,**c** (containing 6π electron planar C_3_N_2_‐ring), respectively, occurs at the expense of Hückel's aromaticity. This is not the case with SIPr‐derived compounds **4a**, **5a**, **6a‐K**, and **7a‐K** as they all have a non‐planar C_3_N_2_‐ring (i.e., non‐aromatic).

Suitable single crystals of **4a**–**c** and **5a** for sc‐XRD were obtained by storing a saturated *n*‐hexane solution of each at room temperature. The solid‐state molecular structures of **4a** and **5a** as well as their precursors **2a** and **3a** are shown in Figure 2 (see the Supporting Information for other species, Figures S58–S68). A comparative overview of the selected bond lengths and bond angles for **2a**/**4a** and **3a**/**5a** is given in Table 1. Like previously reported radicals [(NHC)Ar],^[^ ^63^, ^75^ ^]^ the C1–C4 (**4a**: 1.406(1); **5a**: 1.397(2) Å), and C1–N1/N2 (**4a**: 1.402(1)/1.393(1); **5a**: 1.399(2)/1.382(2) Å) bond lengths are respectively smaller and longer, while the N1─C1─N2 (**4a**: 108.2(1); **5a**: 108.6(1) °) bond angles are smaller for **4a** or **5a** with respect to those of **2a** or **3a** (Table 1). Moreover, the bond length alteration (BLA) for the C─C bonds of the terphenyl rings in **4a** and **5a** (0.05–0.08 Å) is larger than that of **2a** and **3a** (0.01 Å), respectively.

**Table 1:** Selected sc‐XRD [and calculated at PBEh‐3c] bond lengths and angles for **2a**, **3a**, **4a**, and **5a**.

Bond length [Å]/ bond angle or torsion angle [°]	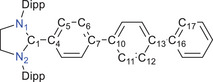		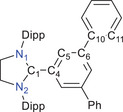	
	**2a**	**4a**	**3a**	**5a**
C1 − N1	1.334(2) [1.326]	1.402(1) [1.387]	1.324(2) [1.324]	1.399(2) [1.389]
C1–C4	1.475(2) [1.456]	1.406(1) [1.401]	1.471(3) [1.458]	1.397(2) [1.404]
C4–C5	1.396(2) [1.395]	1.430(1) [1.424]	1.397(2) [1.389]	1.434(2) [1.417]
C5–C6	1.389(2) [1.379]	1.376(1) [1.371]	1.396(3) [1.389]	1.368(2) [1.382]
C7–C10	1.485(2) [1.468]	1.467(2) [1.462]	–	–
C13–C16	1.490(2) [1.473]	1.479(1) [1.473]	–	–
C6–C10	–	–	1.489(3) [1.474]	1.484(2) [1.476]
N1–C1–N2	111.1(1) [111.3]	108.2(1) [107.7]	111.8(2) [111.6]	108.6(1) [108.1]
N1–C1–C4–C5	37.1(3) [38.7]	14.6(1) [18.4]	37.2(3) [39.5]	18.4(2) [19.6]
C6–C7–C10–C11^a)^	26.4(2)	8.4(1)	–	–
C12–C13–C16–C17^a)^	26.8(2)	31.1(1)	–	–

^a)^ The values correspond to small (positive) angles (according to its 180° inversion).

Similar to the C1─C4 bond lengths, the C7–C10 (**2a**: 1.485(2); **4a**: 1.467(2) Å) and C13–C16 (**2a**: 1.490(2); **4a**: 1.479(2) Å) bond lengths are shorter in **4a** than in **2a**. The bond length reduction is most pronounced at C1–C4 and becomes less significant across C7–C10 and C13–C16. In contrast, the C6–C10 bond lengths in **3a** and **5a** (**3a**: 1.489(3); **5a**: 1.484(2) Å) remain essentially unchanged. These observations suggest that in **4a** the electrons are well delocalized across all three rings, whereas in **5a** they are localized within a single ring. Consistent with the delocalization of the unpaired electron over the C2‐terphenyl ring, the N1–C1–C4–C5 torsion angle for **4a** (14.6(1)°) and **5a** (18.4(2)°) is smaller than that in **2a** (37.1(3)°) and **3a** (37.2(3)°), respectively. Thus, the C2‐aryl ring in **4a**/**5a** becomes more planar and less twisted relative to that in **2a**/**3a**. The central ring of the *p*‐Ter moiety of **4a** also adopts somewhat planarity (cf. C6–C7–C10–C11: 8.4(1)°) relative to that of **2a** (26.4(2)°), this is however less pronounced for the terminal C_6_H_5_ ring (cf. C12–C13–C16–C17 = 26.8(2)° for **2a** and 31.1(1)° for **4a**). These features indicate the delocalization of the unpaired electron over the respective C2‐terphenyl unit, which accounts for the remarkable thermal stability of the radicals **4a**–**c** and **5a**–**c**.

Remarkably, the C5–C6–C10–C11 torsion angle of **5a** (39.0(2)°) is larger than that of **3a** (27.1(3)°), excluding the delocalization of the unpaired electron to the *m*‐C_6_H_5_ rings. Similar trends in the structural parameters are observed for **4b** and **4c**. (Figures S63, S64). Attempts to obtain suitable single crystals of **6a‐K** and **7a‐K** were unfortunately unfruitful. The NMR data of **6a‐K** and **7a‐K** are fully consistent with a related anion characterized by sc‐XRD.^[^ ^75^ ^]^ The molecular structures of the anionic fragments [**6a**]^−^ and [**7a**]^−^ (i.e., excluding counter cation K^+^) have been analyzed by quantum chemical calculations (see below).

**Figure 3:**
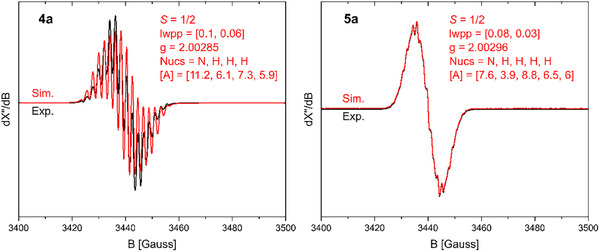
Experimental and simulated isotropic X‐band EPR spectra of [(SIPr)*p*‐Ter]^●^ (**4a**) (HFC to 2 equiv. N atoms, a set of 4 equiv. H atoms, a set of 2 equiv. H atoms and a single H atom) and [(SIPr)*m*‐Ter]^●^ (**5a**) (in toluene, at 298 K, 9.63 GHz, 0.3162 mW).

The optimized molecular structures of the selected radicals **4a** (Figure S71) and **5a** (Figure S72) at the PBEh‐3c level of theory are in good agreement with their solid‐state molecular structures (Figure 2) determined by sc‐XRD. The molecular structures of the anions [**6a**]^−^ (Figure S73) and [**7a**]^−^ (Figure S74) have been also optimized at the PBEh‐3c level of theory. The calculated natural population analysis (NPA) charges, Wiberg bond indices (WBIs), and/or spin densities according to NBO analyses for **4a** (Table S10), **5a** (Table S11), [**6a**]^−^ (Table S12), and [**7a**]^−^ (Table S13) are given in the Supporting Information. The EPR spectra of the radicals **4a** and **5a** (Figure 3, see the Supporting Information for **4b**,**c** and **5b**,**c**) measured at 298 K in toluene exhibit a characteristic EPR signal for *S* = ½ systems. The EPR spectra of **4a**–**c** and **5a**–**c** were successfully simulated using calculated coupling constants (Table S1). For **4b**, **4c**, and **5a**, the EPR spectrum of each exhibits a featureless signal, while the EPR spectra of **4a**, **5b**, and **5c** reveal fine structure. The hyperfine coupling constants (*hfc*) for **4a**–**c** and **5a**–**c** (Table S1) are in good agreement with those calculated for related [(NHC)Ar] radicals.^[^ ^63^ ^]^

**Figure 4:**
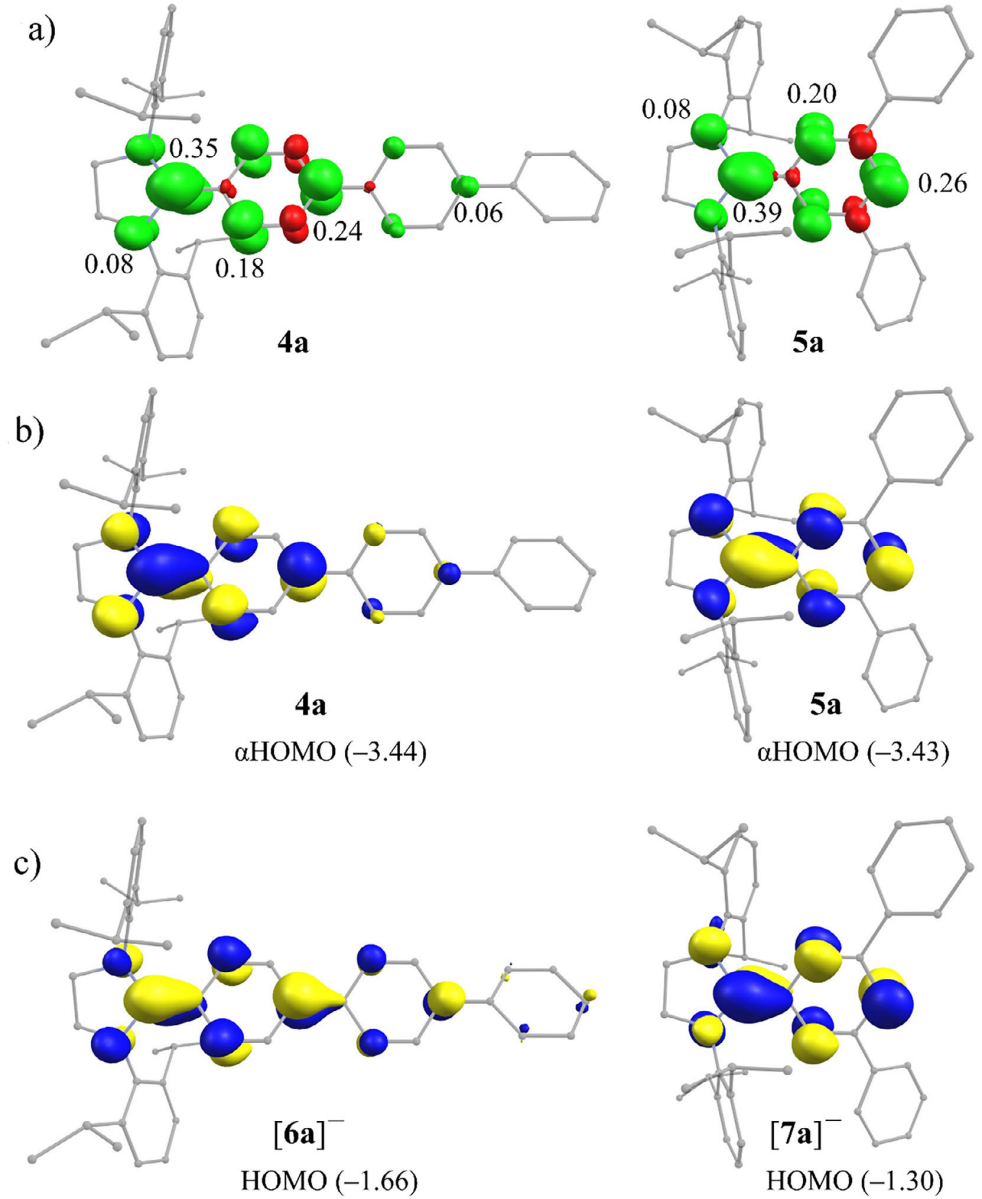
a) Calculated spin density plots (isosurfaces ± 0.005 a. e.) for **4a** and **5a**. The numbers correspond to natural spin density on the atoms. b) Plots (isosurfaces 0.05 a. u.) of the αHOMO (energy in eV) for **4a** and **5a**. c) Plots (isosurfaces 0.05 a. u.) of the HOMO of [**6a**]^−^ and [**7a**]^−^ [PBE0/def2‐TZVP].

The plots of calculated spin density distribution for **4a** and **5a** (Figure 4a) show that the unpaired electron is mostly localized at the C_NHC_‐carbon atom (0.35 *e* for **4a** and 0.39 *e* for **5a**) and the *ortho*‐ and *para*‐carbon atoms of the C_NHC_‐attached aryl ring (0.18–0.26 *e*) of the *p*‐Ter or *m*‐Ter unit. For **4a**, a small part of the spin density is also found at the *o*‐ and *p*‐carbon atoms (∼ 0.06 *e* each) of the central C_6_H_4_ ring, while this is virtually lacking at the terminal aryl ring (*p*‐C_6_H_5_) of the *p*‐Ter unit. Thus, the extent of delocalization of the unpaired electron in **4a** is comparable to that observed in [(NHC)Bp] radicals.^[^ ^75^ ^]^ Also, no spin density is found at the C_6_H_5_ rings of the *m*‐Ter unit. Nonetheless, the presence of an additional (electronegative) *p*‐phenyl group lowers the energy of the αHOMO (i.e., SOMO = singly occupied molecular orbital, in other notion) of **4a** (−3.44 eV, Figure 4b) relative to that of [(NHC)Bp] radicals (−2.61 to −2.81 eV, Bp = *p*‐(C_6_H_5_)C_6_H_4_) as well as of [(NHC)Ar] (−2.40 to −2.67 eV) (Ar = mono‐phenyl substituent).^[^ ^63^ ^]^ Notably, the αHOMO of **5a** (−3.43 eV a) is also stabilized to a similar extent, which may be rationalized considering the negative inductive effect (–*I*) of the *m*‐phenyl groups on the C_6_H_3_‐ring. The αHOMO of **4a** and **5a** (Figure 4b) reveals an out‐of‐phase combination of the *p*‐orbital at the C_NHC_ (or C1, see Table 1 for atom numbering) with the adjacent nitrogen lone pair orbitals, while there is an in‐phase combination between the *p*‐orbital of C1 and C4 atoms. This is in good agreement with the partial C1–C4 double bond character (cf. WBI for **4a** = 1.30, **5a** = 1.28 and for **2a** = 1.07, **3a** = 1.06) (Table 2). A similar orbital topology for the HOMO (highest occupied molecular orbital) of anions [**6a**]^−^ and [**7a**]^−^ can be seen (Figure 4c), which is in line with the population of the αHOMO of **4a** and **5a** with an additional electron to give [**6a**]^−^ and [**7a**]^−^. Consequently, the C1–C4 double bond character further increases in [**6a**]^−^ (WBI = 1.50) and [**7a**]^−^ (WBI = 1.48) as revealed by the respective WBI (Table 2). Moreover, the calculated NPA charges show the negative charge in anions largely resides on the terphenyl substituent.

**Table 2:** Selected Natural charges (*q*) and Wiberg Bond Indices (WBIs) for **2a**, **3a**, **4a**, and **5a**.

	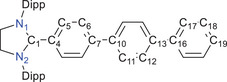		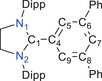	
Compound	**2a**	**4a**	**3a**	**5a**
Atom	**NBO charge (*q*)**			
N1/C1	−0.36/0.58	−0.43/0.40	−0.35/0.58	−0.43/0.39
C4/C10/C16	−0.17/−0.09/−0.06	−0.19/−0.04/−0.04	−0.15	−0.17
C5	−0.16	−0.21	−0.16	−0.21
C6	−0.20	−0.20	−0.03	−0.05
C7/C13	0.02/−0.01	−0.09/−0.07	−0.15	−0.24

Bond	**WBI**			
N1–C1	1.30	1.10	1.31	1.10
C1–C4	1.07	1.30	1.06	1.28
C4–C5	1.35	1.23	1.37	1.25
C5–C6	1.47	1.54	1.39	1.45
C6–C7	1.36	1.31	1.39	1.33
C7–C10	1.07	1.10	−	−
C13–C16	1.06	1.08	−	−

**Figure 5:**
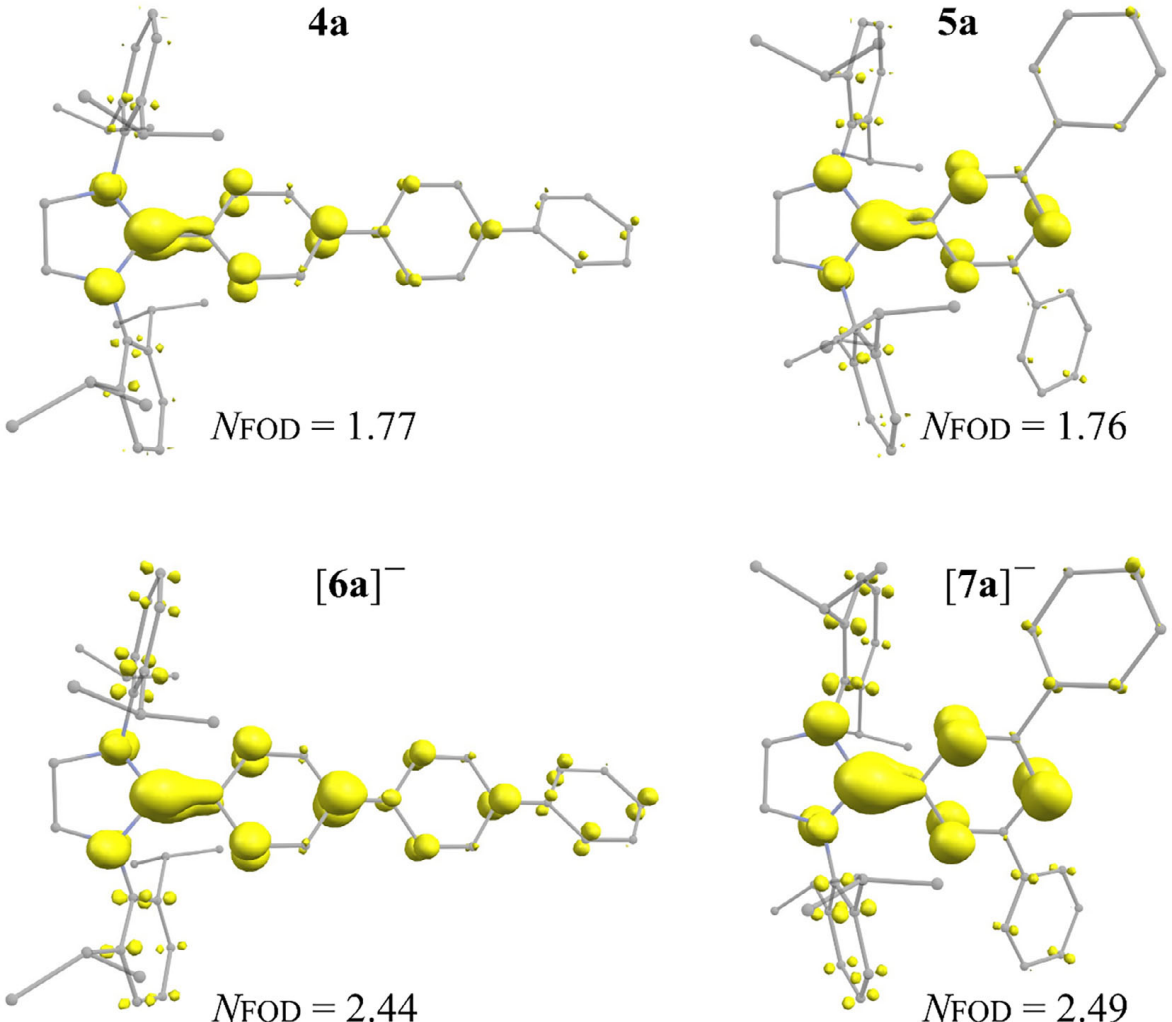
FOD plots (isosurfaces ± 0.005 a. e. in yellow, at the FT‐PBE0/def2‐TZVP level of theory) of **4a**, **5a**, [**6a**]^−^, and. [**7a**]^−^.

We also performed fractional occupation number weighted density (FOD) calculations as an electron correlation diagnostic^[^ ^81^ ^]^ to analyze the electronic structures of **4a**, **5a**, [**6a**]^−^ and [**7a**]^−^ (Figure 5). FOD studies provide reliable information on the localization of “hot” (strongly correlated and chemically active) electrons in a molecule.^[^ ^82^, ^83^, ^84^ ^]^ The FOD plots of **4a**, **5a**, [**6a**]^−^, and [**7a**]^−^ (Figure 5) nicely visualize the “hot” electrons on the C_NHC_‐aryl (i.e. *p*‐Ter or *m*‐Ter) unit and nitrogen atoms. The resulting FOD number *N*_FOD_ increases from the cations ([**2a**]^+^ = 1.19 *e* and [**3a**]^+^ = 1.15 *e*), to the radicals (**4a** = 1.76 *e* and **5a** = 1.77 *e*), and to the anions ([**6a**]^−^ = 2.44 *e*) and [**7a**]^−^ = 2.49 *e*). These *N*_FOD_ values for radicals and anions suggest an intermediate electron correlation.^[^ ^71^, ^72^ ^]^ Based on these results, the stability of the reported radicals (or anions) may be attributed to the delocalization of the unpaired electron (or electrons lone pair) over the C_NHC_‐*m*/*p*‐Ter moiety.

The UV–vis spectra of **4a**–**c** (Figures S41–S43) and **5a**–**c** (Figures S44–S46) exhibit three main absorption bands in the 400–800 nm region. According to the TD‐DFT calculations, the absorption band at *λ*_max._ (in nm) = 707 (**4a**), 801 (**4b**) or 780 (**4c**) may be assigned to the αHOMO→αLUMO transition, while the higher energy band at *λ*_max._ (in nm) = 435 (**4a**), 474 (**4b**), or 484 (**4c**) corresponds to βHOMO → βLUMO and αHOMO–1 → αLUMO transitions (Table S14). Similarly, the absorption band at *λ*_max._ (in nm) = 401 (**5a**), 448 (**5b**) or 458 (**5c**) is related to αHOMO → αLUMO + 1/αLUMO + 2 and βHOMO–2 → βLUMO transitions (Table S15).

The radicals **4a**–**c** and **5a**–**c** are stable at room temperature under an inert gas atmosphere for months. However, the anions **6a**–**c‐K** and **7a‐K** oxidize slowly to form the corresponding radicals even under nitrogen (or argon) atmosphere (∼ 5 ppm O_2_). The radicals are also thermally stable in the solid‐state up to (decomposition temperature) 108 (**4a**), 91 (**4b**), 88 (**4c**), 99 (**5a**), 82 (**5b**), or 80 °C (**5c**). Thus, the SIPr‐based radicals **4a** and **5a** are thermally more stable than IPr‐ and Me‐IPr‐derived radicals (**4b**,**c** and **5b**,**c**). Furthermore, the *p*‐Ter‐derivatives **4a**–**c** decompose at a relatively higher temperature in the solid‐state than their corresponding *m*‐Ter‐analogues **5a**–**c**. Interestingly, in solution, the decomposition trend is reversed. A green benzene solution of **4b** turned completely pale yellow after 1 h at 80 °C, whereas no color change was observed for **5b** under similar conditions. The color change from green to yellow (decomposition) of **5b** occurred at 100 °C after 1 h.

**Scheme 4:**
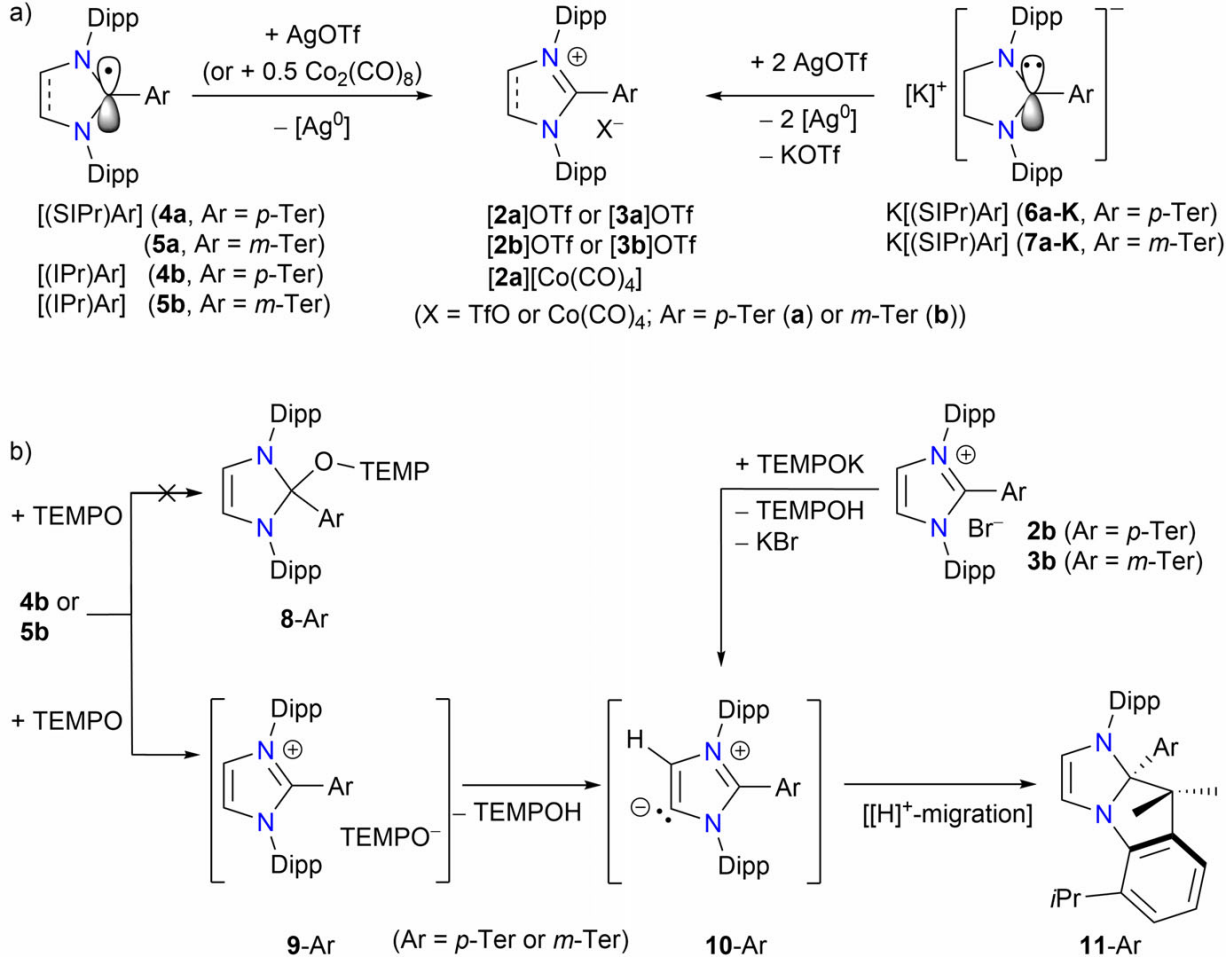
a) Oxidations of radicals (**4a**–**c**, **5a**–**c**) and anions (**6a‐K**, **7a‐K**) with AgOTf or Co_2_(CO)_8_. b) Reactions of **4b** and **5b** with TEMPO to **11**‐Ar.

In line with the CVs of **2a**–**c** and **3a**–**c**, the radicals **4a**–**c** and **5a**–**c** undergo 1e‐oxidation with AgOTf to quantitatively yield the corresponding cations [**2a**–**c**]^+^ and [**3a**–**c**]^+^ with a triflate counter anion (Scheme 4a). Also, two equivalents of AgOTf are required to generate [**2a**]^+^ and [**3a**]^+^ from **6a‐K** and **7a‐K**, while the use of one equivalent AgOTf gave **4a** and **5a**, respectively. Treatment of **4a** with Co_2_(CO)_8_ also affords the diamagnetic salt [**2a**][Co(CO)_4_], in which Co(0) is formally reduced to Co(–I). The ^1^H and ^13^C NMR spectra of [**2a**][Co(CO)_4_] exhibit characteristic signals for the [**2a**]^+^ moiety and has been also characterized by sc‐XRD (Figure S68).

Among stable radicals, nitroxyl radicals such as TEMPO (2,2,6,6‐Tetramethylpiperidinyloxyl) featuring three‐electron‐two‐center π double bond have found widespread applications in various chemical and electrochemical oxidations, polymerizations, and biological antioxidant processes.^[^ ^85^, ^86^, ^87^, ^88^, ^89^, ^90^ ^]^ This is owing to their unique properties and reactivity.^[^ ^91^ ^]^ However, 1e‐reduction of TEMPO and related species to the corresponding anions has received rather limited attention.^[^ ^92^, ^93^, ^94^ ^]^ To further probe the chemical reactivity of the radicals, we treated **4b** and **5b** with one equivalent of TEMPO radical, which resulted in each case a discoloration (green to light brown). The well‐resolved ^1^H NMR spectrum of each sample revealed the selective formation of a diamagnetic species, i.e., **11**‐Ar (Scheme 4b) instead of the expected radical‐coupling products **8**‐Ar. The exact mechanism of the formation of **11**‐Ar is unknown. One‐electron oxidation of **4b** or **5b** with TEMPO to form the salt [(NHC‐Ar)](TEMPO) (**9**‐Ar) is likely.^[^ ^95^ ^]^ In **9**‐Ar, the deprotonation of the cation [(NHC‐Ar)]^+^ by the counter anion TEMPO^−^ in resulting the mesoionic carbene (*i*MIC) **10**‐Ar seems plausible, which subsequently undergo proton migration via a C_sp3_–H bond activation to ultimately form **11**‐Ar. This type of transformation is known for *i*MICs.^[^ ^96^ ^]^ Notably, TEMPO is known to react with an alkali metal (M) reducing agent to afford (TEMPO)M,^[^ ^97^, ^98^ ^]^ which show nucleophilic reactivity.^[^ ^99^ ^]^ Indeed, the deprotonation of **2b** and **3b** by TEMPOK (or KN(SiMe_3_)_2_) yields **10**‐Ar, which at elevated temperature form **11**‐Ar (see Figures S1–S4). For **11**‐Ar, the ^1^H NMR spectra show three septets for the methine protons (*H*CMe_2_), a set of several doublets for methyl protons (HC*Me*_2_), and two singlets for the methyl groups (C*Me*_2_). In addition, the ^1^H NMR spectra of **11**‐Ar exhibit two distinct singlets for the backbone NC*H* protons, indicating that they are magnetically different. In addition to other expected signals, the ^13^C{^1^H} NMR spectra of **11**‐Ar show characteristic resonances for the CMe_2_ unit (*Me*: 1.0/31.1; *C*: 101.3 ppm). The molecular structures of **11**‐Ar (Figures S66 and S67) determined by sc‐XRD clearly show the formation of C–H activation (i.e., C–C coupling) products, which align well with their NMR data.

## Conclusions

In conclusion, we have reported the synthesis and structural characterization of two series of NHC–terphenyl systems, [(NHC)*p*‐Ter] and [(NHC)*m*‐Ter], based on three distinct NHCs (SIPr, IPr, and Me‐IPr). Electrochemical studies confirm the accessibility of these systems in three redox states: cationic [(NHC)Ar]^+^, radical [(NHC)Ar]^●^, and anionic [(NHC)Ar]^−^ species (Ar = *p*‐Ter or *m*‐Ter). The radicals [(NHC)*p*‐Ter]^●^ (**4a**–**c**) and [(NHC)*m*‐Ter]^●^ (**5a**–**c**) have been isolated as stable crystalline solids and fully characterized by sc‐XRD, EPR, and UV–vis spectroscopy. This is in line with the CVs of [(NHC)Ar]Br, which show a reversible redox wave for the [(NHC)Ar]^+^/[(NHC)Ar]^●^ couple. The second reduction step is reversible only for the *p*‐Ter‐derivatives, i.e., the ([(NHC)*p*‐Ter]^●^/[(NHC)*p*‐Ter]^−^ redox couple, enabling the isolation of anions K[(NHC)*p*‐Ter] (**6a**–**c‐K**) as stable diamagnetic solids as confirmed by NMR spectroscopy. For the *m*‐Ter‐series, the second reduction is irreversible or quasi‐reversible. The diamagnetic anion K[(SIPr)*m*‐Ter] (**7a‐K**) is obtained by reducing radical **5a** and characterized by NMR, whereas attempts to reduce the radicals [(IPr)*m*‐Ter]^●^ (**5b**) or [(Me‐IPr)*m*‐Ter]^●^ (**5c**) under similar conditions were not viable.

Reactivity studies of the radicals with TEMPO yielded C–H activation products (**11**‐Ar), while reactions of the radicals or anions with AgOTf quantitatively generated the corresponding cations or radicals. Overall, these findings highlight the crucial influence of the C2‐terphenyl substituent, in addition to the NHC identity, on the stability and redox behavior of NHC‐based systems. The insights gained here provide valuable information for designing new redox‐active systems with tunable and predictable properties.

## Supporting Information

Experimental details, the plots of NMR, EPR, and UV–vis spectra, the details of X‐ray crystallography, and quantum chemical calculations of the reported compounds are given in the Supporting Information.

## Conflict of Interests

The authors declare no conflict of interest.

## Supporting information



Supporting Information

Supporting Information

## Data Availability

The data that support the findings of this study are available in the Supporting Information of this article.
