# Supplementary material for: NHC‐Terphenyl Radicals and Anions: Tuning Stability and Redox Properties via Substituent Patterning
Source: Angew Chem Int Ed Engl. 2025 Oct 22;64(51):e202520260. doi: 10.1002/anie.202520260 (PMC12707353; doi:10.1002/anie.202520260)
Supplement: Supplementary file 1 — Supporting Information [file ANIE-64-e202520260-s002.pdf]

Supporting Information

for

NHC-Terphenyl Radicals and Anions: Tuning Stability and Redox Properties via Substituent Patterning

Henric Steffenfauseweh,[a] Yury V. Vishnevskiy,[a] Beate Neumann,[a] Hans-Georg Stammler,[a] Bas de Bruin,[b] and Rajendra S. Ghadwal[a] *

[a]Molecular Inorganic Chemistry and Catalysis, Inorganic and Structural Chemistry, Center for Molecular Materials, Faculty of Chemistry, Universität Bielefeld, Universitätsstrasse 25, D-33615, Bielefeld, Germany

**E-Mail: rghadwal@uni-bielefeld.de; http://www.ghadwalgroup.de*

Fax: +49 521 106 6026; Tel: +49 521 106 6167

[b]University of Amsterdam (UvA), Faculty of Science, Van 't Hoff Institute for Molecular Sciences (HIMS), Homogeneous and Supramolecular Catalysis Group, Science Park904, 1098 XH Amsterdam, The Netherlands

Table of Contents

Materials and Method S1

Synthesis of [(SIPr)*p*-Ter]Br (2a) and [(SIPr)*m*-Ter]Br (3a) S1

Synthesis of [(IPr)*p*-Ter]Br (2b) and [(IPr)*m*-Ter]Br (3b) S2

Synthesis of [(Me-IPr)*p*-Ter]Br (2c) and [(Me-IPr)*m*-Ter]Br (3c) S3

Synthesis of Radicals [(NHC)*p*-Ter] (4a–c) S4

Synthesis of Radicals [(NHC)*m*-Ter] (5a–c) S5

Synthesis of K[(NHC)*p*-Ter] (6a-K) and (6c-K) S6

Synthesis of K[(SIPr)*m*-Ter] (7a-K) S7

Reactions of 4b and 5b with TEMPO S8

NMR Monitoring of the Reactions of 4b with TEMPO S9

One-electron Oxidation Reactions of Radicals S12

Plots of NMR Spectra S14

UV-Visible Spectra S34

Cyclic Voltammetry S39

EPR Spectroscopy S42

Crystallographic Details S46

Computational Details S59

References S82

Materials and Method

All syntheses and manipulations were carried out under an inert gas (Ar or N_2) atmosphere using standard *Schlenk* techniques or a glove-box (MBraun LABMasterPro). Organic solvents were dried over appropriate drying agents, distilled, and stored over 3 Å molecular sieve. Nano-ESI mass spectra were recorded using an Esquire 3000 ion trap mass spectrometer (Bruker Daltonik GmbH, Bremen, Germany) equipped with a nano-ESI source. Samples were dissolved in THF or dichloromethane (for **4**) and introduced by static nano-ESI using in-house pulled glass emitters. Nitrogen served both as nebulizer as well as dry gas and was generated by a Bruker nitrogen generator NGM 11. Helium served as cooling gas for the ion trap. The mass axis was externally calibrated with ESI-L Tuning Mix (Agilent Technologies, Santa Clara, CA, USA) as a calibration standard. Melting points (MPs) were measured using a Büchi B-545 melting point apparatus. Unless stated otherwise, elemental analyses of reported compounds were performed from isolated samples without further purifications. Deuterated solvents were dried over appropriate drying agents, distilled, and stored inside a glove box. NMR spectra were recorded on a Bruker Avance III 500 or a Bruker Avance III 500 HD spectrometer. Chemical shifts (in δ, ppm) are referenced to the residual solvent signal(s): CD_2Cl_2 (^{1}H, 5.32; ^{13}C, 53.84), CD_3CN (^{1}H, 1.94; ^{13}C, 1.32), C_6D_6 (^{1}H, 7.16; ^{13}C, 128.06), and THF-d_8 (^{1}H, 3.58; ^{13}C, 67.21 ppm).[1] IPr (**1**a), SIPr (**1**b), Me-IPr (**1**c),[2] Ni(cod)$_2$,[3] KC_8,[4] TEMPOK and TEMPOH were prepared according to literature protocols.[5] 4-Bromo-*p*-terphenyl (*p*-TerBr) and 5'-bromo-*m*-terphenyl (*m*-TerBr) (BLD Pharma); TEMPO (Aldrich) and AgOTf (Fluorochem) were used as received from the supplier.

Synthesis of [(SIPr)*p*-Ter]Br (2a) and [(SIPr)*m*-Ter]Br (3a)

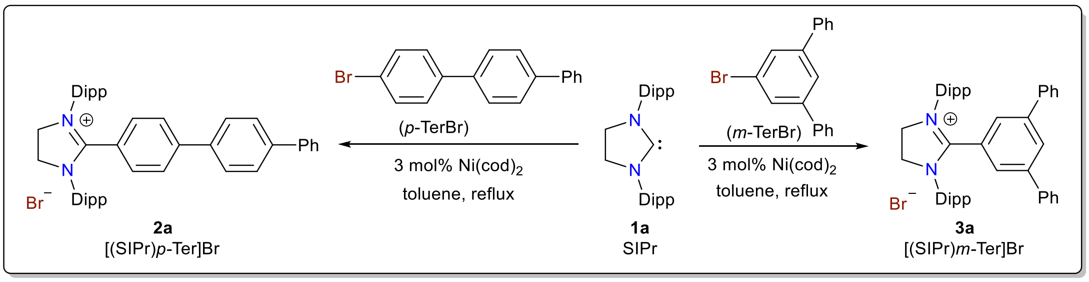

Synthesis of [(SIPr)*p*-Ter]Br (2a): A toluene (20 mL) solution of **1a** (2.50 g, 6.4 mmol), *p*-TerBr (1.98 g, 6.4 mmol), and Ni(cod)$_2$ (0.05 g, 0.2 mmol) was refluxed for 3 h at 140 °C (oil bath temperature) to yield a white suspension. The precipitate was isolated by filtration, washed with 20 mL toluene and then with ethyl acetate (3x20 mL), and dried in vacuum at 120 °C to afford compound **2a** as a colorless solid in 69% (3.10 g) yield. Single crystals suitable for X-ray diffraction (sc-XRD) were obtained by slow diffusion of ethyl acetate into a saturated dichloromethane solution of **2a** at rt. **MP**: 256-260 °C. Elemental analyses (%) calculated for $C_{45}H_{51}N_2Br$ (699.82) (**2a**): C 77.23, H 7.35, N 4.00; found: C 75.80, H 7.29, N 3.74. **ESI** (m/z) observed (calculated) [Fragment]: 619.399 (619.404) [**2a**–Br]$^+$. ^{1}**H**

NMR (500 MHz, $CDCl_3$, 298 K): 7.58 (d, *J* = 8.4 Hz, 2H, C_6H_4), 7.53 (d, *J* = 7.4 Hz, 2H, C_6H_4), 7.50 (d, *J* = 7.9 Hz, 2H, C_6H_4), 7.43 (q, *J* = 7.9 Hz, 6H, C_6H_4 and *o*-, *m*-C_6H_5), 7.33 (t, *J* = 7.3 , 1H, *p*-C_6H_5), 7.22 (d, *J* = 7.8 Hz, 4H, *m*-C_6H_3), 7.08 (m, 2H, *p*-C_6H_3), 4.91 (s, 4H, NCH_2), 3.07 (sept, *J* = 6.9 Hz, 4H, $CH(CH_3)_2$), 1.42 (d, *J* = 6.6 Hz, 12H, $CH(CH_3)_2$), 1.02 (d, *J* = 6.7 Hz, 12H, $CH(CH_3)_2$) ppm. **$^{13}C\{^1H\}$ NMR** (125 MHz, $CDCl_3$, 298 K): 166.0 (NCN), 146.0 (*p*-Ter), 145.6 (*ipso*-C_6H_3), 142.2 (*p*-Ter), 139.9 (*p*-Ter), 136.5 (*p*-Ter), 131.4 (*o*-C_6H_3), 131.3 (*p*-Ter), 130.1 (*p*-C_6H_3), 129.0 (*p*-Ter), 128.0 (*p*-Ter), 127.8 (*p*-C_6H_5), 127.5 (*p*-Ter), 127.1 (*p*-Ter), 126.8 (*p*-Ter), 125.6 (*m*-C_6H_3), 119.6 (*p*-Ter), 55.1 (NCH_2), 29.3 ($CH(CH_3)_2$), 26.1 ($CH(CH_3)_2$), 23.3 ($CH(CH_3)_2$) ppm.

Synthesis of [(SIPr)*m*-Ter]Br (3a): A similar method as described above for **2a** was employed to prepare **3a**, using **1a** (2.50 g, 6.4 mmol), *m*-TerBr (1.99 g, 6.4 mmol), and Ni(cod)$_2$ (0.05 g, 0.2 mmol), as a colorless solid in 61% (2.72 g) yield. Crystals suitable for sc-XRD were obtained by slow diffusion of ethyl acetate into a saturated dichloromethane solution of **3a** at rt. **MP**: 259-263 °C. Elemental analyses (%) calculated for $C_{45}H_{51}N_2Br$ (699.82) (**3a**): C 77.23, H 7.35, N 4.00; found: C 75.63, H 7.34, N 3.94. **ESI** (m/z) observed (calculated) [Fragment]: 619.380 (619.404) [**3a**–Br]$^+$. **^1H NMR** (500 MHz, $CDCl_3$, 298 K): 7.69 (s, 1H, *p*-C_6H_3 of *m*-Ter), 7.51 (t, *J* = 7.8 Hz, 2H, *p*-C_6H_3 of Dipp), 7.35–7.32 (m, 6H, *o*-, *p*-C_6H_5 of *m*-Ter), 7.29 (d, *J* = 7.8 Hz, 4H, *m*-C_6H_3 of Dipp), 7.16 (s, 2H, *o*-C_6H_3 of *m*-Ter), 6.96–6.91 (m, 4H, *o*-C_6H_5), 4.98 (s, 4H, NCH_2), 3.09 (sept, *J* = 6.9 Hz, 4H, $CH(CH_3)_2$), 1.41 (d, *J* = 6.7 Hz, 12H, $CH(CH_3)_2$), 0.92 (d, *J* = 6.7 Hz, 12H, $CH(CH_3)_2$) ppm. **$^{13}C\{^1H\}$ NMR** (125 MHz, $CDCl_3$, 298 K): 165.9 (NCN), 145.9 (*o*-C_6H_3 of Dipp), 143.1 (*ipso*-C_6H_5), 138.4 (*m*-C_6H_3 of *m*-Ter), 131.6 (*ipso*-C_6H_3 of Dipp), 131.4 (*p*-C_6H_3 of Dipp), 131.2 (*p*-C_6H_3 of *m*-Ter), 129.1, 128.8 (*m*- and *p*-C_6H_5), 127.3, 127.1 (*o*-C_6H_5), 125.8 (*m*-C_6H_3 of Dipp), 122.0 (*ipso*-C_6H_3 of *m*-Ter), 55.2 (NCH_2), 29.4 ($CH(CH_3)_2$), 26.2 ($CH(CH_3)_2$), 23.2 ($CH(CH_3)_2$) ppm.

Synthesis of [(IPr)*p*-Ter]Br (2b) and [(IPr)*m*-Ter]Br (3b)

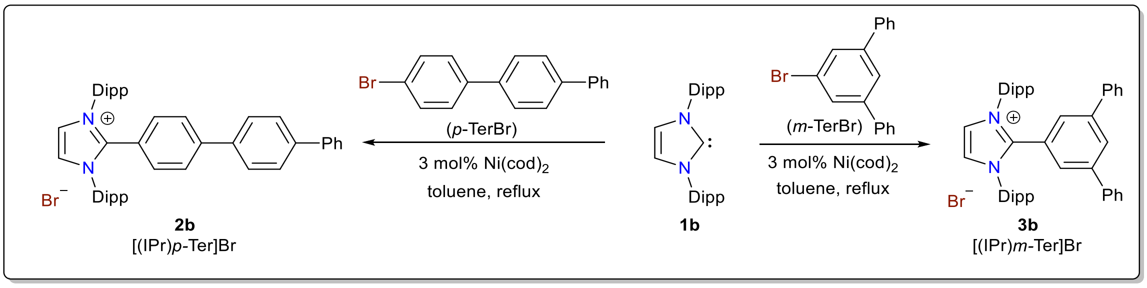

Synthesis of ([IPr)*p*-Ter]Br (2b): A similar method as discussed above for **2a** was used to prepare **2b**, using **1b** (2.99 g, 7.7 mmol), *p*-TerBr (2.39 g, 7.7 mmol), and Ni(cod)$_2$ (0.09 g, 0.3 mmol), as a colorless solid in 87% (4.67 g) yield. Crystals suitable for sc-XRD were obtained by slow diffusion of ethyl acetate into a saturated dichloromethane solution of **2b** at rt. **MP**: 251-256 °C. Elemental analyses (%)

calculated for $C_{45}H_{49}N_2Br$ (697.81) (**2b**): C 77.46, H 7.08, N 4.01; found: C 76.24, H 7.31, N 3.90. **ESI** (m/z) observed (calculated) [Fragment]: 617.378 (617.389) [**2b**–Br]$^+$. **^{1}H NMR** (500 MHz, $CDCl_3$, 298 K): 8.56 (s, 2H, NC*H*), 7.59 (t, *J* = 7.7 Hz, 2H, *m*-C_6H_5), 7.56 (d, *J* = 8.1 Hz, 4H, C_6H_4), 7.52 (d, *J* = 8.4 Hz, 2H, C_6H_4), 7.48 (d, *J* = 8.7 Hz, 2H, C_6H_4), 7.41 (t, *J* = 7.9 Hz, 2H, *p*-C_6H_3), 7.33 (t, *J* = 7.7 Hz, 1H, *p*-C_6H_5), 7.31 (d, *J* = 7.9 Hz, 4H, *m*-C_6H_3), 6.96 (d, *J* = 8.4 Hz, 2H, C_6H_4), 2.46 (sept, *J* = 6.9 Hz, 4H, C*H*(CH_3)$_2$), 1.31 (d, *J* = 6.7, 12H, CH(CH_3)$_2$), 1.01 (d, *J* = 6.9, 12H, CH(CH_3)$_2$) ppm. **^{13}C{^{1}H} NMR** (125 MHz, $CDCl_3$, 298 K): 144.8 (N*C*N), 144.7 (*ipso*-C_6H_4), 144.3 (*ipso*-C_6H_3), 142.0 (*ipso*-C_6H_4), 139.9 (*ipso*-C_6H_4), 136.4 (*ipso*-C_6H_5), 132.4 (C_6H_4), 130.2 (*ipso*-C_6H_3), 129.6 (C_6H_4), 129.0 (*p*-C_6H_3), 127.9 (NC*H*), 127.8 (*m*-C_6H_5), 127.4 (C_6H_4), 127.3 (C_6H_4), 127.1 (*p*-C_6H_5), 125.4 (*m*-C_6H_3), 119.2 (*ipso*-C_6H_4), 29.4 (*C*H(CH_3)$_2$), 25.6 (CH(*C*H_3)$_2$), 22.7 (CH(*C*H_3)$_2$) ppm.

Synthesis of [(IPr)*m*-Ter]Br (3b): A similar method as described above for **2a** was used to prepare **3b**, employing **1b** (2.99 g, 7.7 mmol), *m*-TerBr (2.40 g, 7.7 mmol), and Ni(cod)$_2$ (0.08 g, 0.3 mmol), as a colorless solid in 45% (2.42 g) yield. **MP**: 254-256 °C. Elemental analyses (%) calculated for $C_{45}H_{49}N_2Br$ (697.81) (**3b**): C 77.46, H 7.08, N 4.01; found: C 75.28, H 7.32, N 3.90. **ESI** (m/z) observed (calculated) [Fragment]: 617.369 (617.389) [**3b**–Br]$^+$. **^{1}H NMR** (500 MHz, $CDCl_3$, 298 K): 8.71 (s, 2H, NC*H*), 7.72 (br. s, 1H, *p*-C_6H_3 of *m*-Ter), 7.65 (t, *J* = 7.8 Hz, 2H, *p*-C_6H_3 of Dipp), 7.40 (d, *J* = 7.9 Hz, 4H, *m*-C_6H_3 of Dipp), 7.35 (m, 6H, *m*- and *p*-C_6H_5), 7.08 (br. s, 2H, *o*-C_6H_3 of *m*-Ter), 6.97 (m, 4H, *o*-C_6H_5), 2.50 (sept, *J* = 6.9 Hz, 4H, C*H*(CH_3)$_2$), 1.33 (d, *J* = 6.7 Hz, 12H, CH(CH_3)$_2$), 0.93 (d, *J* = 6.8 Hz, 12H, CH(CH_3)$_2$) ppm. **^{13}C{^{1}H} NMR** (125 MHz, $CDCl_3$, 298 K): 145.1 (*ipso*-C_6H_3 of Dipp), 144.1 (N*C*N), 143.2 (*ipso*-C_6H_5), 138.5 (*m*-C_6H_3 of *m*-Ter), 132.3 (*p*-C_6H_3 of Dipp), 130.6 (*o*-C_6H_3 of Dipp), 129.8 (*p*-C_6H_3 of *m*-Ter), 129.2 (C_6H_5), 128.8 (C_6H_5), 128.2 (N*C*H), 127.0 (*o*-C_6H_3 of *m*-Ter), 126.7 (*o*-C_6H_5), 125.6 (*m*-C_6H_3 of Dipp), 121.6 (*ipso*-C_6H_3 of *m*-Ter), 29.5 (*C*H(CH_3)$_2$), 25.7 (CH(*C*H_3)$_2$), 22.7 (CH(*C*H_3)$_2$) ppm.

Synthesis of [(Me-IPr)*p*-Ter]Br (2c) and [(Me-IPr)*m*-Ter]Br (3c)

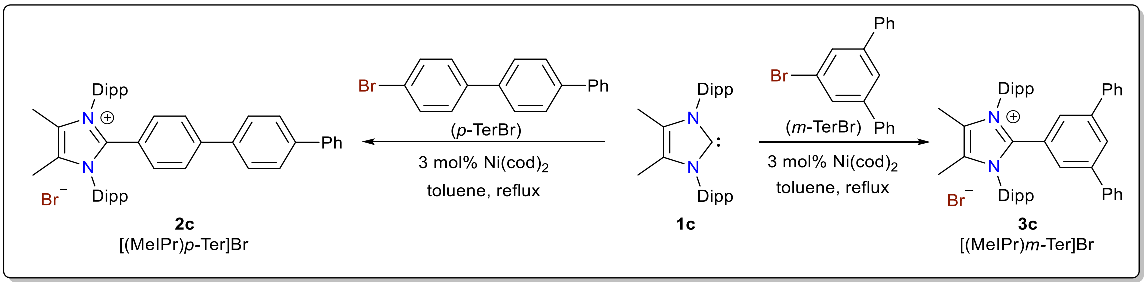

Synthesis of [(Me-IPr)*p*-Ter]Br (2c): A similar method as described above for **2a** was used to prepare **2c**, using **1c** (3.04 g, 7.2 mmol), *p*-TerBr (2.18 g, 7.1 mmol), and Ni(cod)$_2$ (0.08 g, 0.3 mmol), as a colorless solid in 73% (3.74 g) yield. Crystals suitable for sc-XRD were obtained by slow diffusion of ethyl acetate into a saturated dichloromethane solution of **2**c at rt. **MP**: 233-237 °C. Elemental analyses

(%) calculated for $C_{47}H_{53}N_2Br$ (725.86) (**2c**): C 77.77, H 7.36, N 3.86; found: C 76.24, H 7.41, N 3.74. **ESI** (m/z) observed (calculated) [Fragment]: 645.423 (645.420) [**2c**–Br]$^+$. **^{1}H NMR** (500 MHz, $CDCl_3$, 298 K): 7.63 (m, 4H, *p*-C_6H_3, *m*-C_6H_5), 7.58 (d, *J* = 7.8 Hz, 2H, C_6H_4), 7.54 (d, *J* = 7.8 Hz, 2H, C_6H_4), 7.41 (m, 9H, *p*-C_6H_5 (1H), C_6H_4 (4H), *m*-C_6H_3 (4H)), 7.00 (d, *J* = 8.2 Hz, 2H, *o*-C_6H_5), 2.46 (m, 10H, C*H*$(CH_3)_2$ (4H), NCCH_3 (6H)), 1.32 (d, *J* = 6.8 Hz, 12H, CH(CH_3)$_2$), 1.02 (d, *J* = 6.8 Hz, 12H, CH(CH_3)$_2$) ppm. **^{13}C{^{1}H} NMR** (125 MHz, $CDCl_3$, 298 K): 145.3 (*o*-C_6H_3), 144.2 (*ipso*-C_6H_5), 142.9 (N*C*N), 142.1, 140.1 (C_6H_3, C_6H_5), 136.6 (*ipso*-C_6H_3), 132.7 (*p*-C_6H_3), 130.8 (N*C*CH$_3$), 129.4, 129.1 (C_6H_3, C_6H_5), 128.9 (*p*-/*ipso*-C_6H_4), 128.0, 127.9 (C_6H_3, C_6H_5), 127.4 (*m*-C_6H_5), 127.1, 127.0, 126.1 (C_6H_3, C_6H_5), 120.0 (*p*-/*ipso*-C_6H_4), 29.3 (*C*H$(CH_3)_2$), 25.0 (CH(*C*H$_3$)$_2$), 23.5 (CH(*C*H$_3$)$_2$), 11.0 (NC*C*H$_3$) ppm.

Synthesis of [(Me-IPr)*m*-Ter]Br (3c): Using a similar method as described above for **2a** was used to prepare **3c**, using **1c** (3.04 g, 7.2 mmol), *m*-TerBr ((2.22 g, 7.2 mmol), and Ni(cod)$_2$ (0.08 g, 0.3 mmol), as a colorless solid in 54% (2.79 g) yield. **MP**: 240-243 °C. **ESI** (m/z) observed (calculated) [Fragment]: 645.412 (645.420) [**3c**–Br]$^+$. **^{1}H NMR** (500 MHz, $CDCl_3$, 298 K): 7.80 (t, *J* = 7.8Hz, 2H, *p*-C_6H_3 of Dipp), 7.78 (s, 1H, *p*-C_6H_3 of *m*-Ter), 7.55 (d, *J* = 7.8 Hz, 4H, *m*-C_6H_3 of Dipp), 7.37 (m, 6H, C_6H_5), 7.18 (s, 2H, *o*-C_6H_3 of *m*-Ter), 6.98 (m, 4H, C_6H_5), 2.48 (sept, *J* = 6.8 Hz, 4 H, (C*H*)CH$_3$), 2.43 (s, 6H, NCCH_3), 1.34 (d, *J* = 6.8 Hz, 12 H, (CH)CH_3), 0.94 (d, *J* = 6.8 Hz, 12 H, (CH)CH_3) ppm. **^{13}C{^{1}H} NMR** (125 MHz, $CDCl_3$, 298 K): 145.8 (*o*-C_6H_3 of Dipp), 143.4 (N*C*N), 138.9 (*m*-C_6H_3 of *m*-Ter), 133.3 (*p*-C_6H_3 of Dipp), 130.8 (N*C*CH$_3$), 130.2 (*p*-C_6H_3 of *m*-Ter), 129.5 (*ipso*-C_6H_3 of *m*-Ter), 129.4 (C_6H_5), 129.1 (C_6H_5), 127.5 (C_6H_5), 127.0 (*m*-C_6H_3 of Dipp), 126.8 (*o*-C_6H_3 of *m*-Ter), 125.5 (*ipso*-C_6H_3 of Dipp), 122.4 (*ipso*-C_6H_5), 29.9 (*C*H$(CH_3)_2$), 25.3 (CH(*C*H$_3$)$_2$), 23.5 (CH(*C*H$_3$)$_2$), 11.4 (NC*C*H$_3$) ppm.

Synthesis of Radicals [(NHC)*p*-Ter] (4a–c)

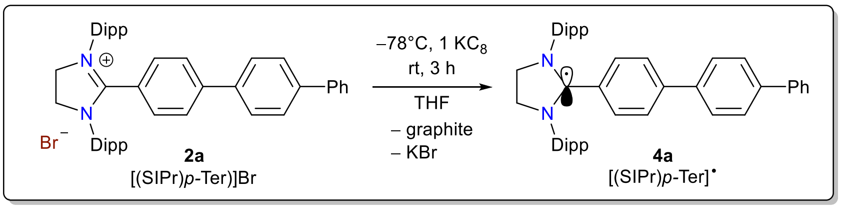

Synthesis of [(SIPr)*p*-Ter)] (4a): THF (10 mL) was added to a precooled (−78 °C) mixture of **2a** (400 mg, 0.57 mmol) and KC_8 (100 mg, 0.73 mmol). The suspension immediately turned green, which was allowed to reach room temperature and stirred overnight. The resulting suspension was filtered and the residue was extracted with THF (2 x 5 mL). The volatiles of the filtrate were removed under vacuum to yield **4a** as a green crystalline solid in 72% yield (255 mg). Crystals suitable for sc-XRD were obtained by slow evaporation of a concentrated *n*-hexane solution of **4a**. **MP**: 108 °C (dec.). **ESI** (m/z): observed

(calculated) [Fragment]: 619.399 (619.404) [**4a**]$^+$. **UV-Vis**. ($\lambda_{max.}$ (nm) (ε (L mol^{-1} cm^{-1}))): 298 (17000); 435 (1900); 707 (600).

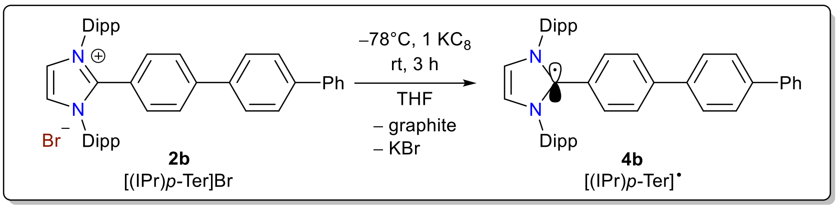

Synthesis of [(IPr)*p*-Ter] (4b): A similar method as discussed above for **4a** was used to prepare **4b**, using **2b** (410 mg, 0.58 mmol) and KC_8 (93 mg, 0.68 mmol), as a green crystalline solid in 99% yield (357 mg). Crystals suitable for sc-XRD were obtained by slow evaporation of a concentrated *n*-hexane solution of **4b**. **MP**: 91 °C (dec.). **ESI** (m/z): observed (calculated) [Fragment]: 617.378 (617.389) [**4b**]$^+$. **UV-Vis**. ($\lambda_{max.}$ (nm) (ε (L mol^{-1} cm^{-1}))): 259 (6000); 305 (8000); 474 (11000); 801 (5000).

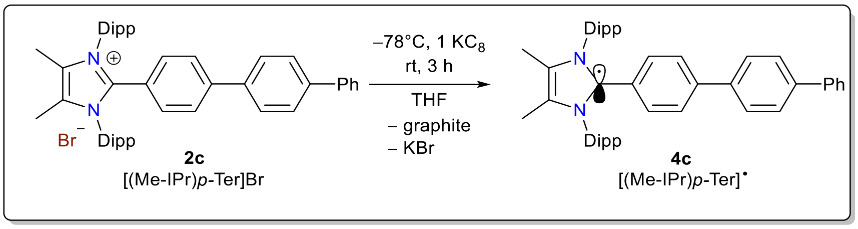

Synthesis of [(Me-IPr)*p*-Ter] (4c): Compound **4c** was prepared employing a similar method as discussed above for **4a** using **2c** (410 mg, 0.58 mmol) and KC_8 (93 mg, 0.68 mmol) as a green crystalline solid in 99% yield (359 mg). Crystals suitable for sc-XRD were obtained by slow evaporation of a concentrated *n*-hexane solution of **4c**. **MP**: 88 °C (dec.). **ESI** (m/z): observed (calculated) [Fragment]: 645.423 (645.420) [**4c**]$^+$. **UV-Vis.** (($\lambda_{max.}$ (nm)): 394 (16000); 772 (600).

Synthesis of Radicals [(NHC)*m*-Ter] (5a–c)

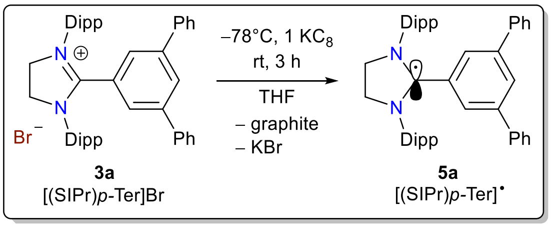

Synthesis of [(SIPr)*m*-Ter)] (5a): Compound **5a** was prepared employing a similar method as discussed above for **4a** using **3a** (198 mg, 0.28 mmol) and KC_8 (48 mg, 0.35 mmol) as a green crystalline solid in 99% yield (174 mg). Crystals suitable for sc-XRD were obtained by slow evaporation of a

concentrated *n*-hexane solution of **5a** at rt. **MP**: 99 °C (dec.). **ESI** (m/z): observed (calculated) [Fragment]: 619.380 (619.404) [**5a**]$^+$. **UV-Vis**. (($\lambda_{max.}$ (nm)): 286 (5000); 401 (1500).

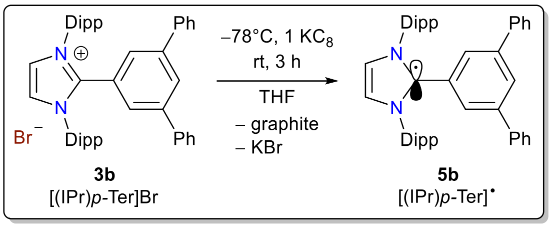

Synthesis of [(IPr)*m*-Ter)] (5b): Compound **5b** was prepared employing a similar method as discussed above for **4a** using **3b** (404 mg, 0.58 mmol) and KC_8 (90 mg, 0.67 mmol) as a green crystalline solid in 84% yield (300 mg). **MP**: 82 °C (dec.). **ESI** (m/z): observed (calculated) [Fragment]: 617.369 (617.389) [**5b**]$^+$. **UV-Vis**. ($\lambda_{max.}$ (nm) (ε (L mol^{-1} cm^{-1}))): 220 (13000); 262 (15000); 448 (3000); 515 (1000).

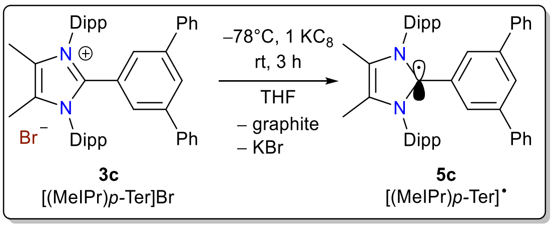

Synthesis of [(Me-IPr)*m*-Ter)] (5c): Compound **5b** was prepared employing a similar method as discussed above for **4a** using **3c** (409 mg, 0.56 mmol) and KC_8 (91 mg, 0.68 mmol) as a green crystalline solid in 85% yield (309 mg). **MP**: 80 °C (dec.). **ESI** (m/z): observed (calculated) [Fragment]: 645.412 (645.420) [M]$^+$. **UV-Vis**. ($\lambda_{max.}$ (nm) (ε (L mol^{-1} cm^{-1}))): 292 (7500); 458 (7500).

Synthesis of K[(NHC)*p*-Ter] (6a-K) and (6c-K)

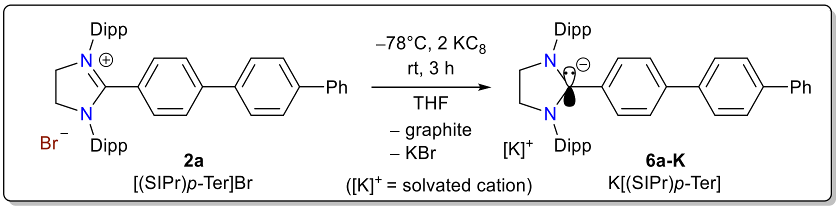

Synthesis of K[(SIPr)p-Ter] (6a-K): THF (10 mL) was added to a precooled (−78 °C) mixture of 2a (212 mg, 0.30 mmol) and KC_8 (110 mg, 0.81 mmol). The suspension turned blue immediately, which was allowed to reach room temperature and further stirred overnight. Filtration through a plug of Celite and extracting the residue with THF (2x 5 mL) gave a blue solution. The volatiles from the filtrate were removed under vacuum to yield **6a-K** as a blue crystalline solid in 91% yield (181 mg, coordinating THF solvent excluded). **^{1}H NMR** (500 MHz, THF-d_8, 298 K): 7.09 (m, 2H, C_6H_5), 7.08 (br, 5H, *m*-C_6H_3

(4H) *p*-C_6H_5 (1H)), 6.93 (t, *J* = 7.8 Hz, 2H, *p*-C_6H_3), 6.72 (d, *J* = 9.0 Hz, 2H, C_6H_4), 6.52 (m, 2H, C_6H_5), 6.09 (d, *J* = 9.0 Hz, 2H, C_6H_4), 5.69 (d, *J* = 9.7 Hz, 2H, C_6H_4), 4.92 (d, *J* = 9.7 Hz, 2H, C_6H_4), 3.72 (sept, *J* = 6.8 Hz, 4H, C*H*(CH_3)$_2$), 3.66 (s, 4H, NCH_2), 1.27 (d, *J* = 6.8 Hz, 12H, CH(CH_3)$_2$), 1.24 (d, *J* = 6.8 Hz, 12H, CH(CH_3)$_2$) ppm. **^{13}C{^{1}H} NMR** (126 MHz, THF-d_8, 298 K): 147.7 (*o*-C_6H_3), 144.2 (*ipso*-C_6H_3), 141.9 (N*C*N), 132.7 (*ipso*/*p*-C_6H_4), 132.5 (*ipso*/*p*-C_6H_4), 128.9 (*p*-C_6H_3), 126.6 (*p*-C_6H_5), 125.8 (C_6H_4), 124.5 (*m*-C_6H_3), 121.3 (C_6H_5), 120.9 (C_6H_4), 119.9 (C_6H_5), 113.9 (*ipso*-C_6H_5), 113.1 (C_6H_4), 112.6 (C_6H_4), 101.9 (*ipso*/*p*-C_6H_4), 101.2 (*ipso*/*p*-C_6H_4), 54.4 (NCH_2), 28.9 (*C*H(CH_3)$_2$), 26.5 (CH(*C*H_3)$_2$), 24.3 (CH(*C*H_3)$_2$) ppm.

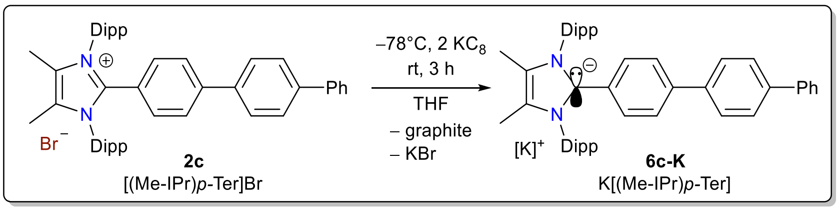

Synthesis of K[(Me-IPr)*p*-Ter)] (6c-K). To a Young NMR tube containing **2c** (21.0 mg, 0.03 mmol) and KC_8 (10.0 mg, 0.07 mmol) was added 550 µL of THF-d_8. A deep blue solution of **6c-K** was the immediate result. **^{1}H NMR** (500 MHz, THF-d_8, 298 K): 7.28 (t, *J* = 7.6 Hz, 2H, *p*-C_6H_3), 7.15 (d, *J* = 7.6 Hz, 4H, *m*-C_6H_3), 6.89 (d, *J* = 8.0 Hz, 2H, *o*-C_6H_5), 6.79 (t, *J* = 8.0 Hz, 2H, *m*-C_6H_5), 6.50 (d, *J* = 8.8 Hz, 2H, C_6H_4), 6.26 (t, *J* = 8.0 Hz, 1H, *p*-C_6H_5), 5.81 (m, 2H, C_6H_4), 5.40 (d, *J* = 9.5 Hz, 2H, C_6H_4), 4.77 (d, *J* = 9.5 Hz, 2H, C_6H_4), 3.33 (sept, *J* = 6.8 Hz, 4H, C*H*(CH_3)$_2$), 1.58 (s, 6H, NCCH_3), 1.27 (d, *J* = 6.8 Hz, 12H, CH(CH_3)$_2$), 1.21 (d, *J* = 6.8 Hz, 12H, CH(CH_3)$_2$) ppm. **^{13}C{^{1}H} NMR** (126 MHz, THF-d_8, 298 K): 149.6 (N*C*N), 148.8 (*o*-C_6H_3), 140.8 (*ipso*-C_6H_4), 138.2 (*ipso*-C_6H_3), 128.9 (*p*-C_6H_3), 128.8 (*m*-C_6H_5), 126.8 (*ipso*/*p*-C_6H_4), 124.5 (C_6H_4), 124.2 (*m*-C_6H_3), 124.1 (*ipso*/*p*-C_6H_4), 119.2 (*o*-C_6H_5), 119.0 (*ipso*-C_6H_5), 118.7 (C_6H_4), 116.9 (*p*-C_6H_5), 114.3 (C_6H_4), 112.5 (C_6H_4), 95.1 (*ipso*/*p*-C_6H_4), 29.2 (*C*H(CH_3)$_2$), 25.9 (N*C*CH_3), 25.5 (CH(*C*H_3)$_2$), 23.2 (CH(*C*H_3)$_2$), 10.2 (NC*C*H_3) ppm.

Synthesis of K[(SIPr)*m*-Ter] (7a-K)

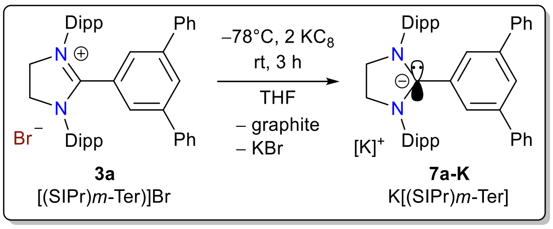

To a Young NMR tube containing **3a** (20.0 mg, 0.03 mmol) and KC_8 (10.0 mg, 0.07 mmol) was added 550µL THF-d_8. This led to the immediate formation of a deep blue solution of **7a-K**. **^{1}H NMR** (500 MHz, THF-d_8, 298 K): 7.09 (m, 4H, *m*-C_6H_3), 7.03 (m, 2H, *p*-C_6H_3), 6.96 (m, 8H, C_6H_5), 6.92 (m,

2H, *p*-C_6H_5), 4.52 (d, *J* = 1.84 Hz, 2H, *o*-$C_6H_3Ph_2$), 4.08 (m, 1H, *p*-$C_6H_3Ph_2$), 4.06 (m, 4H, C*H*$(CH_3)_2$), 3.63 (s, 4H, NCH_2), 1.37 (d, *J* = 6.8 Hz, 12H, CH$(CH_3)_2$), 1.24 (d, *J* = 6.8 Hz, 12H, CH$(CH_3)_2$) ppm. **^{13}C{^{1}H} NMR** (126 MHz, THF-d_8, 298 K): 147.7 (*o*-C_6H_3), 145.9 (NCN), 145.6 (*ipso*-C_6H_3), 140.5 (*ipso*-C_6H_5), 127.8 (C_6H_5), 125.4 (*p*-C_6H_5), 125.1 (*p*-C_6H_3), 124.1 (*m*-C_6H_3), 121.7 (*m*-$C_6H_3Ph_2$), 109.2 (*ipso*-$C_6H_3Ph_2$), 100.2 (*o*-$C_6H_3Ph_2$), 80.1 (*p*-$C_6H_3Ph_2$), 55.1 (NCH_2), 28.4 ($CH(CH_3)_2$), 26.0 (CH$(CH_3)_2$), 24.2 (CH$(CH_3)_2$) ppm.

Under similar conditions, reactions of **3b** and **3c** with KC_8 (> 2 equivalents) afford only the radicals **5b** and **5c**, respectively. Thus, **5b** and **5c** do not react further with an excess of KC_8 to form the corresponding anions.

Reactions of 4b and 5b with TEMPO

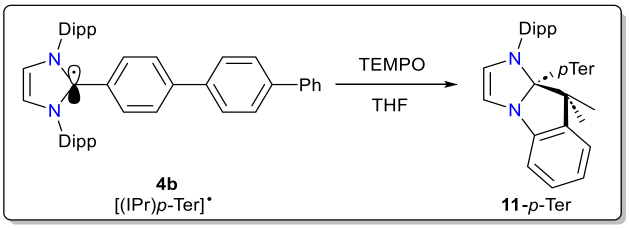

To a Schlenk tube containing **4b** (200 mg, 0.32 mmol) and TEMPO (53 mg, 0.36 mmol) was added 5 mL THF. The green solution was stirred overnight, which resulted in a light brown solution. Removal of the volatiles yielded compound **11**-*p*-Ter along with (likely) TEMPOH (total yield: 241 mg). Suitable crystals for sc-XRD analysis were obtained by storing a saturated benzene solution of **11**-*p*-Ter for 5 days. **ESI** (m/z) observed (calculated) [Fragment]:575.323 (575.343) [**11**-*p*-Ter+H]$^+$. **^{1}H NMR** (500 MHz, C_6D_6, 298 K): 7.44 (d, *J* = 7.8 Hz, 3H, *p*-Ter), 7.37 (d, *J* = 7.8 Hz, 4H, *p*-Ter), 7.25 (d, *J* = 7.8 Hz, 2H, *p*-Ter), 7.20 (m, 2H, 4H: C_6H_3, 4H: *p*-Ter), 7.03 (m, 2H, C_6H_3), 6.93 (d, *J* = 6.9 Hz, 1H, C_6H_3), 6.73 (d, *J* = 6.9 Hz, 1H, C_6H_3), 6.03 (s, 1H, NC*H*), 5.63 (s, 1H, NC*H*), 4.29 (sept, *J* = 6.9 Hz, 1H, C*H*$(CH_3)_2$), 3.54 (sept (overlaps with THF's signal), 1H, C*H*$(CH_3)_2$), 3.42 (sept, *J* = 6.9 Hz, 1H, C*H*$(CH_3)_2$), 2.04 (s, 3H, Dipp: C$(CH_3)_2$), 1.52 (d, *J* = 6.9 Hz, 3H, CH$(CH_3)_2$), 1.41 (m, 9H, C$(CH_3)_2$ (3H), CH_2 (TEMPO, 6H)), 1.37 (d, *J* = 6.9 Hz, 3H, CH$(CH_3)_2$), 1.32 (d, *J* = 6.9 Hz, 3H, CH$(CH_3)_2$), 1.24 (d, *J* = 6.6 Hz, 3H, CH$(CH_3)_2$), 1.16 (s, 12H, TEMPO CH_3), 0.36 (d, *J* = 6.9 Hz, 3H, CH$(CH_3)_2$), 0.21 (s, 3H, Dipp: C$(CH_3)_2$) ppm. **^{13}C{^{1}H} NMR** (126 MHz, C_6D_6, 298 K): 152.6 (C_6H_3), 149.9 (C_6H_3), 144.8 (C_6H_3), 143.1 (NCN), 141.3 (*p*-Ter), 140.9 (*p*-Ter), 140.3 (*p*-Ter), 139.6 (*p*-Ter), 138.6 (*p*-Ter), 136.8 (C_6H_3), 136.2 (C_6H_3), 129.1 (*p*-Ter), 127.7 (*p*-Ter), 127.5 (*p*-Ter), 127.4 (*p*-Ter), 126.3 (NCH), 125.7 (C_6H_3), 125.6 (C_6H_3), 125.0 (C_6H_3), 123.2 (CH$(CH_3)_2$) or TEMPO)), 120.7 (C_6H_3), 119.4 (NCH), 101.3 (NC$C(CH_3)_2$), 58.6, 55.5, 39.9 (TEMPO), 31.1 (C$(CH_3)_2$), 29.0, 28.6, 27.9 ($CH(CH_3)_2$), 26.4, 26.2, 24.6, 23.9, 23.6, 23.0, 22.1 (CH$(CH_3)_2$) and TEMPO), 17.6 (CH$(CH_3)_2$), 1.0 (C$(CH_3)_2$) ppm.

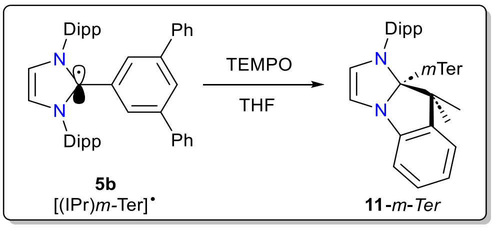

Compound **11**-*m*-Ter was synthesized using **5b** (202 mg, 0.32 mmol) and TEMPO (56 mg, 0.36 mmol) in a similar reaction protocol as described for **11**-*p*-Ter (total yield: 256 mg). **ESI** (m/z): observed (calculated) [Fragment]:575.320 (575.343) [**11**-*m*-Ter+H]$^+$. **^{1}H NMR** (500 MHz, C_6D_6, 298 K): 7.82 (s, 1H, $C_6H_3Ph_2$), 7.47 (s, 1H, $C_6H_3Ph_2$), 7.35 (m, 4H, *m*-Ter and C_6H_3), 7.04 (m, 12H, *m*-Ter and C_6H_3), 6.65 (d, J = 6.9 Hz, C_6H_3), 6.02 (s, 1H, NC*H*), 5.62 (s, 1H, NC*H*), 4.27 (sept, J = 6.9 Hz, 1H, C*H*(CH_3)$_2$), 3.54 (sept (overlaps with THF's signal), 1H, C*H*(CH_3)$_2$), 3.42 (sept, J = 6.9 Hz, 1H, C*H*(CH_3)$_2$), 1.87 (s, 3H, Dipp: C(CH_3)$_2$), 1.35 (m, 26H, 22H: CH(CH_3)$_2$ and TEMPO), 1.20 (t, J = Hz, 9H,), 1.15 (s, 12H,), 0.31 (d, J = Hz, 3H,), 0.21 (s, 3H, Dipp: C(CH_3)$_2$) ppm. **^{13}C{^{1}H} NMR** (126 MHz, C_6D_6, 298 K): 153.0 (*C*$_6$H$_3$ of Dipp), 150.3 (*C*$_6$H$_3$ of Dipp), 144.6 (*C*$_6$H$_3$ of Dipp), 143.0 (N*C*N), 142.1 (*m*-Ter), 142.0 (*m*-Ter), 141.8 (*m*-Ter), 141.2 (*m*-Ter), 139.6 (*m*-Ter), 138.5 (*m*-Ter), 136.5 (*C*$_6$H$_3$ of Dipp), 129.7 (*m*-Ter), 128.9 (*m*-Ter), 128.8 (*m*-Ter), 127.6 (*m*-Ter), 127.4 (*m*-Ter), 127.1 (*C*$_6$H$_3$ of *m*-Ter), 126.2 (N*C*H), 125.6 (*C*$_6$H$_3$ of Dipp), 124.9 (*C*$_6$H$_3$), 124.3 (*C*$_6$H$_3$Ph$_2$), 123.4 (*m*-Ter), 120.7 (*C*$_6$H$_3$), 119.4 (N*C*H), 101.3 (NC*C*(CH$_3$)$_2$), 58.6 (TEMPO), 55.4 (TEMPO), 39.9 (CH(*C*H$_3$)$_2$) and TEMPO), 30.9 (C(*C*H$_3$)$_2$), 28.1 (CH(*C*H$_3$)$_2$) and TEMPO), 26.2 (CH(C*H*$_3$)$_2$), 25.8, 24.4, 23.0, 21.9 (CH(*C*H$_3$)$_2$) and TEMPO), 17.6 (CH(C*H*$_3$)$_2$), 1.0 (C(*C*H$_3$)$_2$) ppm.

NMR Monitoring of the Reactions of 4b with TEMPO

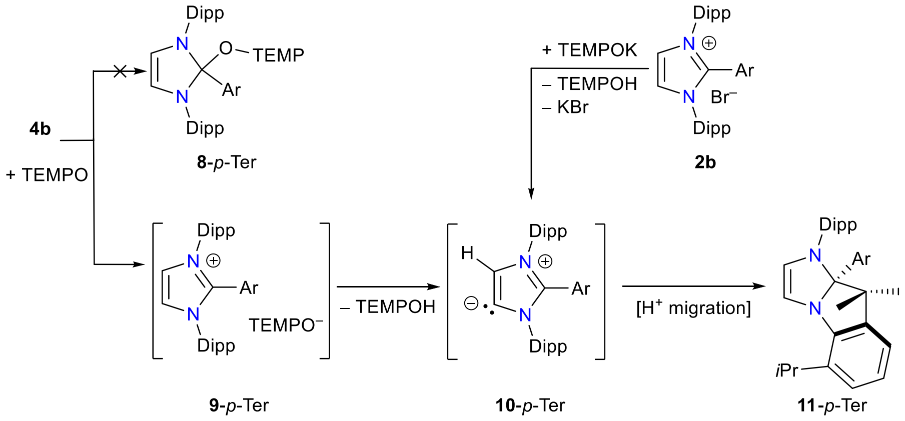

To obtain further insight into the formation of **11**-*p*-Ter, instead of the expected radical coupling products **8**-*p*-Ter, we monitored the progress of the reaction of **4b** with TEMPO in a Young NMR tube in C_6D_6 by ^{1}H NMR spectroscopy (Figures S1 and S2). Both radicals **4b** and TEMPO are NMR silent (Figure S1).

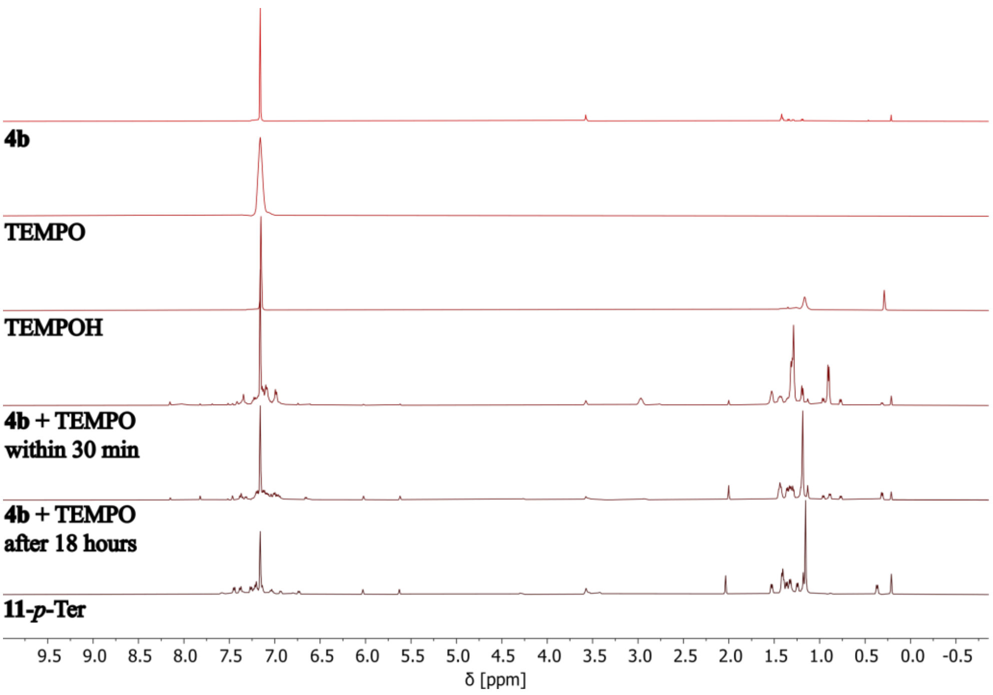

Figure S1. Stack of ^{1}H NMR (500 MHz, C_6D_6, 298 K) spectra of 4b, TEMPO, TEMPOH, reacting **4b** with TEMPO and the isolated product **11**-*p*-Ter.

On addition of TEMPO to a C_6D_6 sample of **4b** leads to the formation of several diamagnetic species, including iMIC (**10**-*p*-Ter, major),[6] **11**-*p*-Ter, and an unknown species (Figure S2).

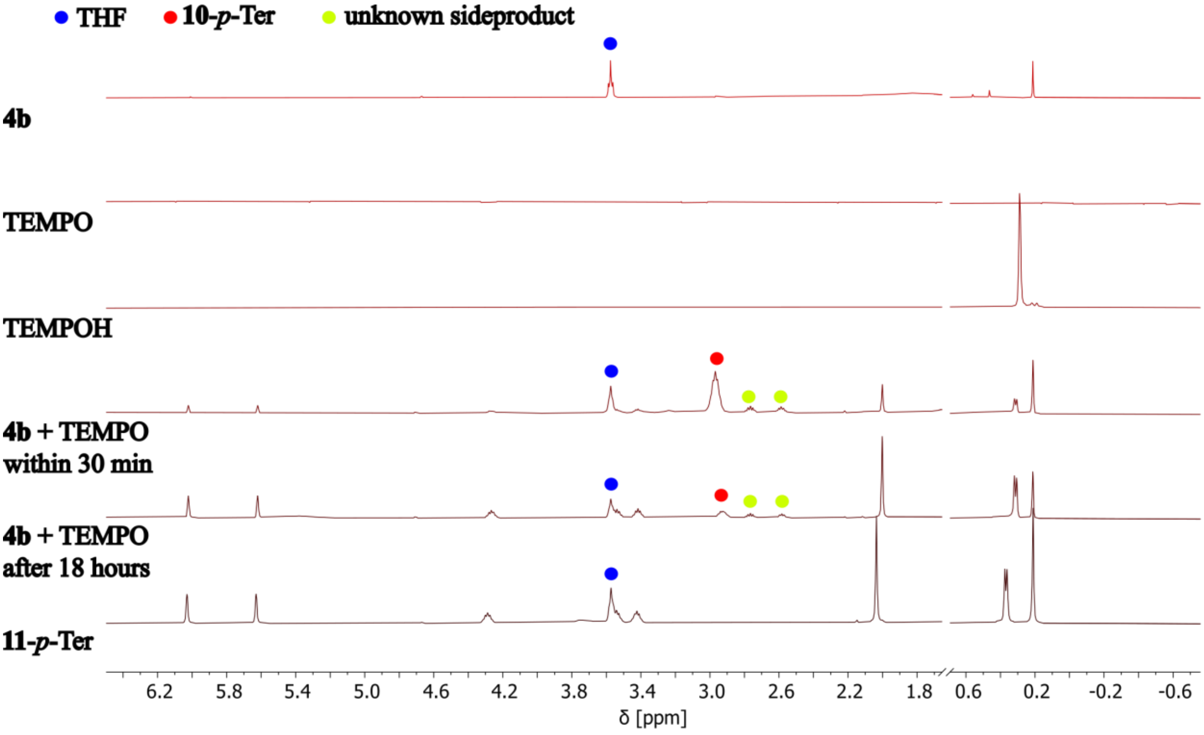

Figure S2. Zoomed in stack of ^{1}H NMR (500 MHz, C_6D_6, 298 K) spectra of 4b, TEMPO, TEMPOH, reacting **4b** with TEMPO and the isolated product **11**-*p*-Ter.

The formation of molecular hydrogen can be excluded as no signal for H_2 was seen in the ^{1}H NMR spectrum. After one day (at rt), the amount of iMIC (**10**-*p*-Ter) decreases, while **11**-*p*-Ter increases. However, the signals due to the unknown species remained unchanged.

To probe the involvement of the iMIC (**10**-*p*-Ter), we performed reaction **2b** (16.0 mg, 0.02 mmol) with KHMDS (4.7 mg, 0.02 mmol) in C_6D_6. This led to the clean formation of iMIC (**10**-*p*-Ter) as a single species along with $HN(SiMe_3)_2$ (Figure S3). The ^{1}H NMR signals of this sample compare well with the major species formed after mixing **4b** with TEMPO (Figure S2). Interestingly, the C_6D_6 solution of iMIC (**10**-*p*-Ter) is stable at 70 °C as no change in the ^{1}H NMR spectrum was observed after 1h. This suggests that TEMPO (or related species) plays an important role in the formation of **11**-*p*-Ter.

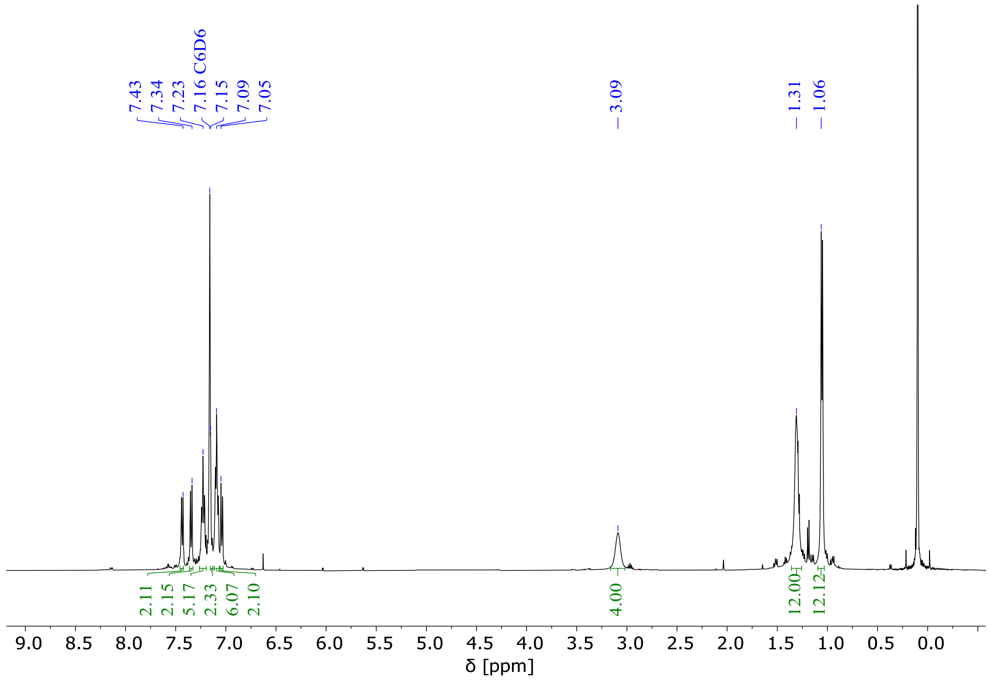

Figure S3. ^{1}H NMR (500 MHz, C_6D_6, 298 K) spectrum of reacting **2b** with KHMDS.

Therefore, we decided to deprotonate **2b** with TEMPOK in generating iMIC (**10**-*p*-Ter) and TEMPOH (see below). To an NMR tube containing **2b** (21.0 mg, 0.03 mmol) and TEMPOK (6.0 mg, 0.03 mmol) was added C_6D_6, which resulted in a deep green suspension immediately, like in the case with KHMDS (see above, Figure 3). The ^{1}H NMR spectrum measured showed the clean formation of iMIC (**10**-*p*-Ter) and a small amount of **11**-*p*-Ter. After 1h at room, the amount of iMIC (**10**-*p*-Ter) decreases while **11**-*p*-Ter increases (~ 3:1 ratio). Also, the solution turned yellowish. The 1H NMR spectrum measured after 18 h showed that iMIC (**10**-*p*-Ter) has almost completely converted into **11**-*p*-Ter.

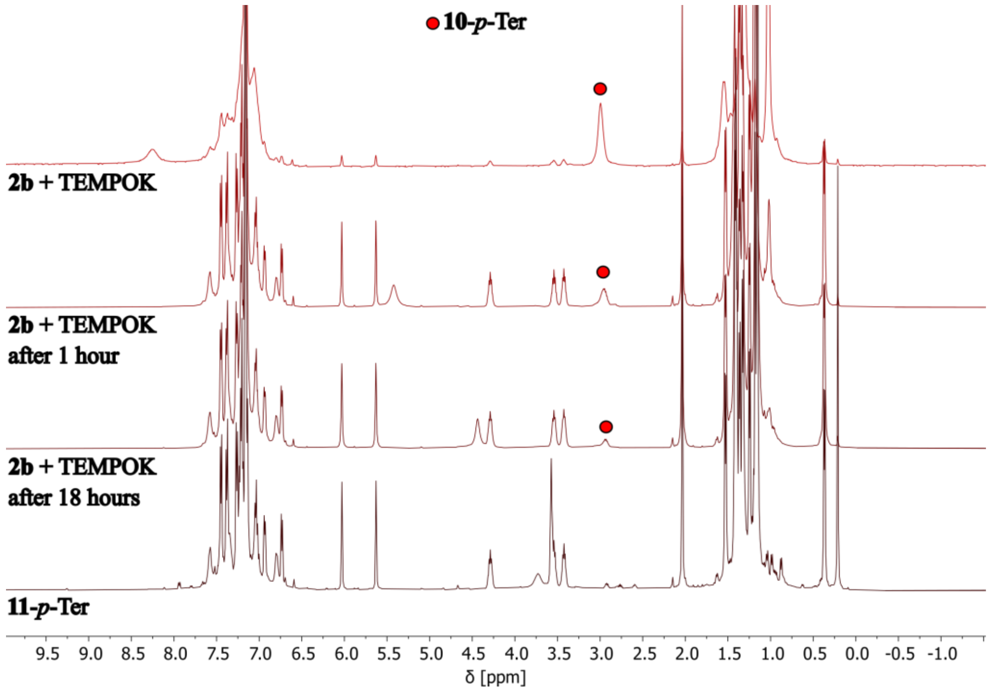

Figure S4. Stack of ^{1}H NMR (500 MHz, C_6D_6, 298 K) spectra of reacting **4b** with TEMPOK.

These results indicate the initial formation of iMIC (**10**-*p*-Ter), which in the presence of TEMPOH underwent C-H bond activation to ultimately yield **11**-*p*-Ter. The exact role of TEMPO (or TEMPOH) is currently not known, however, this transformation of **4b** into **11**-*p*-Ter can also be affected in the presence of a catalytic amount of TEMPO.

One-electron Oxidation Reactions of Radicals

Oxidation of 4b with AgOTf: Radicals **4**a–c and **5**a–c (10 mg) were oxidized using AgOTf (1 equivalent) in C_6D_6 (550 µL) in a Young NMR tube, respectively. In each case, the characteristic green (to green-yellow) color disappeared, yielding a brownish yellow suspension featuring characteristic NMR Signals for the respective cationic [**2**a–c]$^{+}$ and [**3**a–c]$^{+}$ parts.

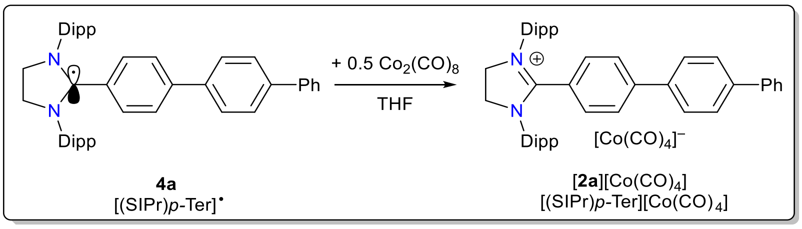

Reaction of 4a with $Co_2(CO)_8$: [(SIPr)*p*-Ter] (**4a**) (77 mg, 0.12 mmol) and $Co_2(CO)_8$ (43 mg, 0.06 mmol) were dissolved in 5 mL of THF and stirred overnight. The characteristic green color of **4a** changed to brown within seconds. All volatiles were removed to give [**2a**][$Co(CO)_4$] as a off-white crystalline solid in 99% (96 mg) yield. Single crystals suitable for x-ray diffraction analysis were obtained by storing a saturated benzene solution of [**2a**][$Co(CO)_4$] over 3 days. **MP**: 260 °C. **ESI** (m/z)

observed (calculated) [Fragment]: 619.399 (619.404) $[M–Co(CO)_4]^+$ and 170.906 (170.913) $[Co(CO)_4]^–$. **^{1}H NMR** (500 MHz, C_6D_6, 298 K): 7.53 (br. m, 13H, C_6H_3 and *p*-Ter), 7.36 (s, 3H, *p*-Ter), 7.26 (signal overlaps with C_6D_6's signal, 1H, *p*-Ter), 7.10 (br. S, 2H, *p*-Ter), 4.70 (br. s, NCH_2), 3.08 (br. s, $CH(CH_3)_2$), 1.44 (br. s, $CH(CH_3)_2$), 1.06 (br. s, $CH(CH_3)_2$) ppm. **^{13}C{^{1}H} NMR** (126 MHz, C_6D_6, 298 K): 145.7 (C_6H_3), 142.3 (*p*-Ter), 140.0 (*p*-Ter), 136.6 (*p*-Ter), 131.4 (*p*-Ter), 130.3 (C_6H_3), 129.0 (*p*-Ter), 128.4 (*p*-Ter), 127.9 (*p*-Ter), 127.5 (*p*-Ter), 127.1 (*p*-Ter), 126.8 (*p*-Ter), 125.8 (C_6H_3), 30.6 ($CH(CH_3)_2$), 29.7 ($CH(CH_3)_2$), 23.6 ($CH(CH_3)_2$) ppm.

Plots of NMR Spectra

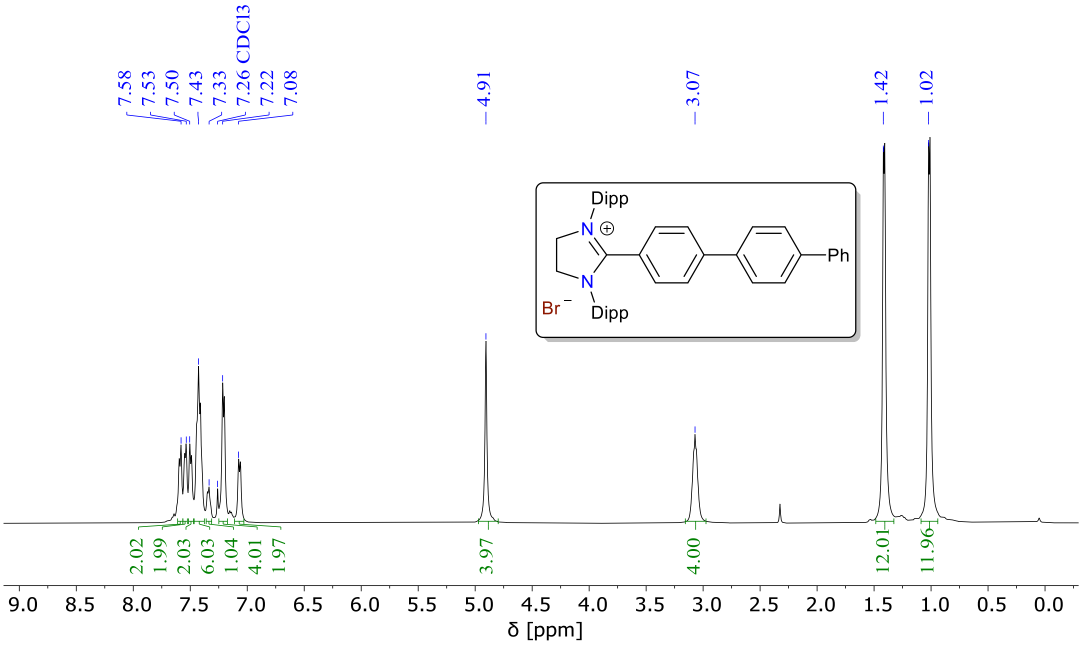

Figure S5. ^{1}H NMR (500 MHz, $CDCl_3$, 298 K) spectrum of **2a**.

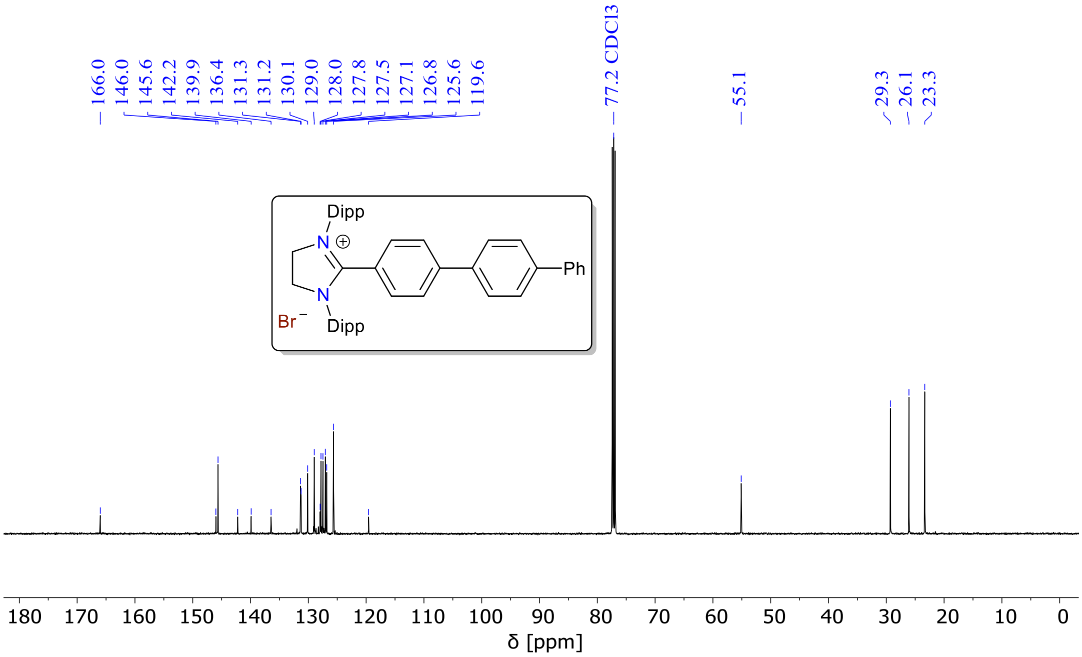

Figure S6. $^{13}C\{^{1}H\}$ NMR (125 MHz, $CDCl_3$, 298 K) spectrum of **2a**.

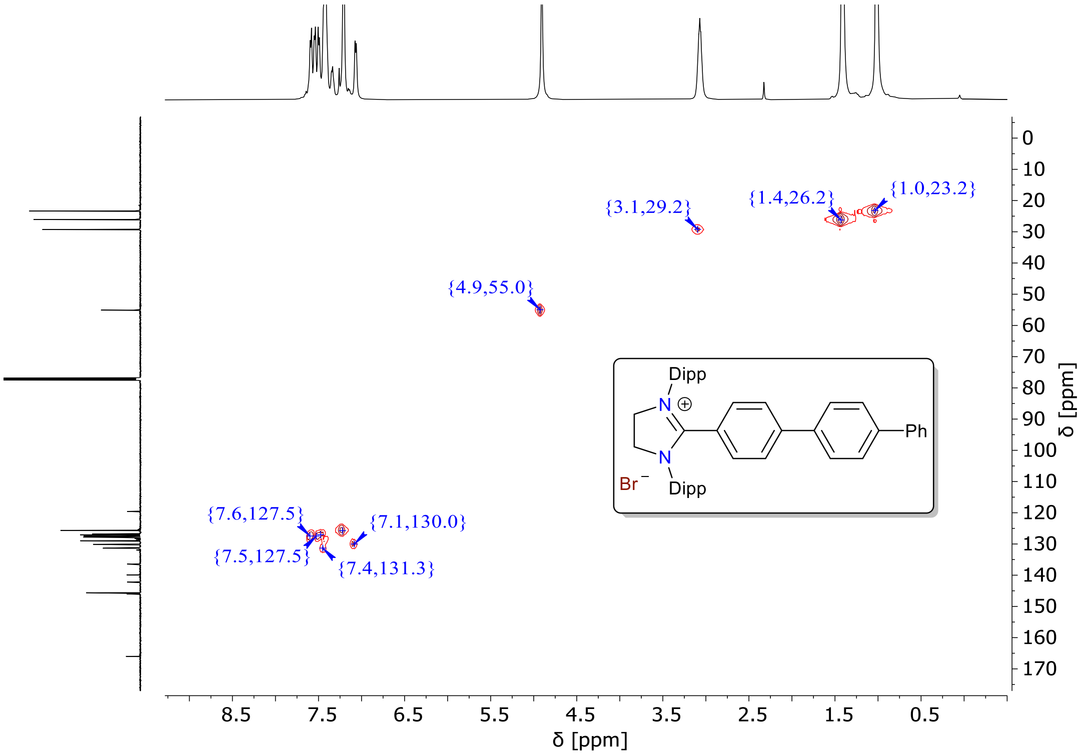

Figure S7. ^{1}H-$^{13}C\{^{1}H\}$ HMQC NMR (500/125 MHz, $CDCl_3$, 298 K) spectrum of **2a**.

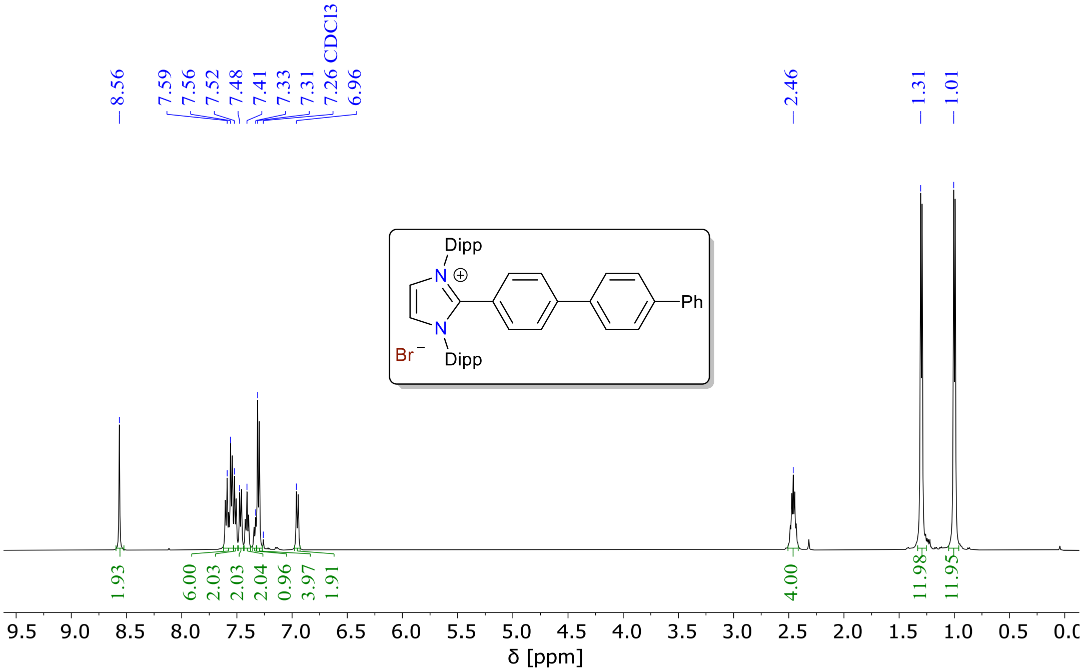

Figure S8. ^{1}H NMR (500 MHz, $CDCl_3$, 298 K) spectrum of **2b**.

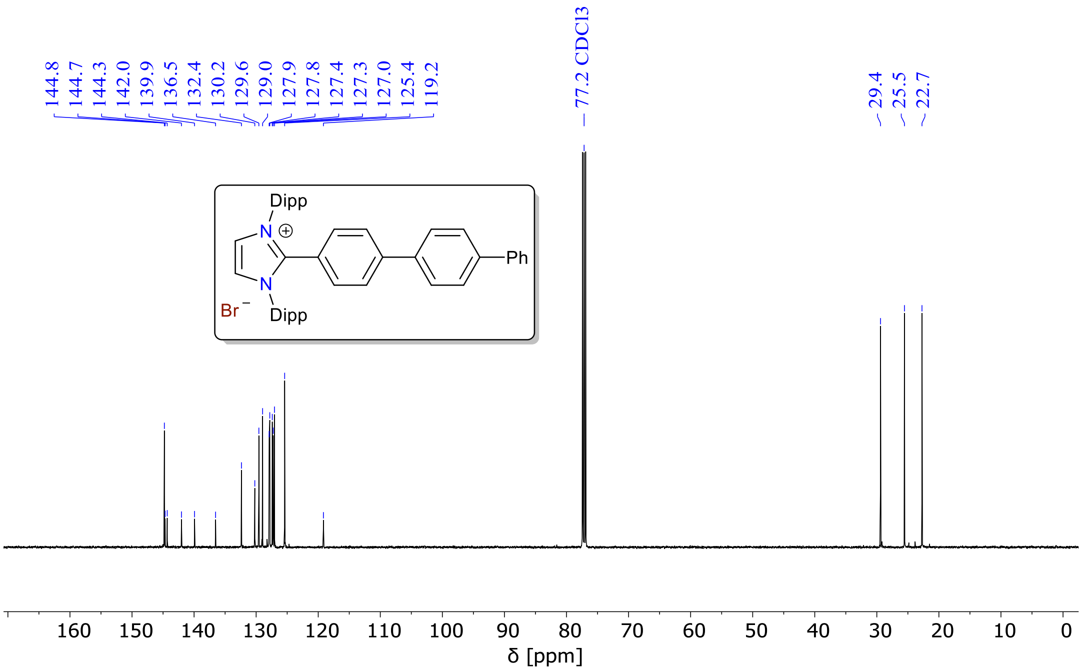

Figure S9. $^{13}C\{^{1}H\}$ NMR (125 MHz, $CDCl_3$, 298 K) spectrum of **2b**.

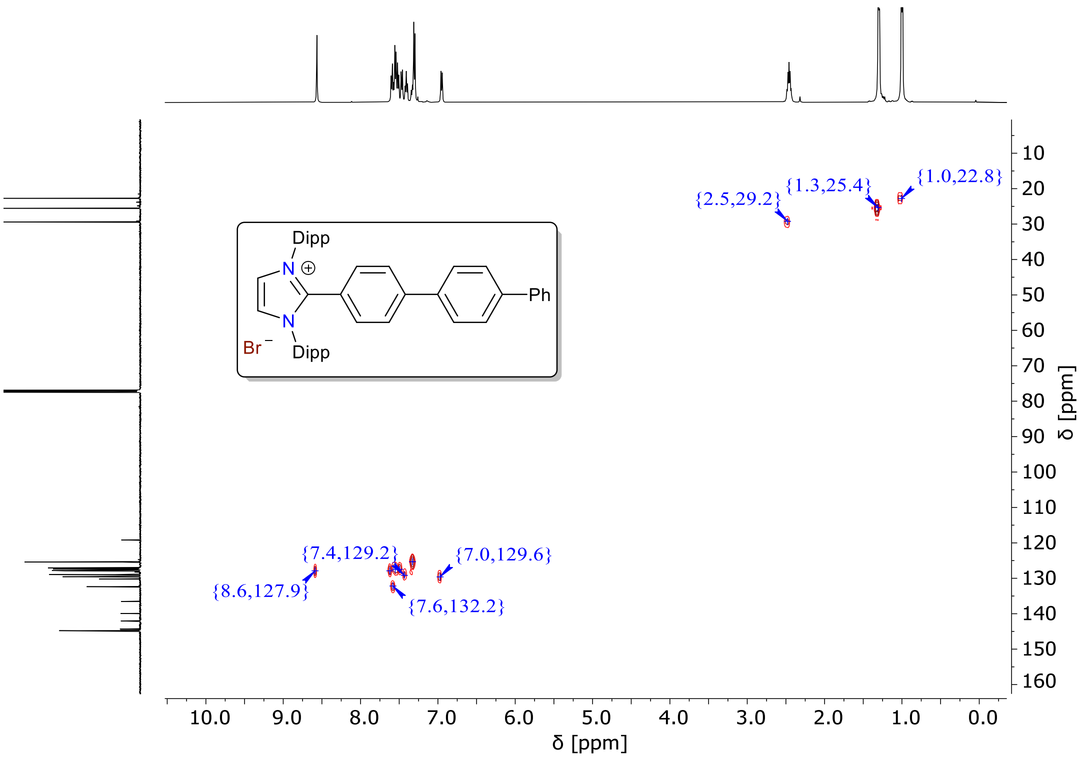

Figure S10. ^{1}H-$^{13}C\{^{1}H\}$ HMQC NMR (500/125 MHz, $CDCl_3$, 298 K) spectrum of **2b**.

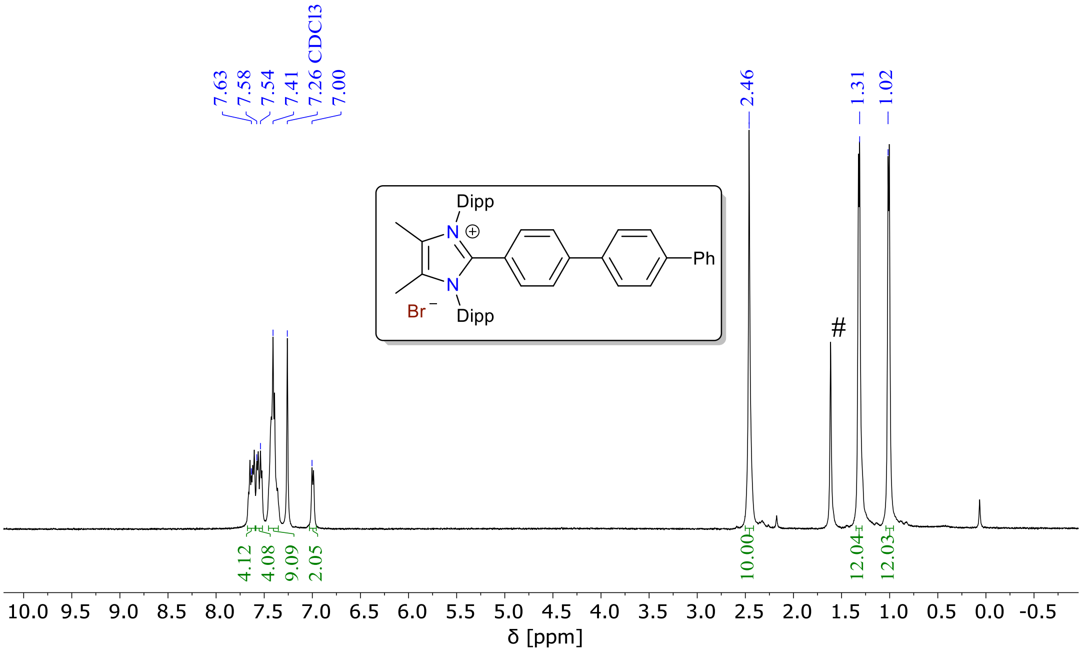

Figure S11. ^{1}H NMR (500 MHz, $CDCl_3$, 298 K) spectrum of **2c**. $^{\#}H_2O$ from $CDCl_3$.

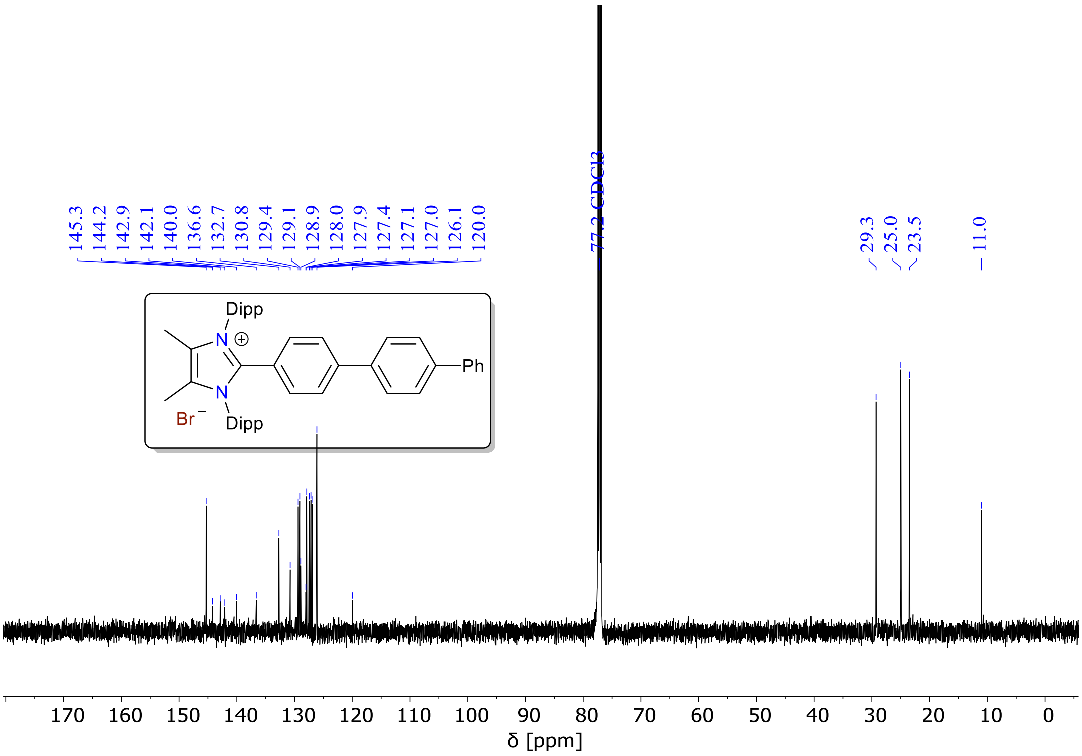

Figure S12. $^{13}C\{^{1}H\}$ NMR (125 MHz, $CDCl_3$, 298 K) spectrum of **2c**.

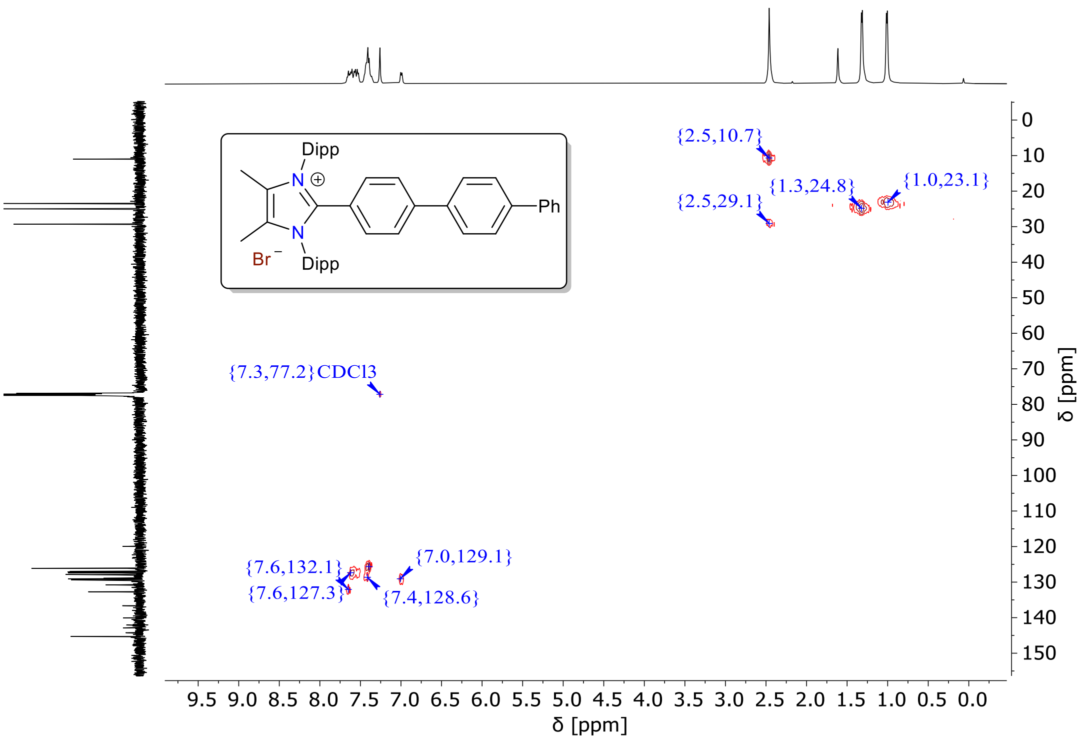

Figure S13. ^{1}H-^{13}C{^{1}H} HMQC NMR (500/125 MHz, CDCl$_3$, 298 K) spectrum of **2c**.

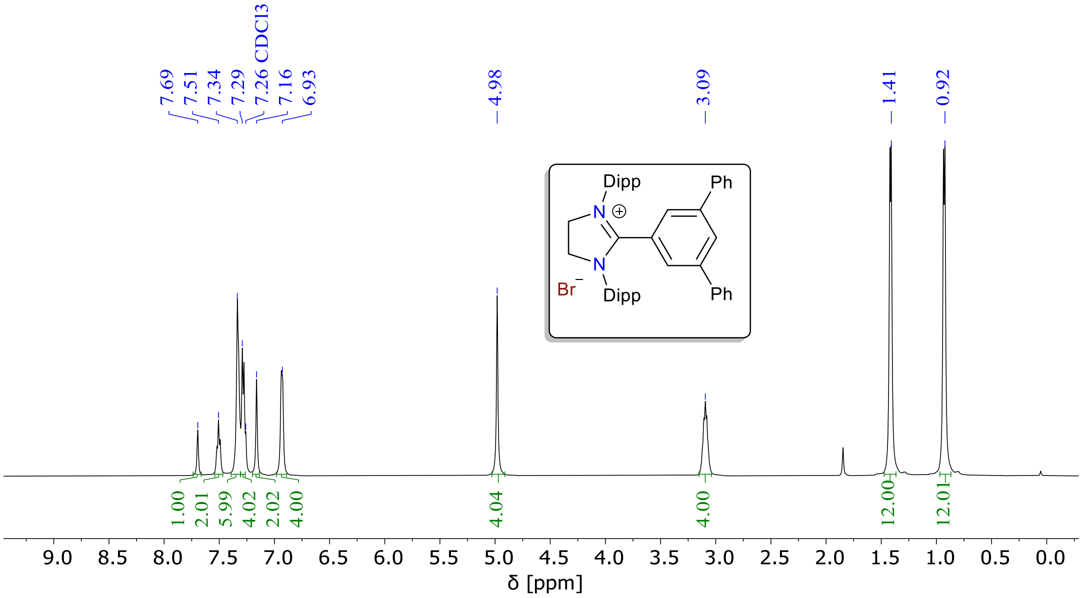

Figure S14. ^{1}H NMR (500 MHz, CDCl$_3$, 298 K) spectrum of **3a**.

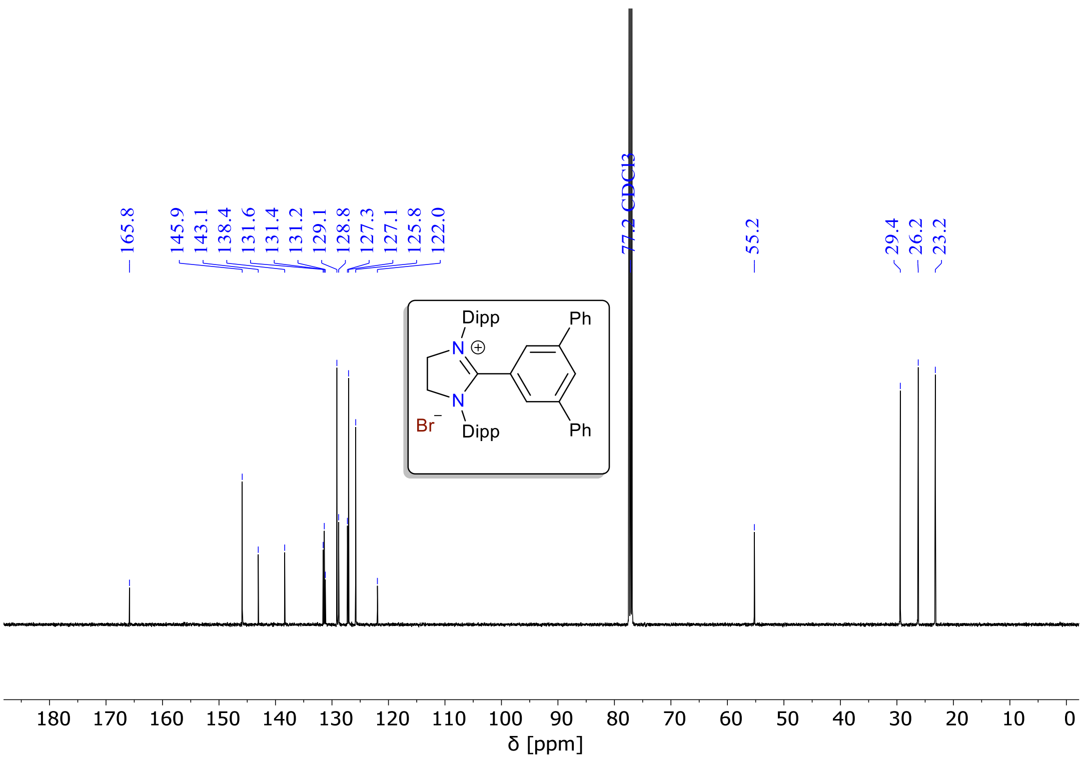

Figure S15. $^{13}C\{^{1}H\}$ NMR (125 MHz, $CDCl_3$, 298 K) spectrum of **3a**.

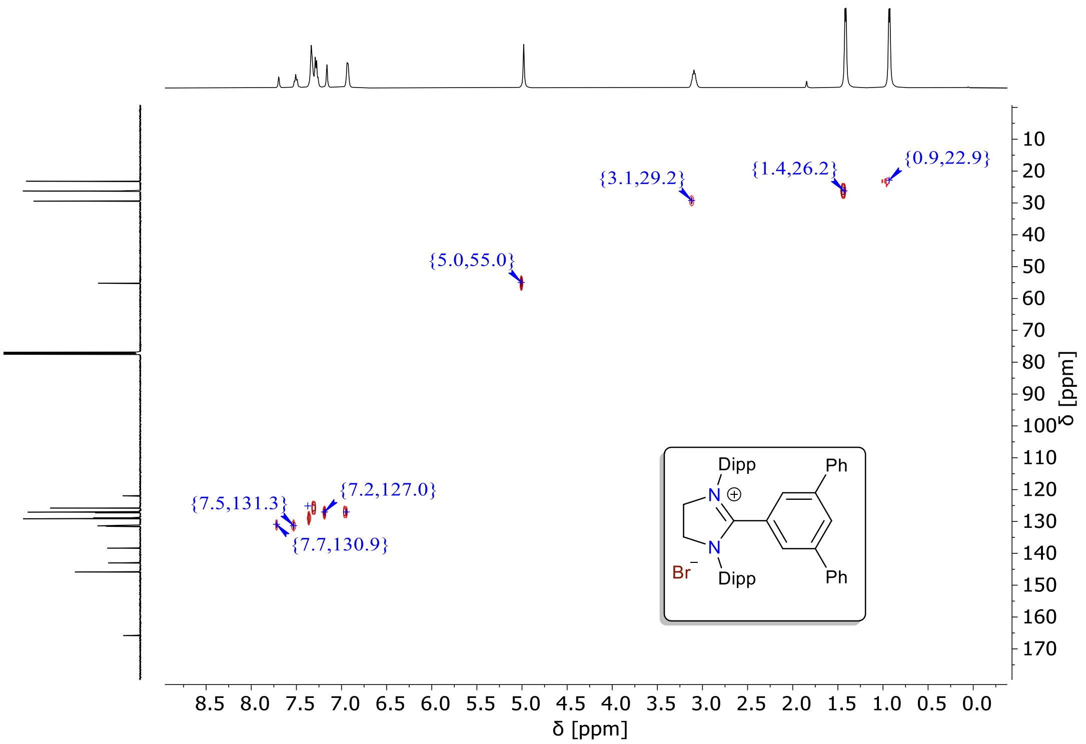

Figure S16. ^{1}H-$^{13}C\{^{1}H\}$ HMQC NMR (500/125 MHz, $CDCl_3$, 298 K) spectrum of **3a**.

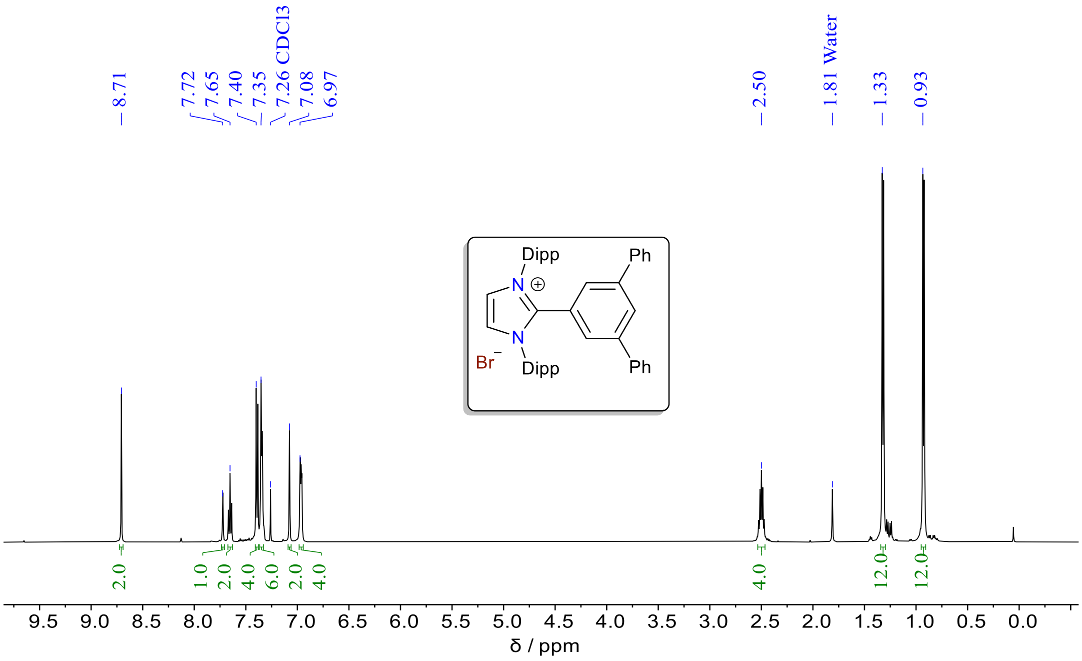

Figure S17. ^{1}H NMR (500 MHz, $CDCl_3$, 298 K) spectrum of **3b**.

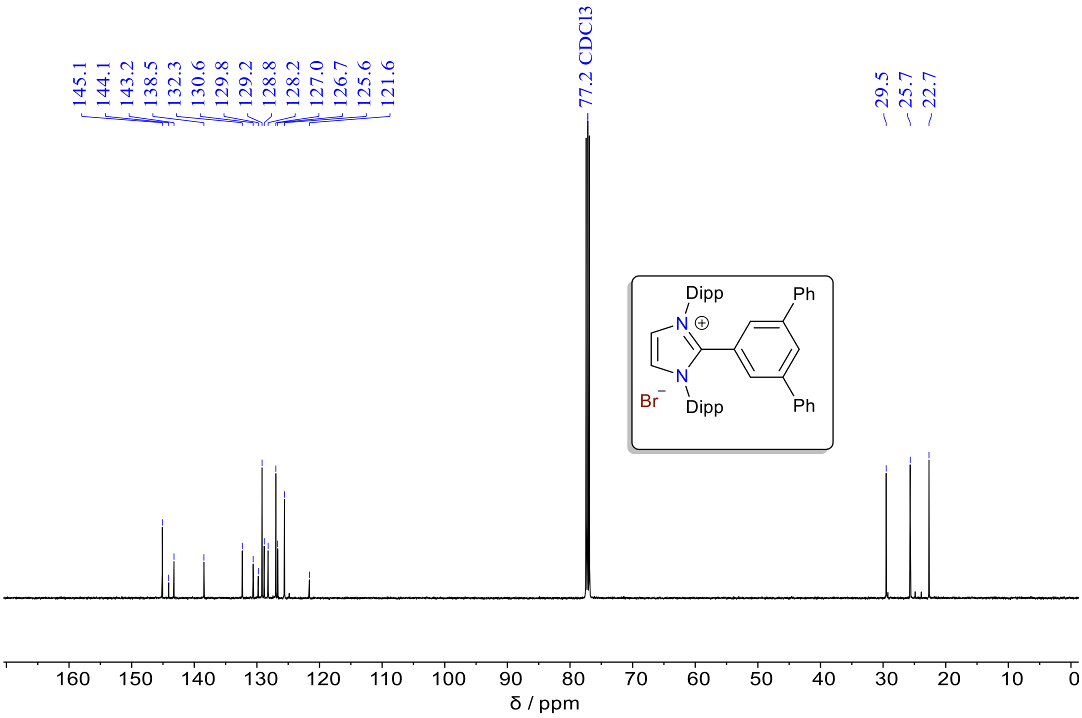

Figure S18. $^{13}C\{^{1}H\}$ NMR (125 MHz, $CDCl_3$, 298 K) spectrum of **3b**.

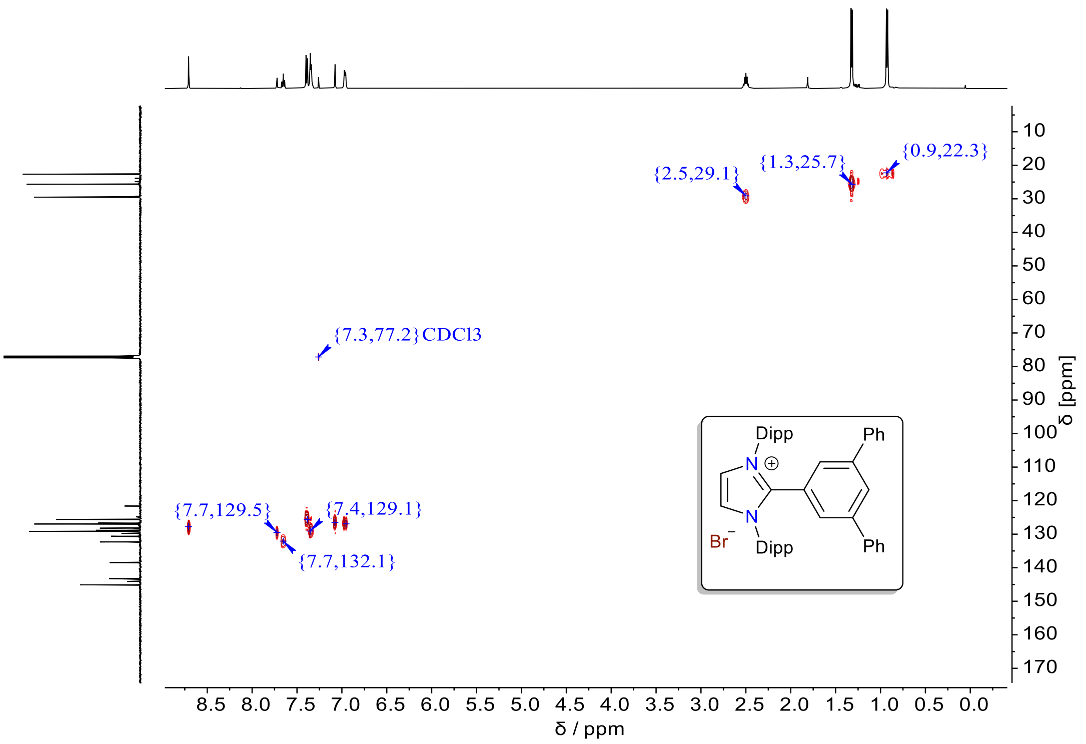

Figure S19. ^{1}H-$^{13}C\{^{1}H\}$ HMQC NMR (500/125 MHz, $CDCl_3$, 298 K) spectrum of **3b**.

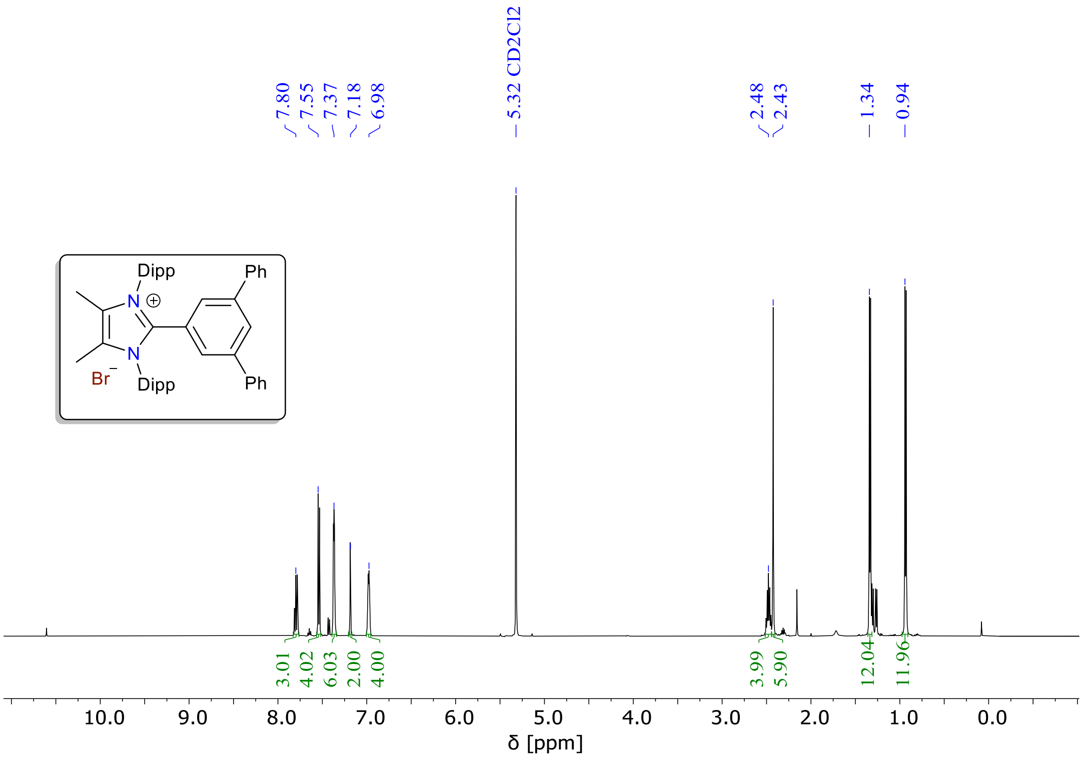

Figure S20. ^{1}H NMR (500 MHz, $CDCl_3$, 298 K) spectrum of **3c**.

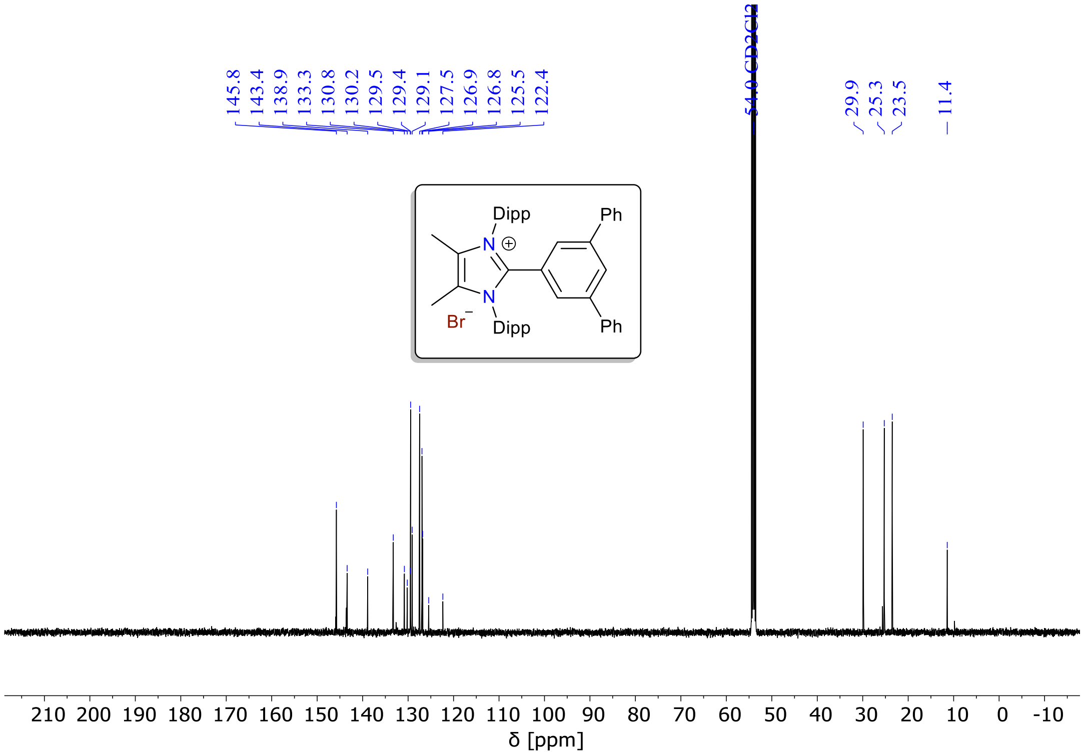

Figure S21. $^{13}C\{^{1}H\}$ NMR (125 MHz, $CDCl_3$, 298 K) spectrum of **3c**.

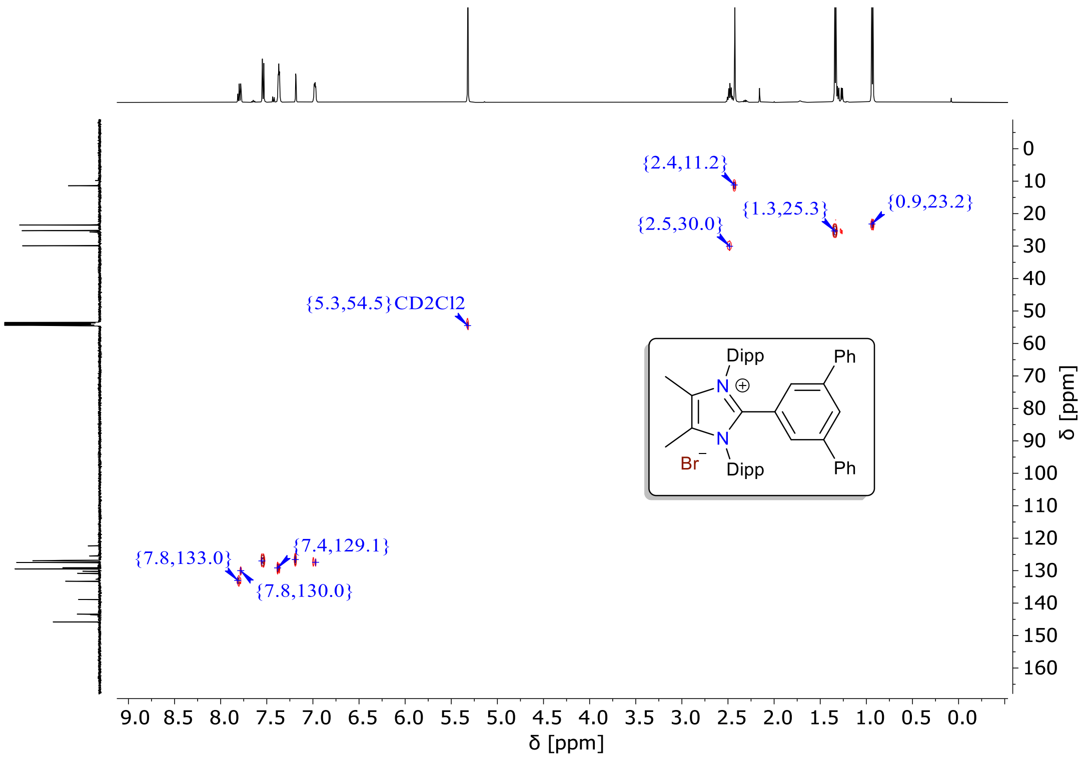

Figure S22. ^{1}H-$^{13}C\{^{1}H\}$ HMQC NMR (500/125 MHz, $CDCl_3$, 298 K) spectrum of **3c**.

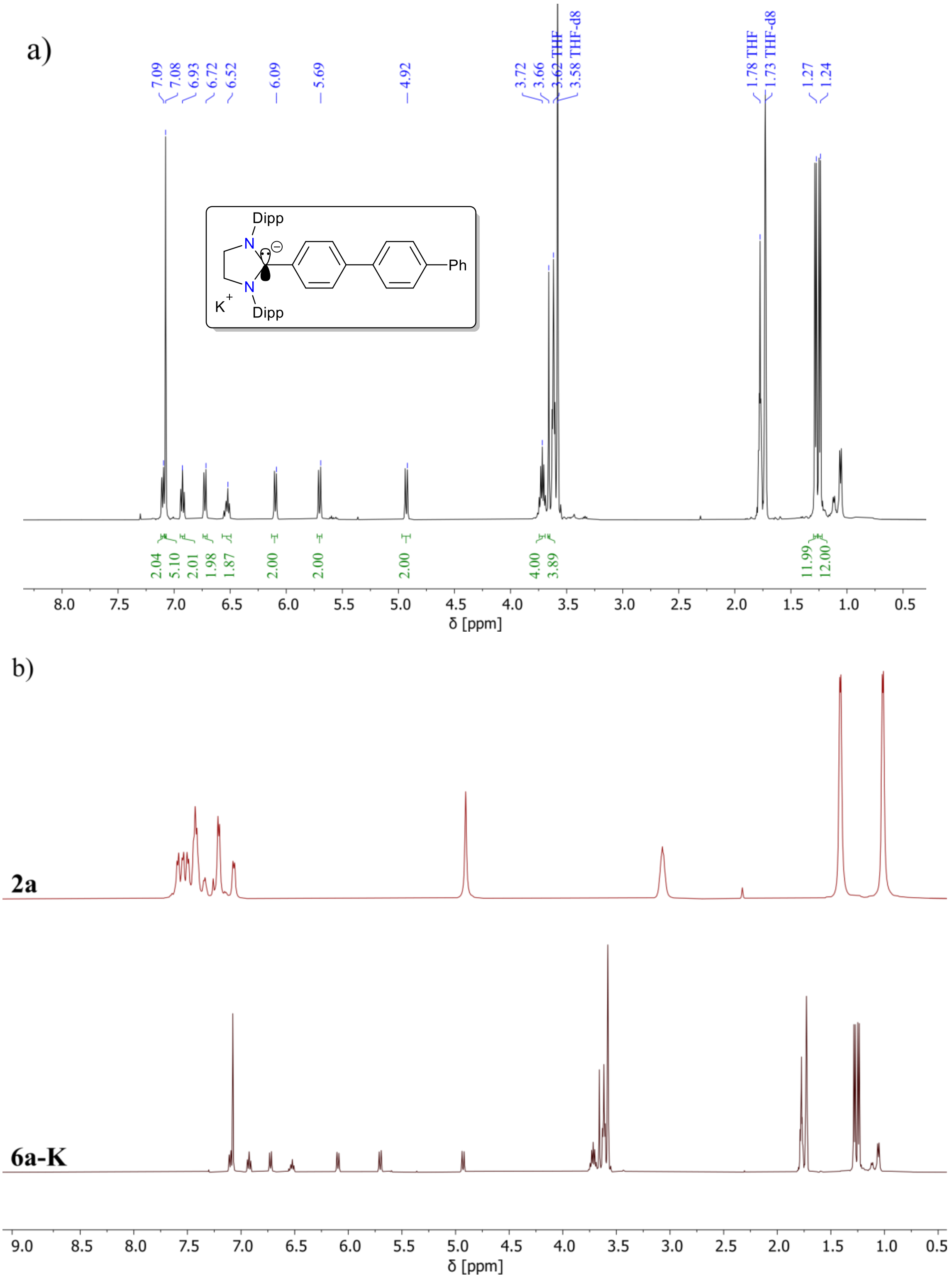

Figure S23. a) ^{1}H NMR (500 MHz, THF-d_8, 298 K) spectrum of **6a-K**. b) Stacked ^{1}H NMR spectra of **2a** (in $CDCl_3$) and **6a-K** (in THF-d_8).

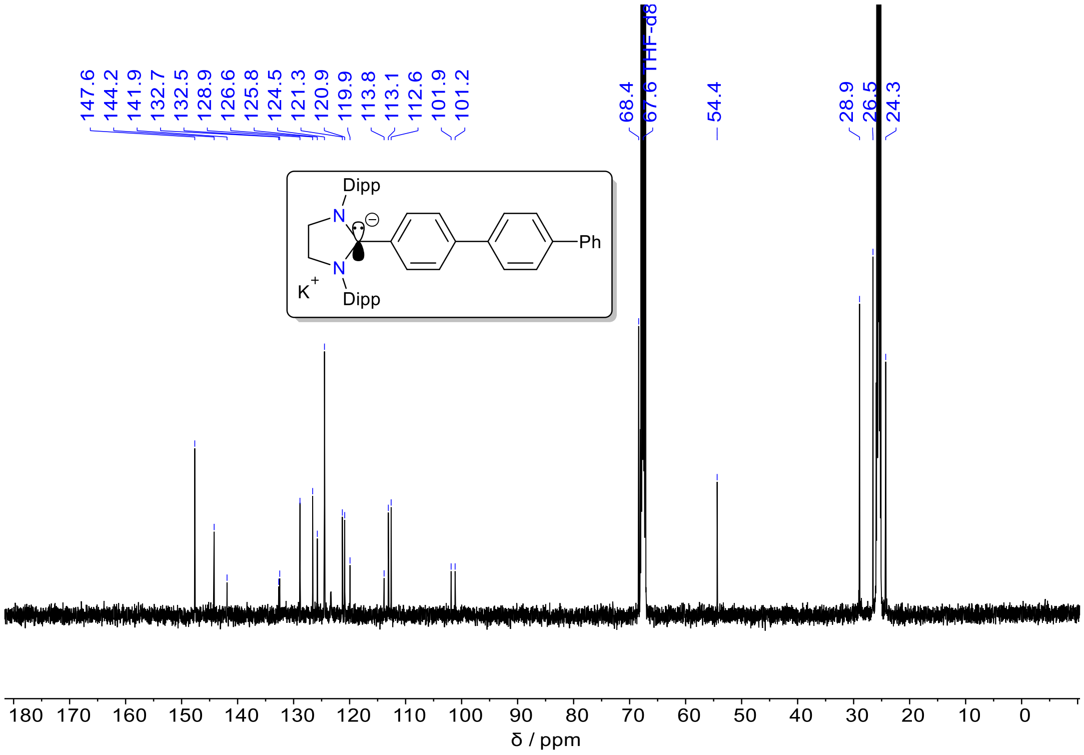

Figure S24. $^{13}C\{^1H\}$ NMR (125 MHz, THF-d_8, 298 K) spectrum of **6a-K**.

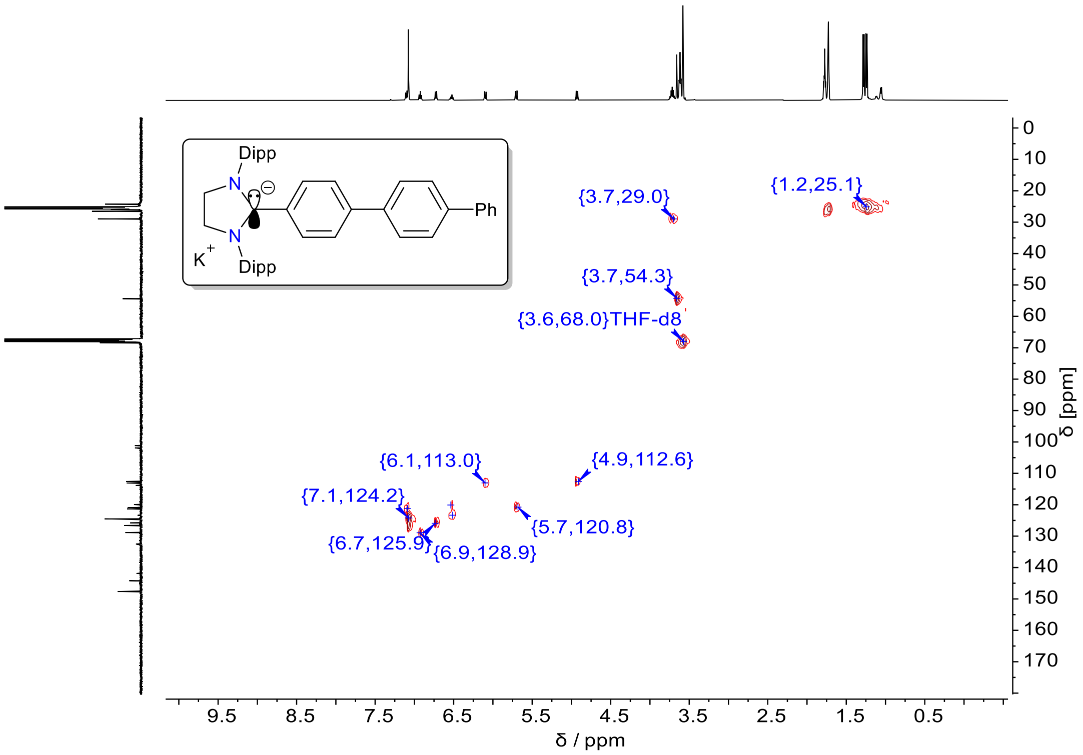

Figure S25. ^1H-$^{13}C\{^1H\}$ HMQC NMR (500/125 MHz, THF-d_8, 298 K) spectrum of **6a-K**.

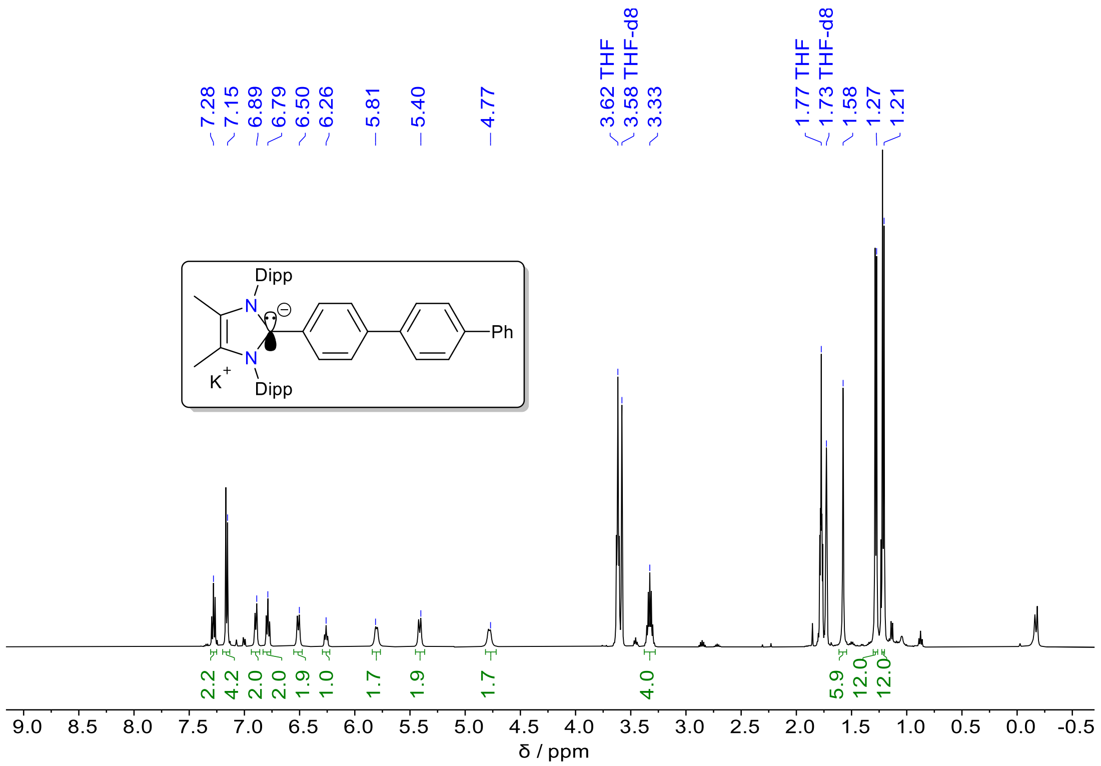

b)

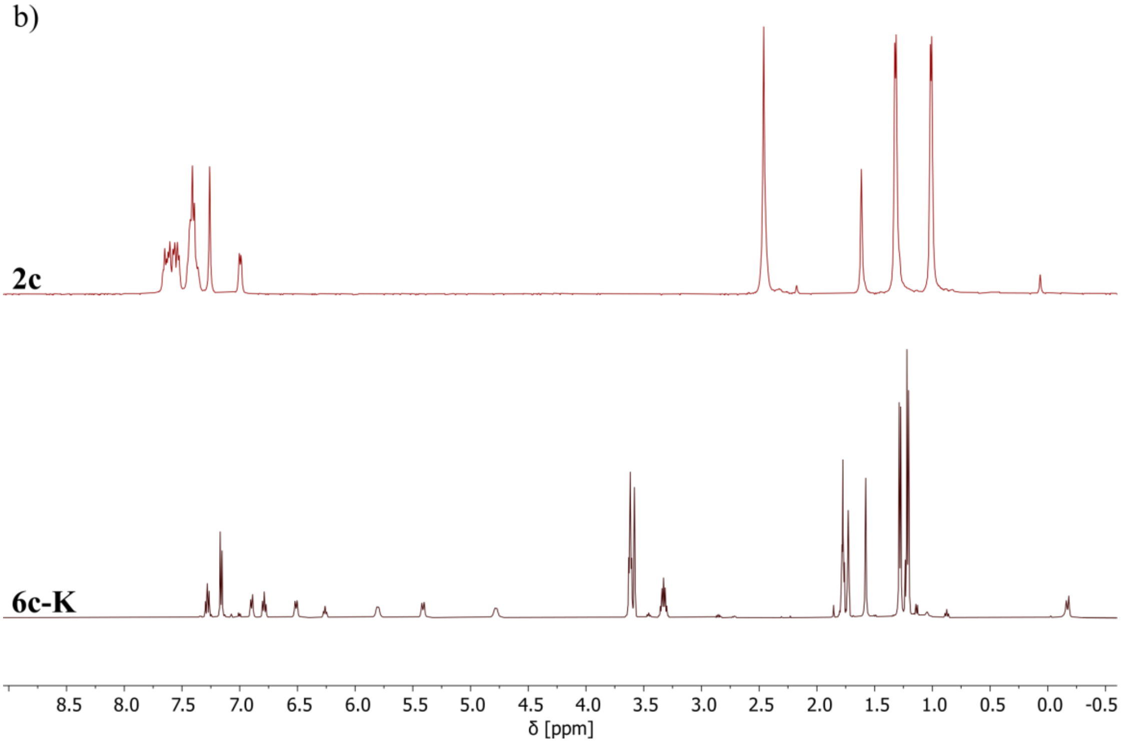

Figure S26. a) ^{1}H NMR (500 MHz, THF-d_8, 298 K) spectrum of **6c-K**. b) Stacked ^{1}H NMR spectra of **2c** (in $CDCl_3$) and **6c-K** (in THF-d_8).

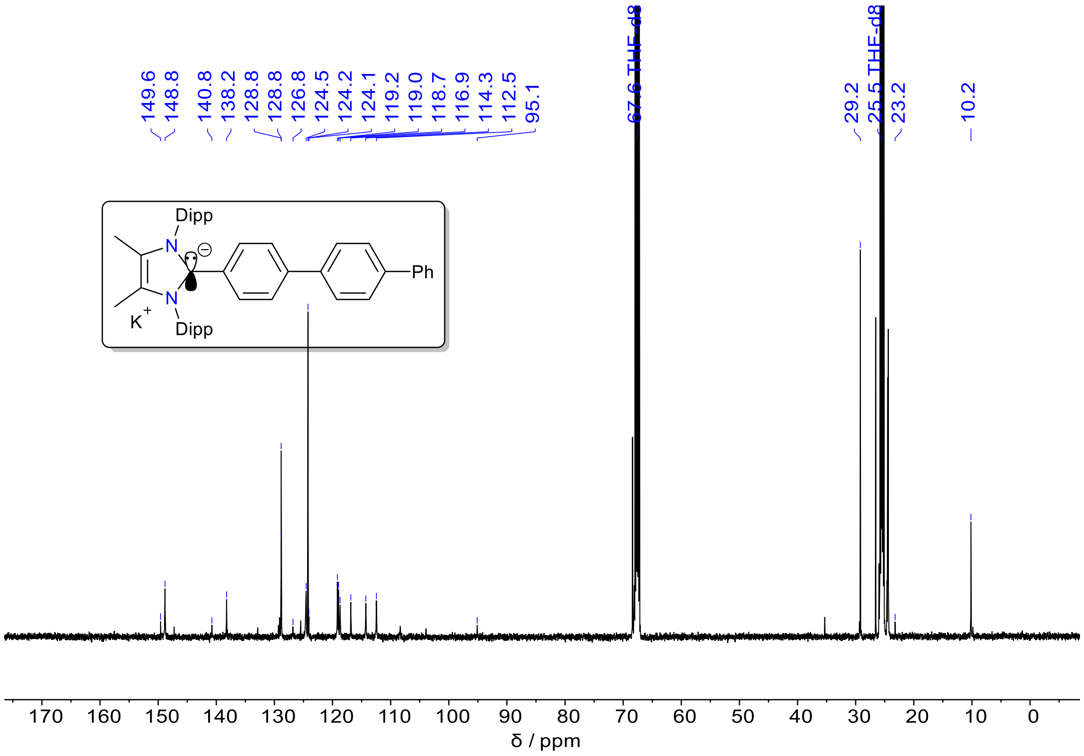

Figure S27. $^{13}C\{^{1}H\}$ NMR (125 MHz, THF-d_8, 298 K) spectrum of **6c-K**.

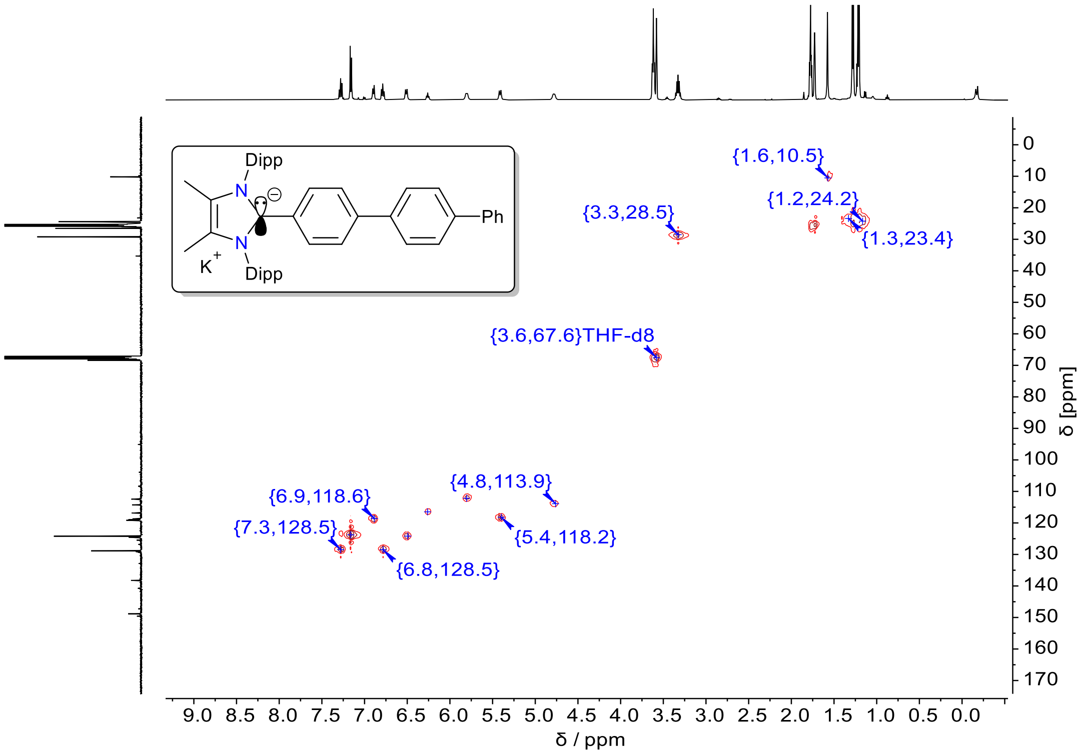

Figure S28. ^{1}H-$^{13}C\{^{1}H\}$ HMQC NMR (500/125 MHz, THF-d_8, 298 K) spectrum of **6c-K**.

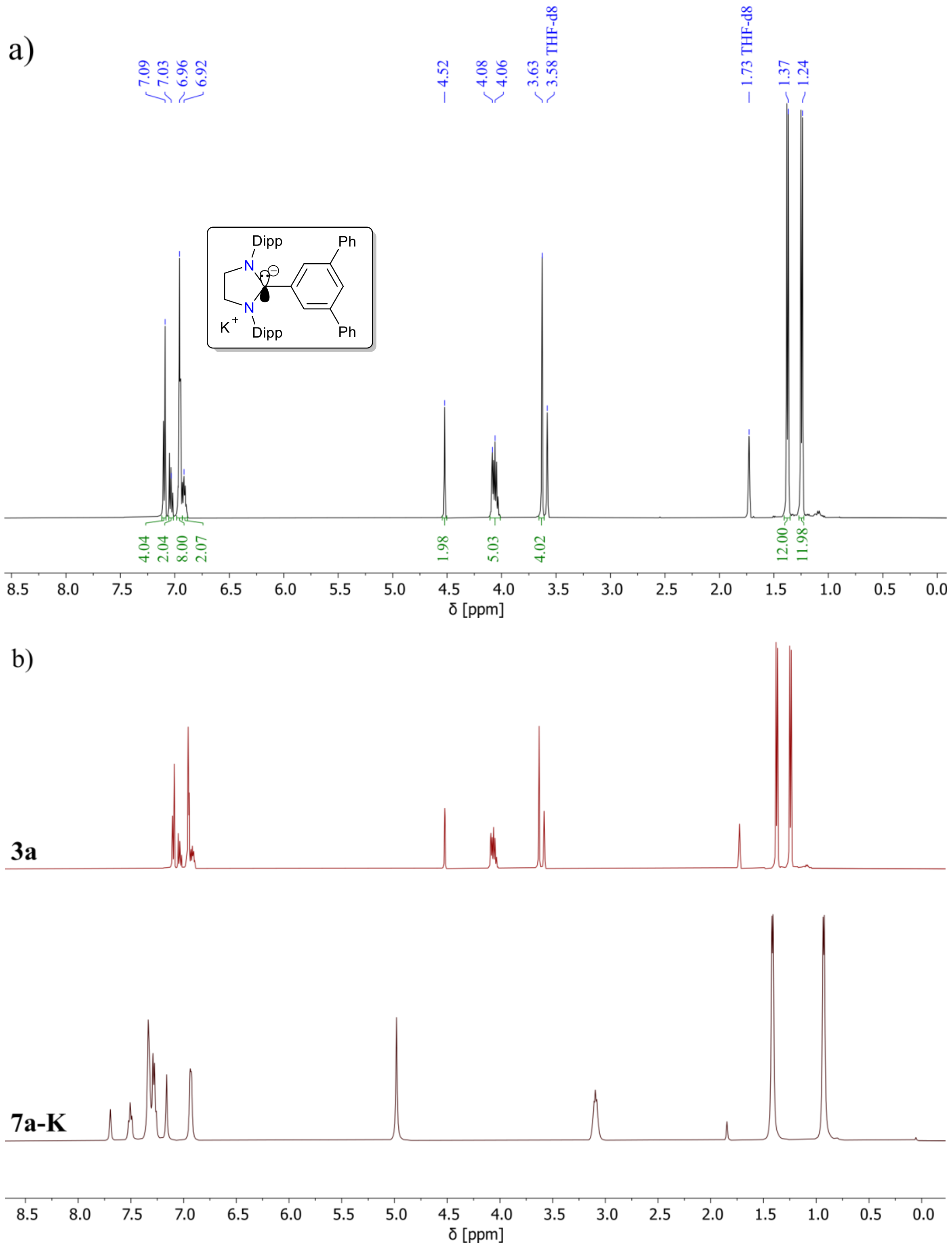

Figure S29. a) ^{1}H NMR (500 MHz, THF-d_8, 298 K) spectrum of **7a-K**. b) Stacked ^{1}H NMR spectra of **3a** (in $CDCl_3$) and **7a-K** (in THF-d_8).

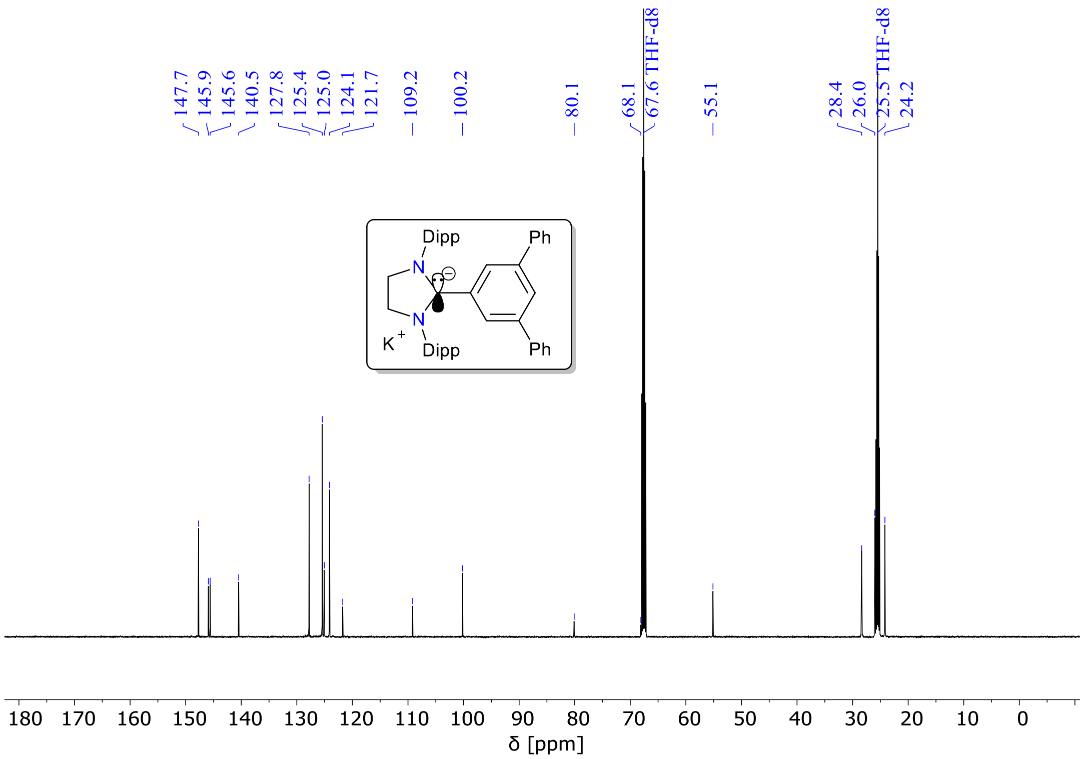

Figure S30. $^{13}C\{^{1}H\}$ NMR (125 MHz, THF-d_8, 298 K) spectrum of **7a-K**.

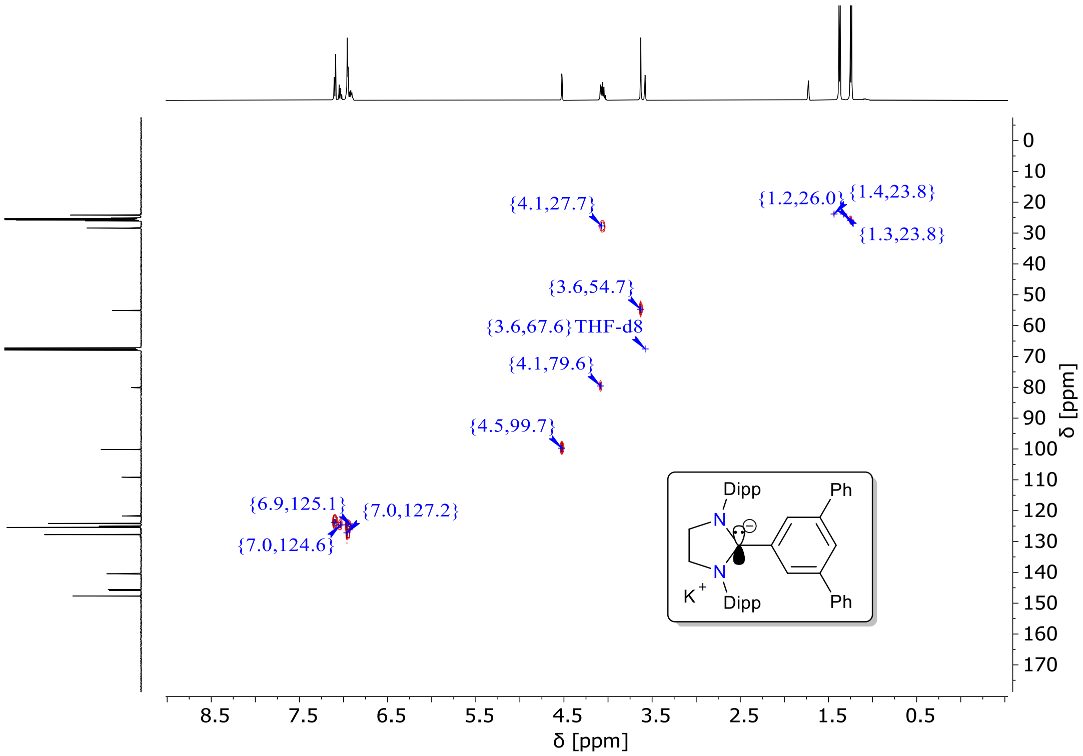

Figure S31. ^{1}H-$^{13}C\{^{1}H\}$ HMQC NMR (500/125 MHz, THF-d_8, 298 K) spectrum of **7a-K**.

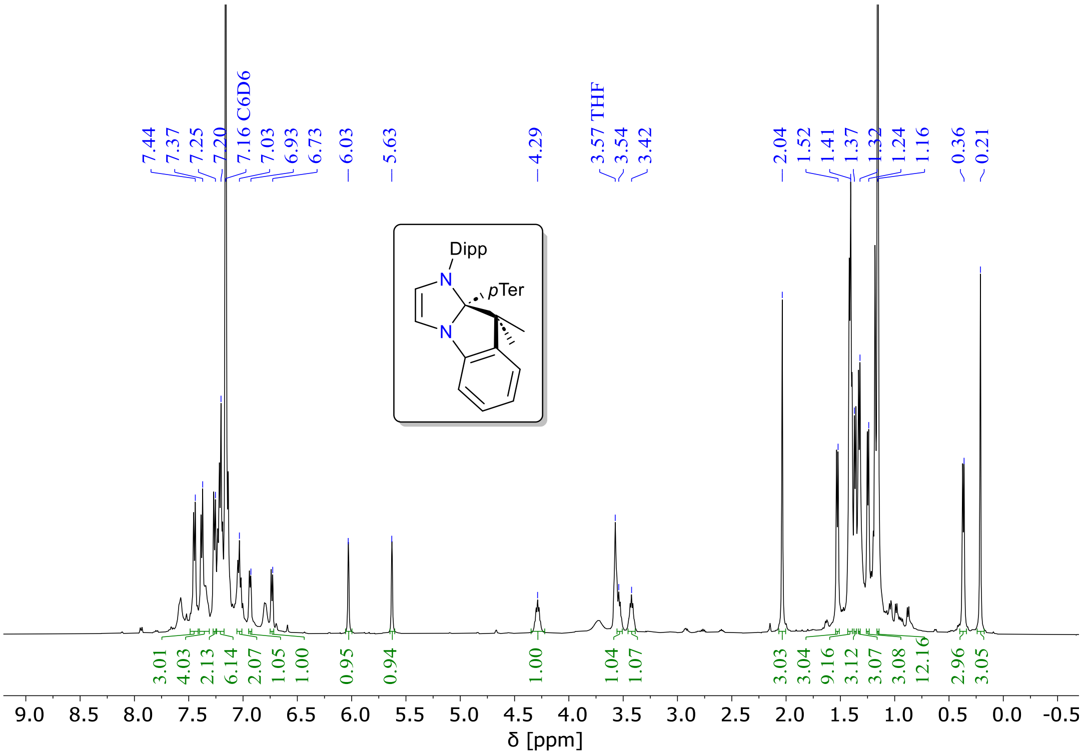

Figure S32. ^{1}H NMR (500 MHz, C_6D_6, 298 K) spectrum of **11**-*p*-Ter and TEMPO.

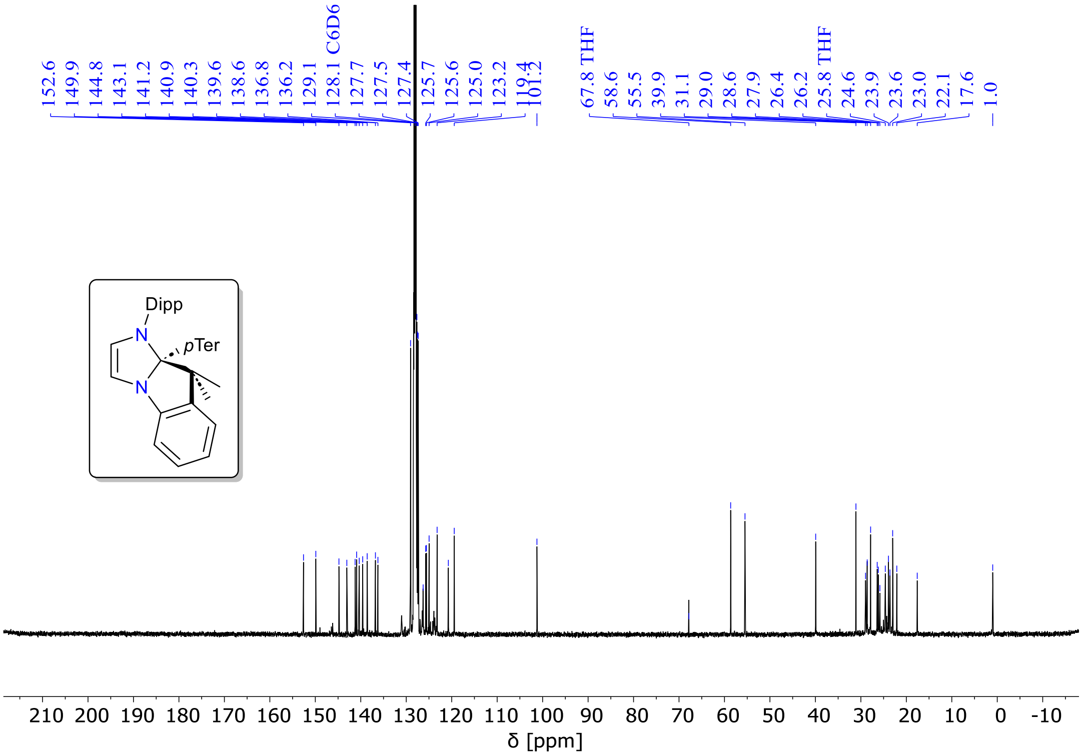

Figure S33. $^{13}C\{^{1}H\}$ NMR (125 MHz, C_6D_6, 298 K) spectrum of **11**-*p*-Ter and TEMPO.

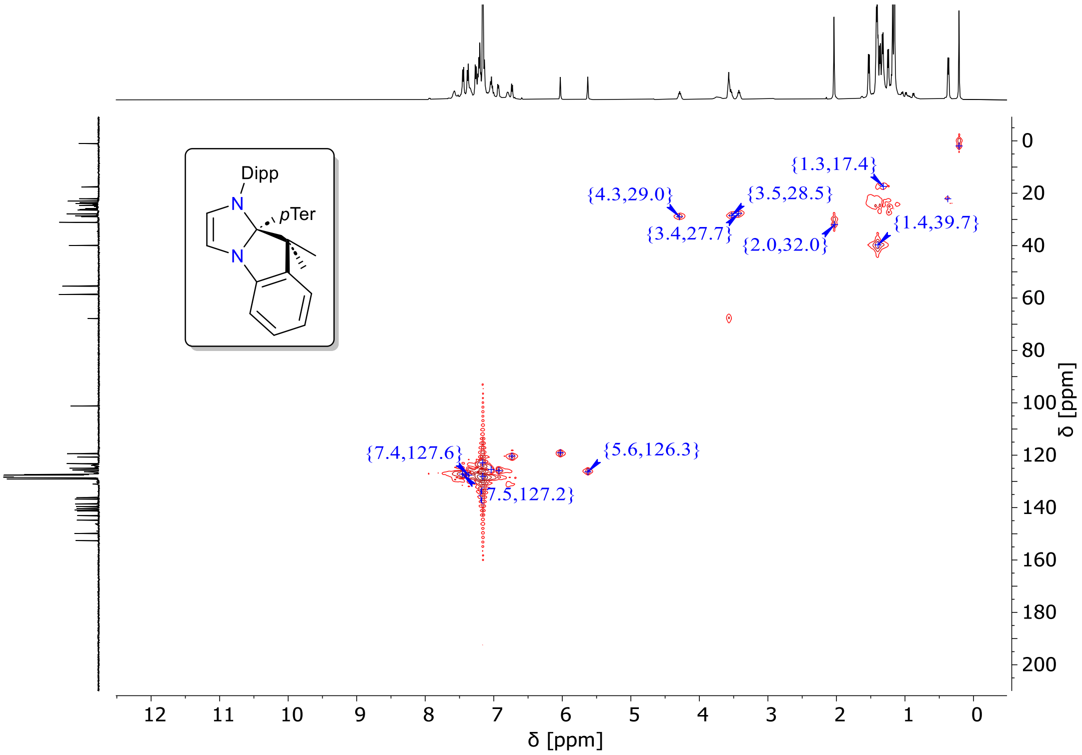

Figure S34. ^{1}H-$^{13}C\{^{1}H\}$ HMQC NMR (500/125 MHz, C_6D_6, 298 K) spectrum of **11-*p***-Ter and TEMPO.

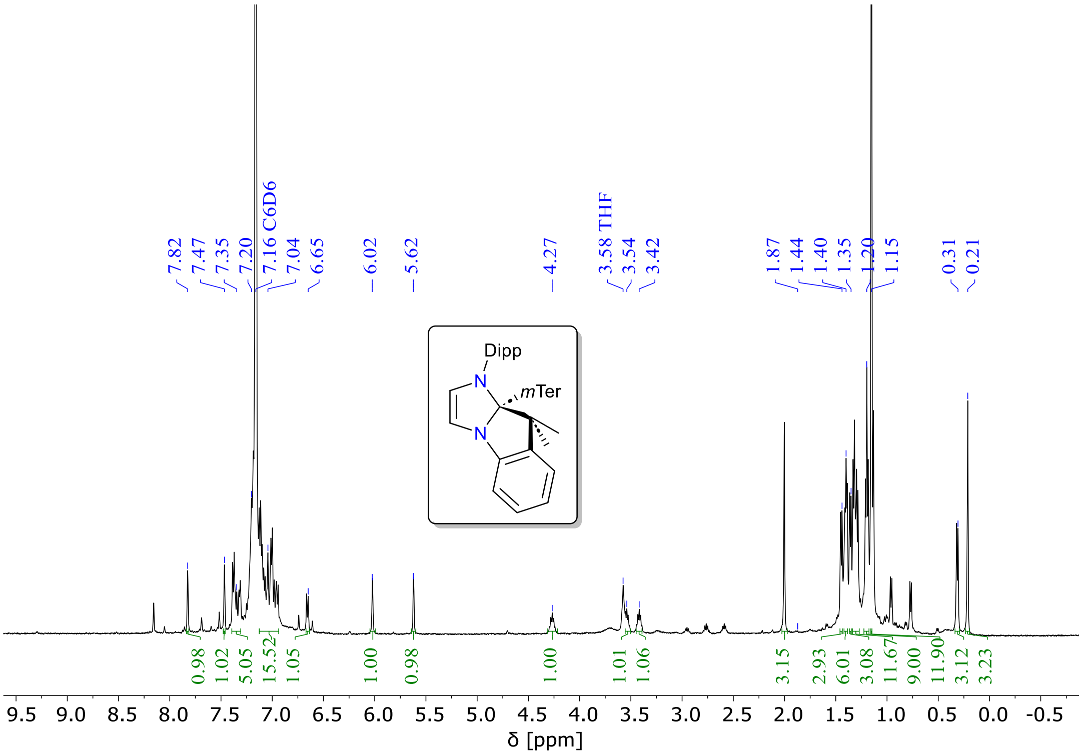

Figure S35. ^{1}H NMR (500 MHz, C_6D_6, 298 K) spectrum of **11-*m***-Ter and TEMPO.

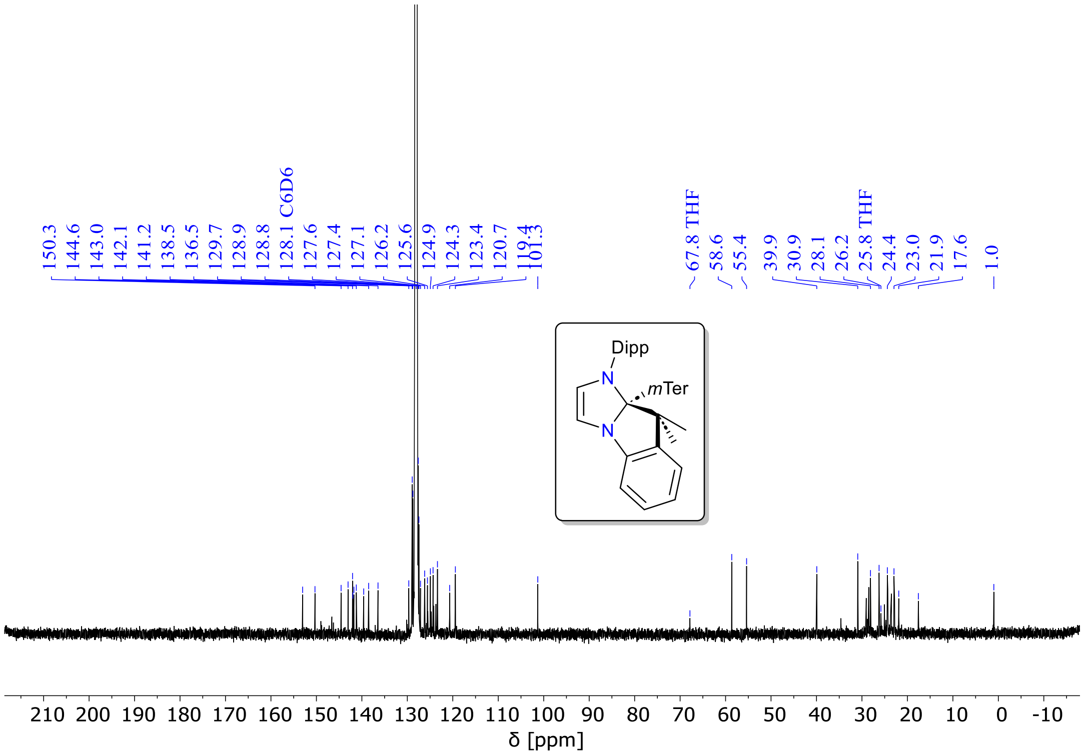

Figure S36. $^{13}C\{^1H\}$ NMR (125 MHz, C_6D_6, 298 K) spectrum of **11**-*m*-Ter and TEMPO.

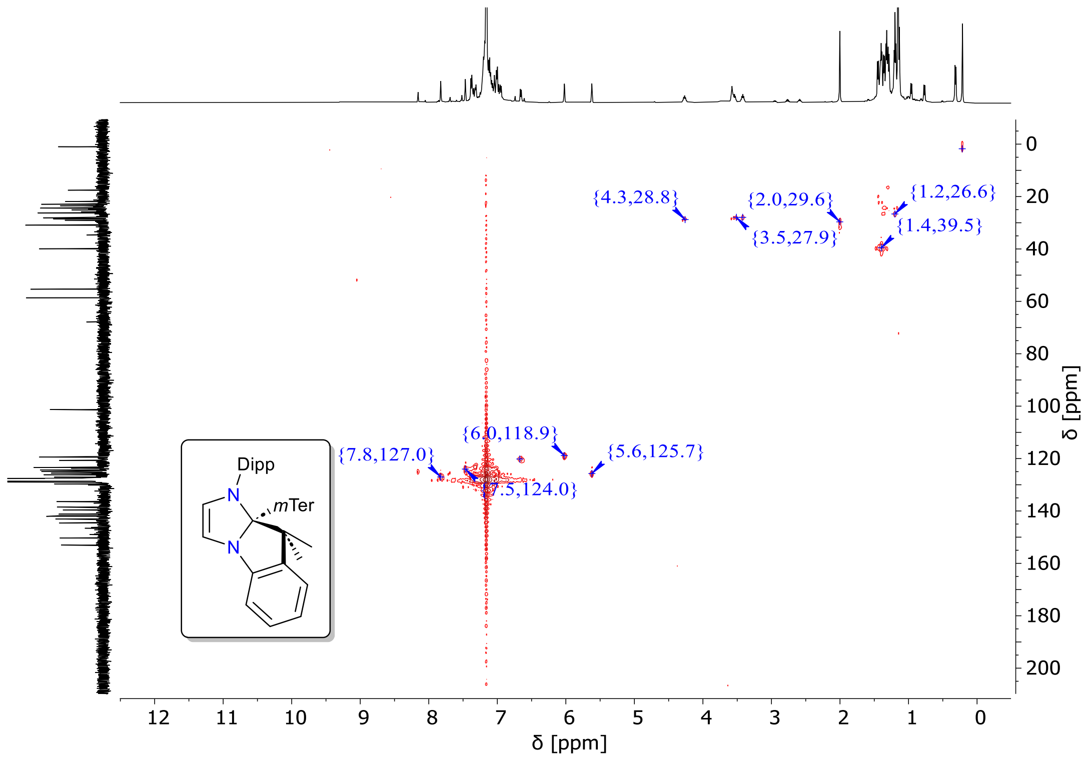

Figure S37. ^1H-$^{13}C\{^1H\}$ HMQC NMR (500/125 MHz, C_6D_6, 298 K) spectrum of **11**-*m*-Ter and TEMPO.

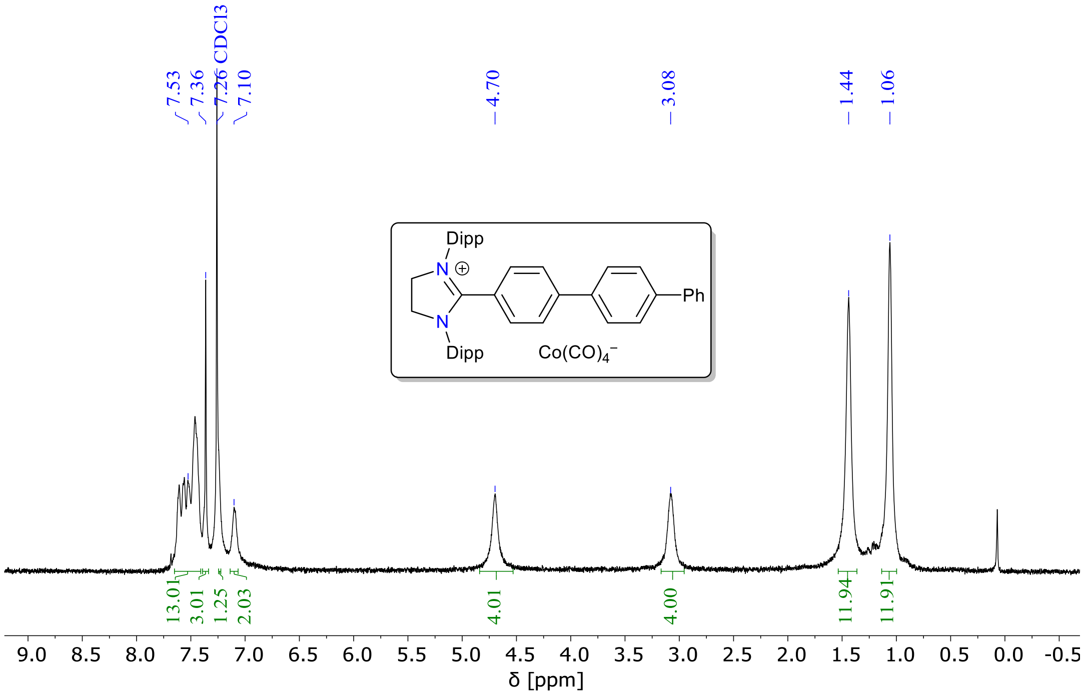

Figure S38. ^{1}H NMR (500 MHz, C_6D_6, 298 K) spectrum of [**2a**][$Co(CO)_4$].

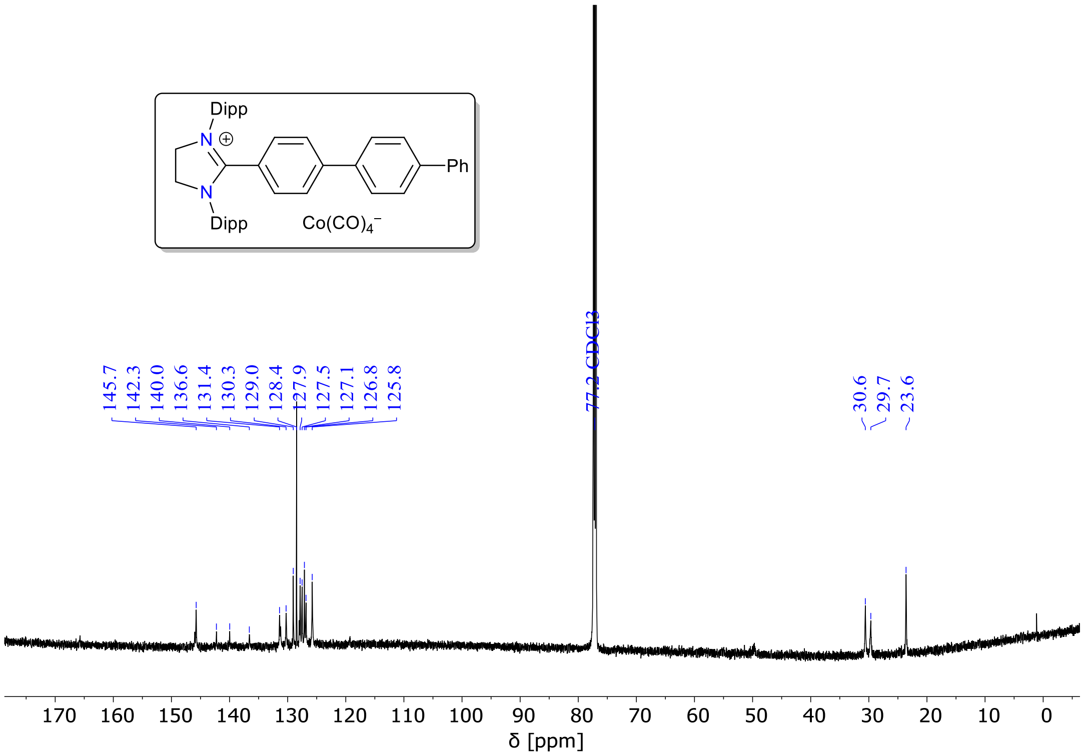

Figure S39. $^{13}C\{^{1}H\}$ NMR (125 MHz, C_6D_6, 298 K) spectrum of [**2a**][$Co(CO)_4$].

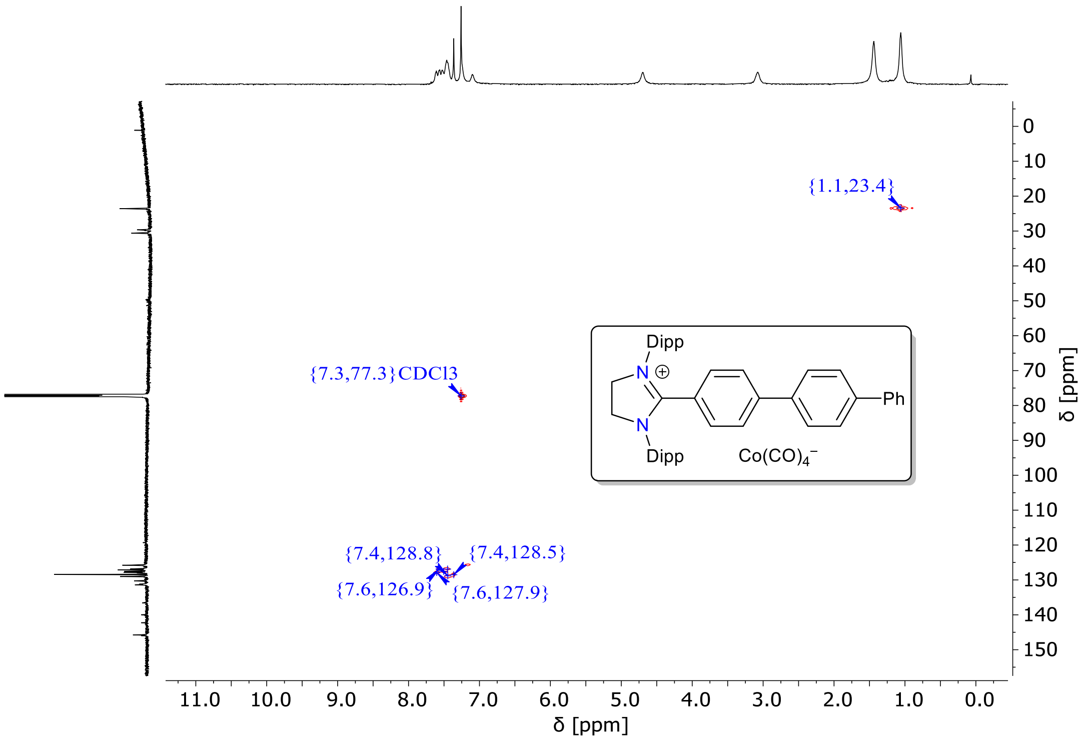

Figure S40. ^{1}H-$^{13}C\{^{1}H\}$ HMQC NMR (500/125 MHz, C_6D_6, 298 K) spectrum of [**2a**][$Co(CO)_4$].

UV-Visible Spectra

UV-visible spectra were recorded on a Genesys 50 UV-visible spectroscopy system by thermos scientific. A quartz glass cuvette (1cm) was used for the solved samples. The device was blanked with pure solvant before each measurement. Higher concentrations were used when necessary, resulting in A > 1, to observe previously hidden absorption bands.

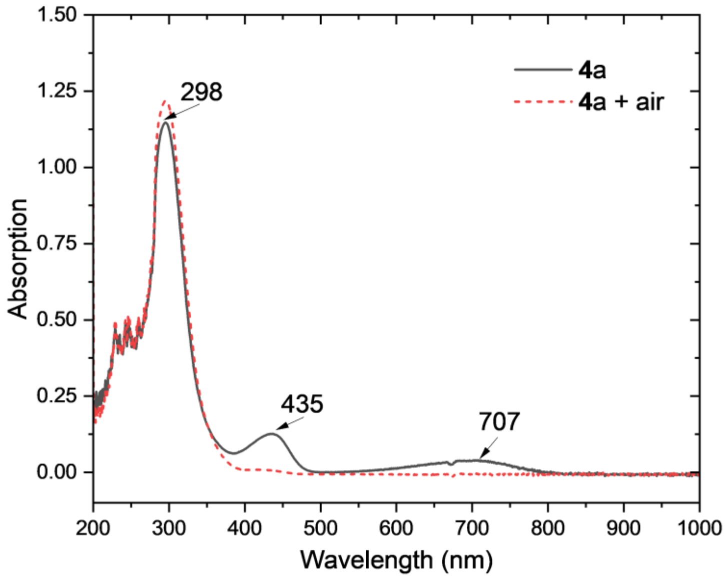

Figure S41. UV-visible spectra of [(SIPr)*p*-Ter] (**4a**) measured in toluene (69 µM). Observed absorption bands and calculated molar extinction coefficients ε (L mol^{-1} cm^{-1}): [λ_1 = 298 nm (16522); λ_2 = 435 nm (1884); λ_3 = 707 nm (580)].

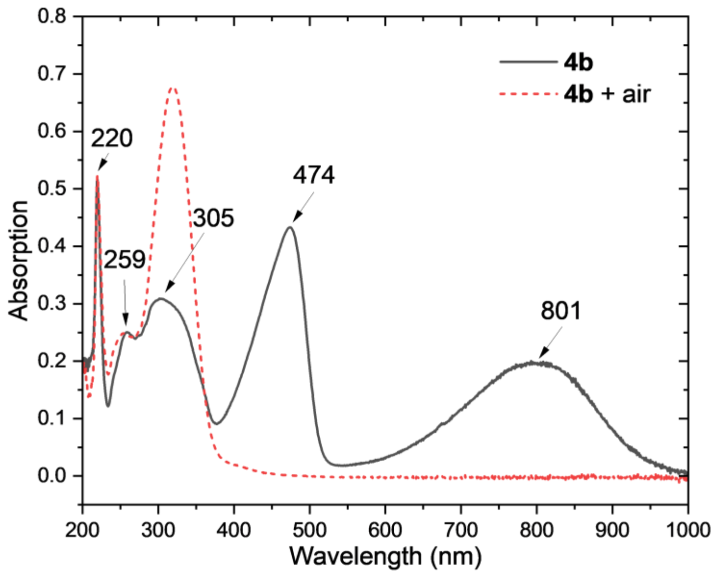

Figure S42. UV-visible spectra of [(IPr)*p*-Ter] (**4b**) measured in toluene(40 µM). Observed absorption bands and calculated molar extinction coefficients ε (L mol^{-1} cm^{-1}): [λ_1 = 259 nm (6250); λ_2 = 305 nm (7750); λ_3 = 474 nm (10750); λ_4 = 801 nm (5000)].

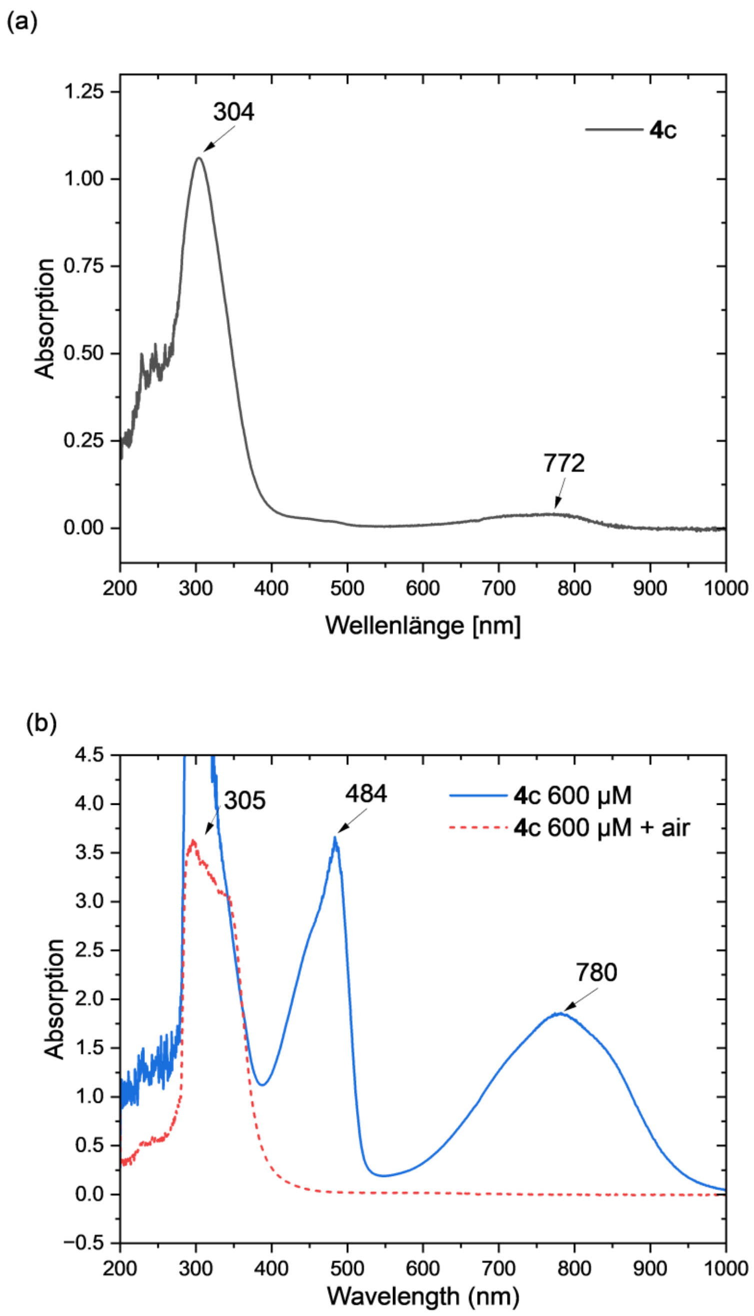

Figure S43. (a) UV-visible spectra of [(Me-IPr)*p*-Ter] (**4c**) measured in toluene at 65 µM and (b) at 600 µM. Observed absorption bands (for 65 µM) and calculated molar extinction coefficients ε (L mol^{-1} cm^{-1}) from (a): [λ_1 = 304 nm (16308); λ_2 = 772 nm (615)].

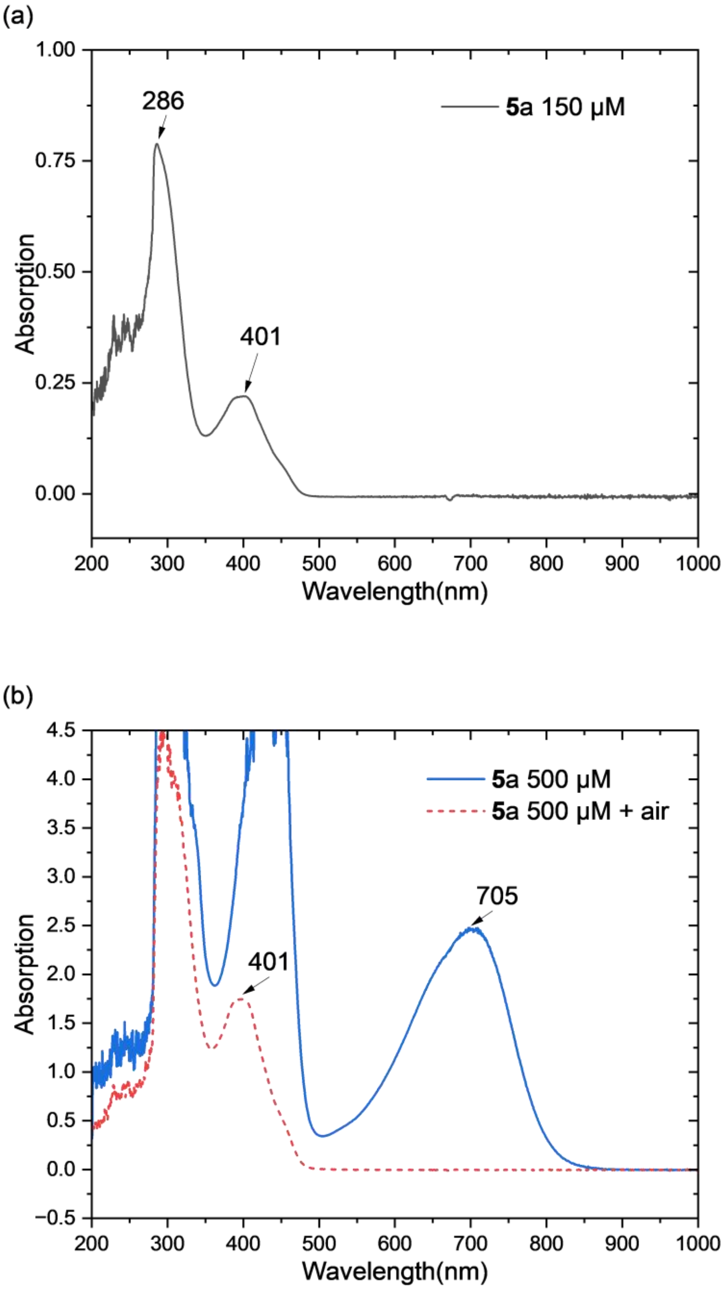

Figure S44. (a): UV-visible spectra of [(SIPr)*m*-Ter] (**5a**) measured in toluene 150µM and (b) 500µM. Observed absorption bands (for 150 µM) and calculated molar extinction coefficients ε (L mol^{-1} cm^{-1}): [λ_1 = 286 nm (5267); λ_2 = 401 nm (1466)].

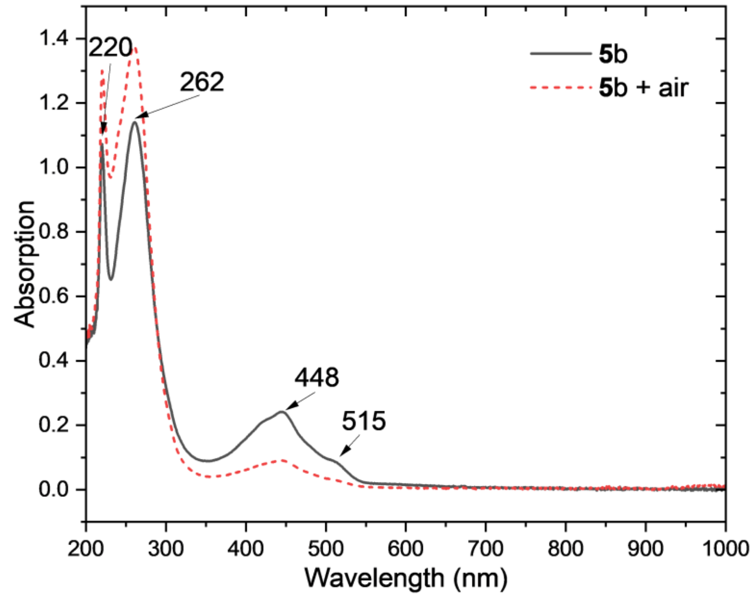

Figure S45. UV-visible spectra of [(IPr)*m*-Ter] (**5b**) measured in toluene (80 µM). Observed absorption bands and calculated molar extinction coefficients ε (L mol^{-1} cm^{-1}): [λ_1 = 220 nm (13375); λ_2 = 262 nm (14250); λ_3 = 448 nm (3000); λ_4 = 515 nm (1000)].

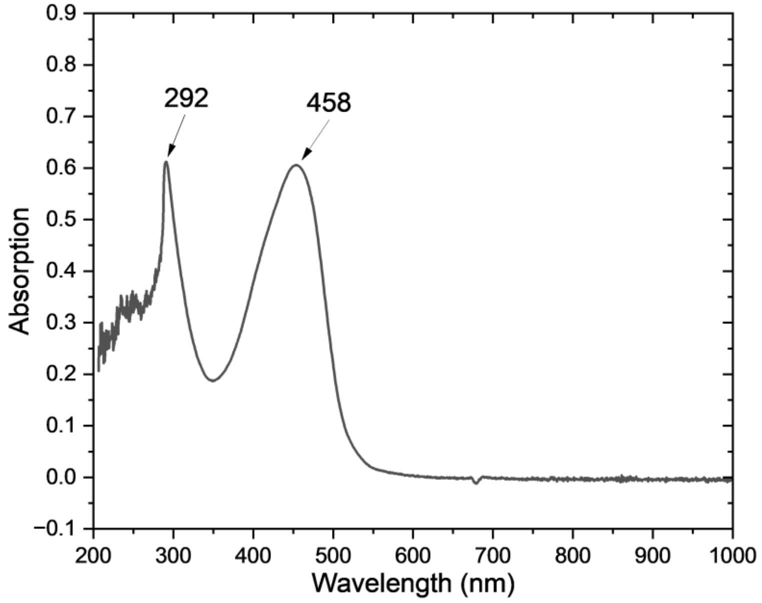

Figure S46. UV-visible spectra of [(MeIPr)*m*-Ter] (**5c**) measured in toluene (80 µM). Observed absorption bands and calculated molar extinction coefficients ε (L mol^{-1} cm^{-1}): [λ_1 = 292 nm (7500); λ_2 = 458 nm (7500)].

Cyclic Voltammetry

Cyclic voltammetry (CV) experiments were carried out using a PGSTAT204 electrochemical workstation (METROHM). All experiments were carried out under an atmosphere of argon in degassed and anhydrous 5 mL acetonitrile solution containing nBu$_4$NPF$_6$ (0.1 M) at a scan rates of 50, 100, 250, 500, and 1000 mV s^{-1}. The setup consisted of a glassy carbon working electrode (surface area = 0.04 cm^2), a graphite counter electrode, and a silver wire as the pseudo-reference electrode. First, ferrocene was measured in an acetonitrile solution of nBu$_4$NPF$_6$ and observed at $E_{1/2}$ (Fc/Fc$^+$) = +0.44 V. Adding ferrocene to a solution of nBu$_4$NPF$_6$ and one of the samples led to a shift of the observed Fc/Fc$^+$ to $E_{1/2}$ = 0.80 V. Using Fc/Fc$^+$ (ferrocene/ferrocenium) couple as a reference, we set $E_{1/2}$(Fc/Fc$^+$) = 0.4 V.[7] For irreversible reduction waves, only the cathodic peak potential E_{pc} is given.

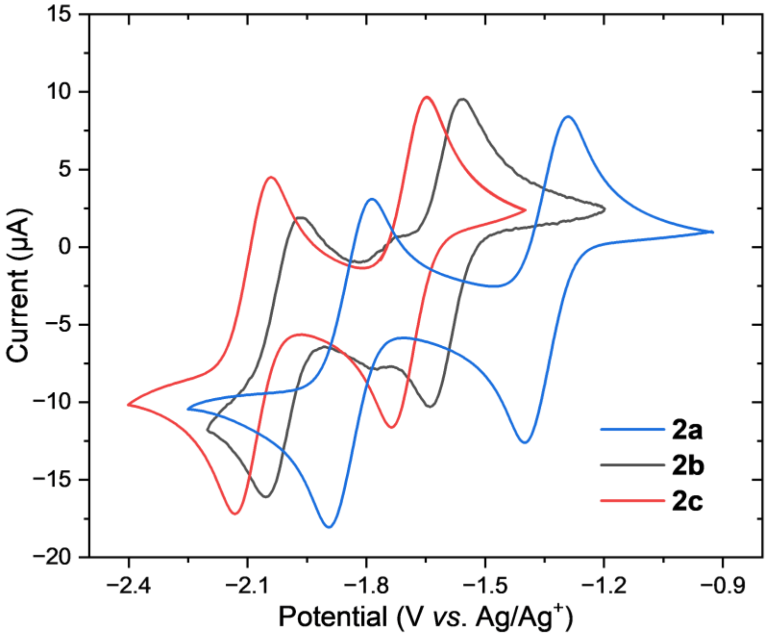

Figure S47. Stack of CVs of **2a**–**c** at 100 mV s^{-1}. (**2**a: $E_{1/2}$ = −1.35 V, $E_{1/2}$ = −1.85 V; **2**b: $E_{1/2}$ = −1.60 V, $E_{1/2}$ = −2.00 V; **2**c: $E_{1/2}$ = −1.70 V, $E_{1/2}$ = −2.10 V).

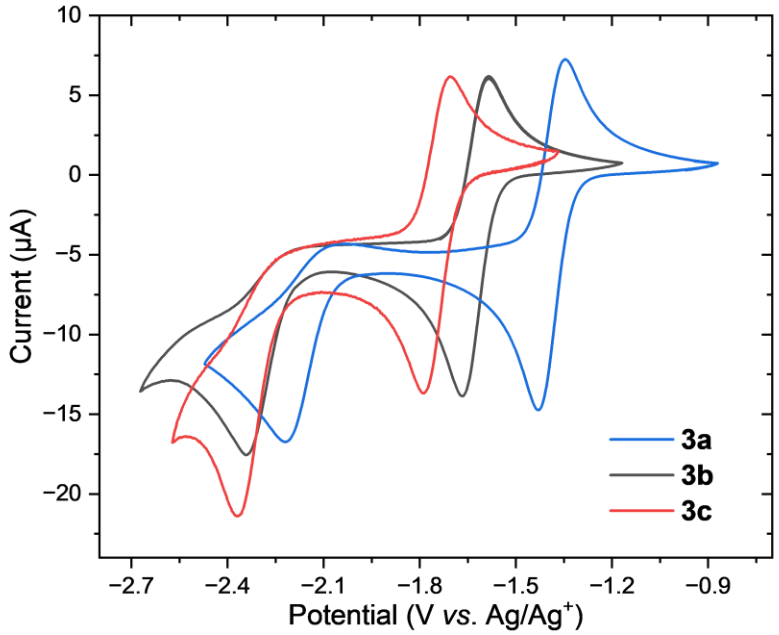

Figure S48. Stack of CVs of **3a–c** at 100 mV s^{-1}. (**3a**: $E_{1/2} = −1.40$ V, $E_{pc} = −2.20$ V; **3b**: $E_{1/2} = −1.65$ V, $E_{pc} = −2.30$ V; **3c**: $E_{1/2} = −1.75$ V, $E_{pc} = −2.35$ V).

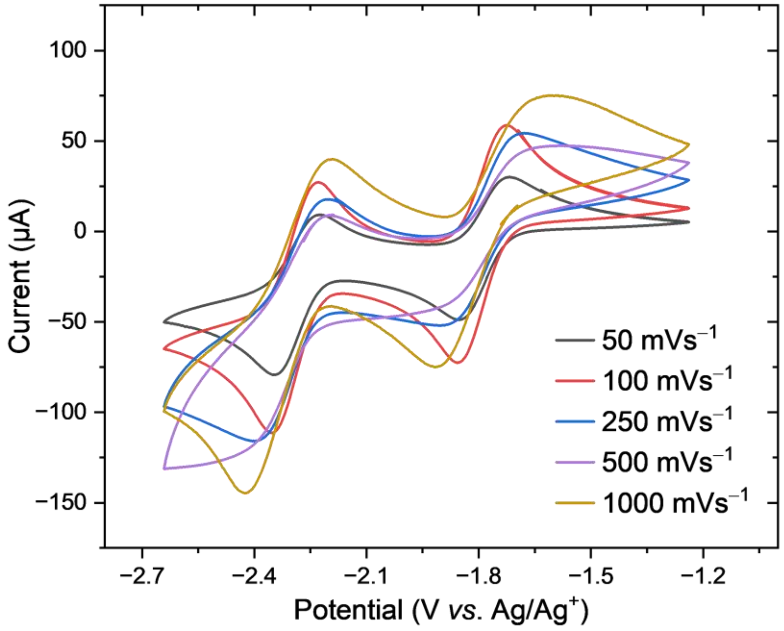

Figure S49. Stack of CVs of **2a** at various scan rates.

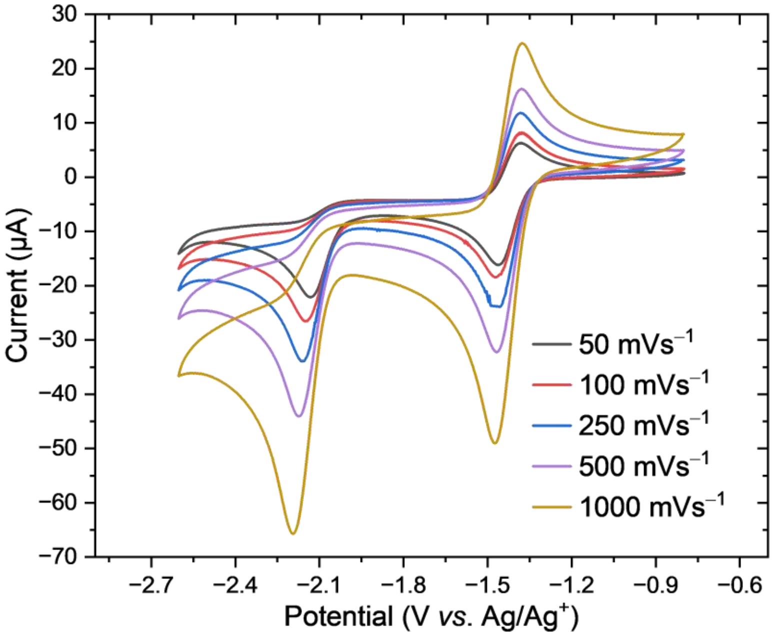

Figure S50. Stack of CVs of **3a** at various scan rates.

EPR Spectroscopy

The continuous wave (CW) EPR experiments were performed at room temperature (298 K) in a Bruker standard ST9402 resonator and with a Bruker EMX Plus spectrometer. The microwave frequency was 9.63 GHz and the modulation amplitude was 0.3 mT. Simulations of the EPR spectra were performed by iteration of the isotropic *g*-values, hyperfine coupling interactions and line widths using EasySpin (version 6.0.2)[8] via the cwEPR GUI (slightly modified version of v3.4.6.47.[9]

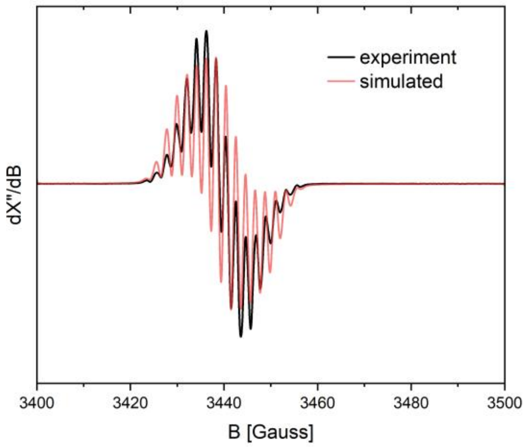

Figure S51. Isotropic X-band EPR spectrum of [(SIPr)*p*-Ter] (**4a**) recorded at RT in toluene. HFC were found to be of 2 equivalent N atoms (11.2 MHz), a set of 4 equivalent H atoms (6.1 MHz), a set of 2 equivalent H atoms (7.3 MHz) and a single H atom (5.9 MHz)

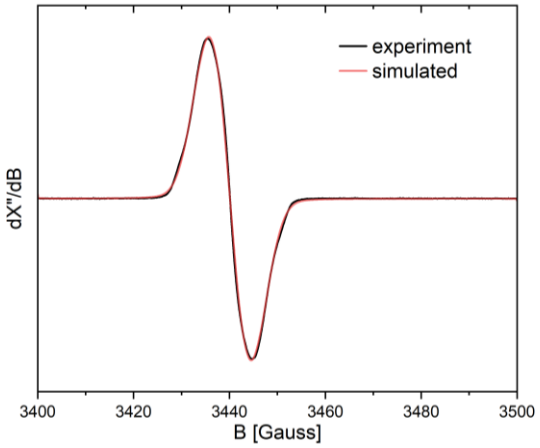

Figure S52. Isotropic X-band EPR spectrum of [(IPr)*p*-Ter] (**4b**) recorded at RT in toluene. HFC were found to be 2 equivalent N atoms (6.4 MHz), a set of 2 equivalent H atoms (6.9 MHz), another set of 2 equivalent H atoms (6.0) and a single H atom (6.0 MHz). However, these are not at all resolved. The only reliable information is the g-value.

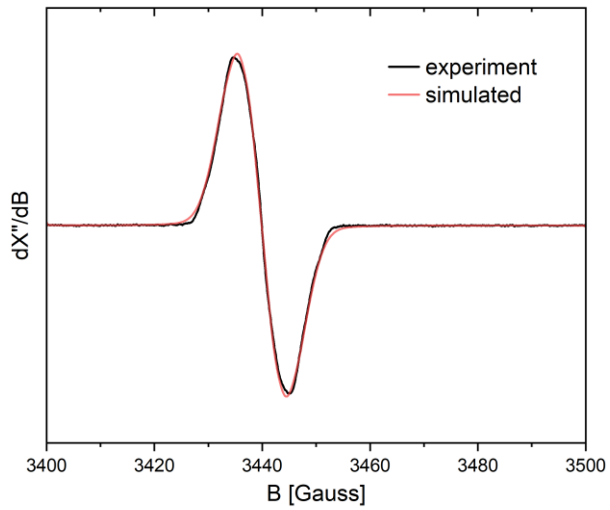

Figure S53. Isotropic X-band EPR spectrum of [(Me-IPr)*p*-Ter] (**4c**) recorded at RT in toluene. HFC were found to be 2 equivalent N atoms (6.9 MHz), a set of 2 equivalent H atoms (6.8 MHz), another set of 2 equivalent H atoms (6.0 MHz) and a single H atom (6.0 MHz). However, these are not at all resolved. The only reliable information is the g-value.

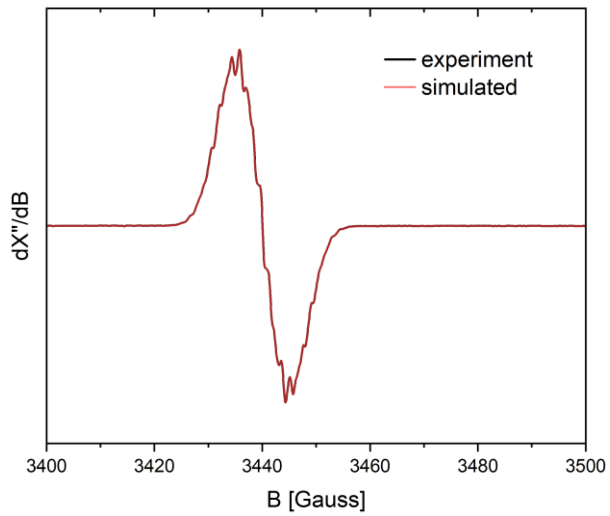

Figure S54. Isotropic X-band EPR spectrum of [(SIPr)*m*-Ter] (**5a**) recorded at RT in toluene. HFC were found to be 2 equivalent N atoms (7.6 MHz), a set of 4 equivalent H atoms (3.9 MHz), a set of 2 equivalent H atoms (8.8 MHz), another set of 2 equivalent H atoms (6.5 MHz) and a single H atom (6.0 MHz). Hyperfine couplings are not very reliable, as they are not so well resolved.

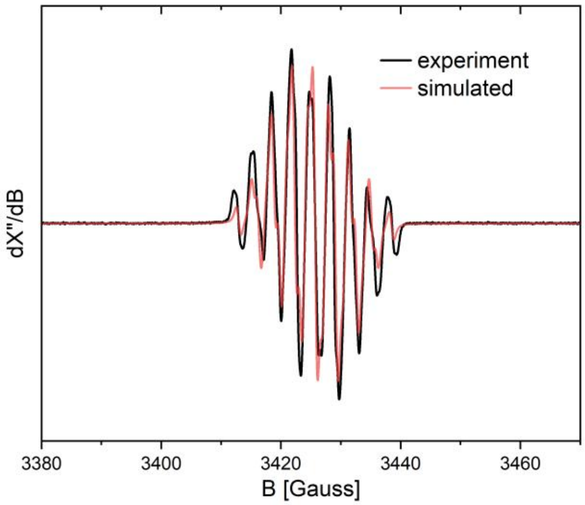

Figure S55. Isotropic X-band EPR spectrum of [(IPr)*m*-Ter] (**5b**) recorded at RT in toluene. HFC were found to be of 2 equivalent N atoms (9.9 MHz), a set of 2 equivalent H atoms (9 MHz), and another set of 2 equivalent H atoms (7 MHz).

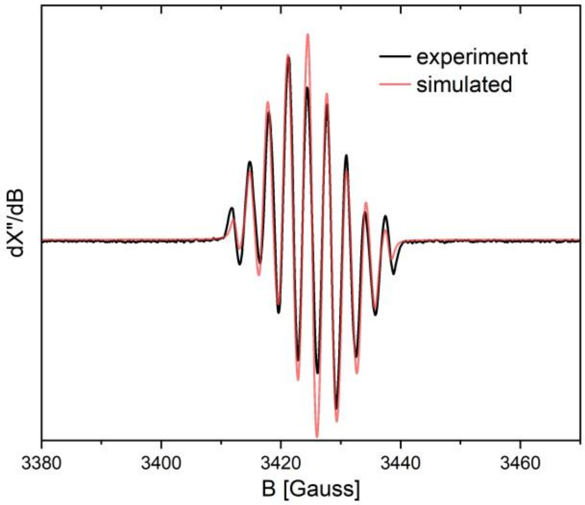

Figure S56. Isotropic X-band EPR spectrum of [(Me-IPr)*m*-Ter] (**5c**) recorded at RT in toluene. HFC were found to be of 2 equivalent N atoms (10 MHz), a set of 2 equivalent H atoms (8.5 MHz), and another set of 2 equivalent H atoms (7 MHz).

Table S1. Collection of EPR parameters of **4a**–**c** and **5a**–**c**.

	**4a**	**4b**	**4c**	**5a**	**5b**	**5c**
g-Tensor rt	2.00285	2.00292	2.00302	2.00296	2.00270	2.002768
lwpp	[0.1, 0.06]	[0.3, 0.056]	[0.3, 0.056]	[0.08, 0.03]	[0.05, 0.05]	[0.06, 0.06]
Nucs	N, H, H, H	N, H, H, H	N, H, H, H	N, H, H, H, H	N, H, H	N, H, H
n	[2,4,2,1]	[2,2,2,1]	[2,2,2,1]	[2,4,2,2,1]	[2,2,2]	[2,2,2]
A-Tensor rt	[11.2, 6.1, 7.3, 5.9]	[6.4, 6.9, 6, 6]	[6.9, 6.8, 6, 6]	[7.6, 3.9, 8.8, 6.5, 6]	[9.9, 9, 7]	[10, 8.5, 7]

n = number of equivalent nuclei

Crystallographic Details

Single crystals were examined on a Rigaku Supernova diffractometer using CuKα (λ = 1.54184 Å) radiation. The crystals were kept at 100.0(1) K during data collection. Using Olex2,[10] the structures were solved with the ShelXT[11] structure solution programs using Intrinsic Phasing (**2a**, **2b**, **2c**, **3a**, **4a**, **4b**, **4c**, **5a**, **11**-*p*-Ter, **11**-*m*-Ter, [**2a**][$Co(CO)_4$]) and refined with the ShelXL[12] (**2a**, **2b**, **2c**, **3a**, **4b**, **4c**, **11**-*p*-Ter, **11**-*m*-Ter, [**2a**][$Co(CO)_4$]) refinement package using Least Squares minimisation or with olex2.refine[13] (**4a**, **5a**) using Gauss-Newton minimization and NoSpherA2.[14]

For **2b**, the crystal contains heavily disordered dichloromethane molecules, which could not be refined reasonably. Therefore, a solvent mask was calculated and 280 electrons were found in a volume of 1041 $Å^3$ in 1 void per unit cell. This is consistent with the presence of 1.75 dichloromethane molecules per asymmetric unit which account for 294 electrons per unit cell. For **2c**, a solvent mask was calculated and 84 electrons were found in a volume of 492 $Å^3$ in 2 voids per unit cell. This is consistent with the presence of 0.5 THF molecules per asymmetric unit which accounts for 80 electrons per unit cell.

For **3a**, a solvent mask was calculated and 259 electrons were found in a volume of 920 $Å^3$in 1 void per unit cell. This is consistent with the presence of 1 dichloromethane and 0.5 THF molecules per asymmetric unit which account for 264 electrons per unit cell.

For **4b**, the Dipp groups at C79 and C90 are disordered over two sites in a 65:35 ratio. Suitable restraints were applied for the disordered atoms. For **4c**, A solvent mask was calculated and 301 electrons were found in a volume of 1384 $Å^3$ in 1 void per unit cell. This is consistent with the presence of 1.5 hexane molecules per asymmetric unit which account for 300 electrons per unit cell.

For **5a**, the position of C7 and its hydrogen atom cannot be suitably modeled with only one type of carbon atom, neither with an aromatic one nor with a non-aromatic one. The residual electron density is banana-shaped and is perpendicular to the aromatic plane, which indicates a carbanionic component (Figure S57). C7 was modelled to be positionally disordered (ratio 64:36), the thermal parameters of the disordered hydrogen atom H7A and H7B were constrained to be same. All other hydrogen atoms were refined isotropically.

Details of the X-ray investigations are given in Table S2–S5. CCDC 2471666 (**2a**), 2471667 (**2b**), 2471668 (**2c**), 2471669 (**3a**), 24716677 (**4a**), 24716670 (**b**), 24716671 (**c**), 24716672 (**5a**), 24716673 (**11**-*p*-Ter), 24716674 (**11**-*m*-Ter) and 24716675 ([**2a**][$Co(CO)_4$]) contain the supplementary crystallographic data for this paper. These data can be obtained free of charge from The Cambridge Crystallographic Data Centre via www.ccdc.cam.ac.uk/structures.

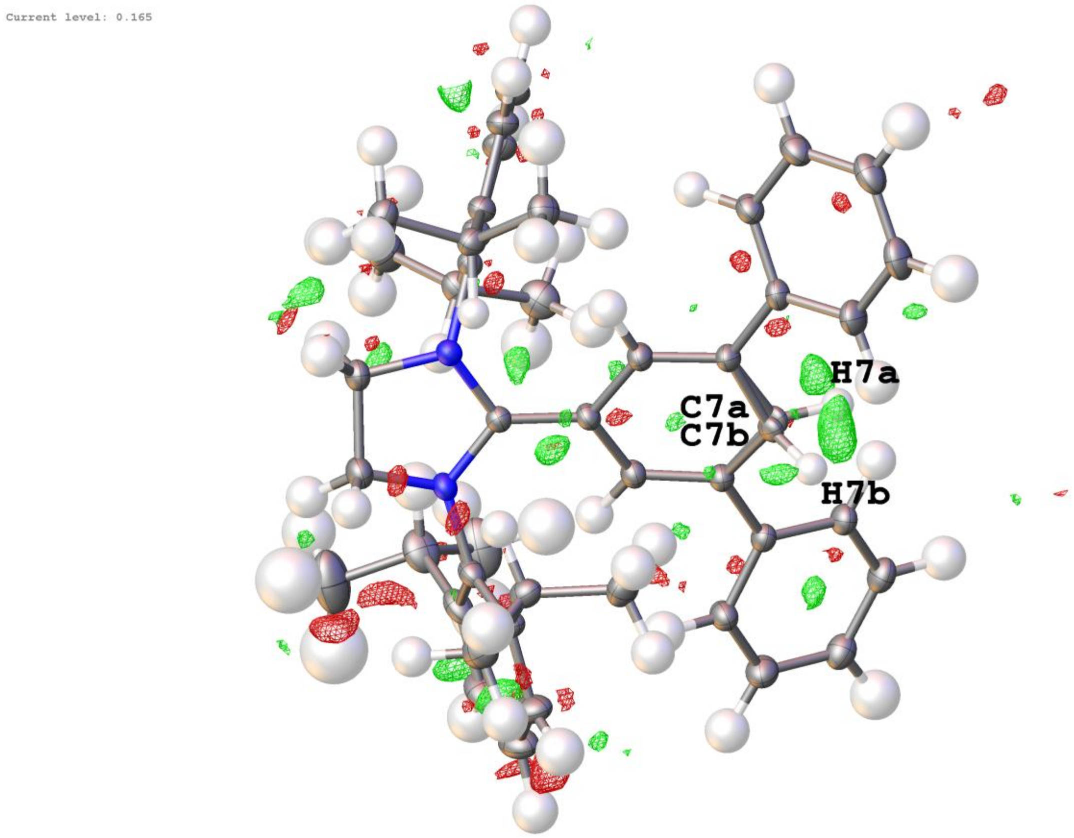

Figure S57. Olex2 electron maps with residual electron density of **5a**.

Table S2. Crystallographic details of **2a**, **2b**, and **2c**.

	**2a**	**2b(1.75 CH_2Cl_2)**	**2c(1 C_6H_6, 0.5 C_4H_8O)**
**Empirical formula**	$C_{45}H_{51}BrN_2$	$C_{46.75}H_{52.5}BrCl_{3.5}N_2$	$C_{55}H_{63}BrN_2O_{0.5}$
**Formula weight**	699.79	846.39	839.98
**Temperature [K]**	100.0(1)	100.0(1)	100.0(1)
**Crystal system**	triclinic	triclinic	monoclinic
**Space group**	P-1	P-1	$P2_1/c$
**a [Å]**	10.2803(5)	15.2926(3)	17.0614(3)
**b [Å]**	13.9114(7)	16.2532(3)	16.7345(2)
**c [Å]**	15.2962(7)	19.5873(4)	18.4085(3)
**α [°]**	69.243(4)	74.751(2)	90
**β [°]**	71.740(4)	74.567(2)	116.248(2)
**γ [°]**	71.966(4)	74.603(2)	90
**Volume [$Å^3$]**	1893.39(18)	4426.54(17)	4713.94(15)
Z	2	4	4
**ρ_{calc} [g/cm^3]**	1.227	1.270	1.184
**μ [mm^{-1}]**	1.714	1.173	1.467
**F(000)**	740.0	1766.0	1784.0
**Crystal size [mm^3]**	0.26 × 0.23 × 0.07	0.383 × 0.234 × 0.14	0.21 × 0.08 × 0.05
**Radiation (λ [Å])**	Cu Kα (λ = 1.54184)	Mo Kα (λ = 0.71073)	Cu Kα (λ = 1.54184)
**2Θ range for data [°]**	6.348 to 147.804	5.314 to 60.068	5.776 to 147.944
**hkl-index ranges**	$-12 \leq h \leq 12$<br>$-16 \leq k \leq 17$<br>$-18 \leq l \leq 13$	$-21 \leq h \leq 21$,<br>$-22 \leq k \leq 22$,<br>$-27 \leq l \leq 27$	$-21 \leq h \leq 14$,<br>$-17 \leq k \leq 20$,<br>$-22 \leq l \leq 22$
**Reflections collected**	14150	102936	36135
**Independent reflections**	7429 [Rint = 0.0221,<br>Rsigma = 0.0273]	25865 [R_{int} = 0.0330,<br>R_{sigma} = 0.0304]	9466 [R_{int} = 0.0195,<br>R_{sigma} = 0.0160]
**Reflections with *I* > 2σ(*I*)**	6773	21205	8748
**Data/restraints/parameters**	7429/0/441	25865/0/881	9466/0/515
***GoF* on F^2**	1.022	1.037	1.059
**R_1/wR_2 [$I > 2\sigma(I)$]**	R1 = 0.0319,<br>wR2 = 0.0793	R_1 = 0.0378,<br>wR_2 = 0.0998	R_1 = 0.0264,<br>wR_2 = 0.0685
**R_{ind} [all data]/wR_2**	R1 = 0.0356,<br>wR2 = 0.0820	R_1 = 0.0491,<br>wR_2 = 0.1048	R_1 = 0.0288,<br>wR_2 = 0.0701
**ρ_{fin} (max/min) / [e $Å^{-3}$]**	0.61/−0.52	0.65/−0.48	0.59/−0.28
**CCDC number**	2471666	2471667	2471668

Table S3. Crystallographic details of **3a**, **4a**, and **4b**.

	**3a(0.5 $C_4H_8O_2$, 1 CH_2Cl_2)**	**4a**	**4b**
**Empirical formula**	$C_{48}H_{57}Br Cl_2N_2O$	$C_{45}H_{51}N_2$	$C_{45}H_{49}N_4$
**Formula weight**	828.76	619.920	617.86
**Temperature [K]**	100.0(1)	100.0(1)	100.0(1)
**Crystal system**	triclinic	monoclinic	monoclinic
**Space group**	P-1	$P2_1/c$	$P2_1/n$
**a [Å]**	15.6189(5)	18.7119(2)	10.7275(4)
**b [Å]**	15.7783(5)	13.1283(1)	48.6244(16)
**c [Å]**	21.6246(6)	15.7741(2)	14.2444(5)
**α [°]**	71.990(3)	90	90
**β [°]**	87.785(3)	109.831(1)	102.935(3)
**γ [°]**	61.453(3)	90	90
**Volume [$Å^3$]**	4413.8(3)	3645.20(7)	7241.6(4)
Z	4	4	8
**ρ_{calc} [g/cm^3]**	1.247	1.130	1.133
**μ [mm^{-1}]**	2.654	0.486	0.489
**F(000)**	1744.0	1343.7	2664.0
**Crystal size [mm^3]**	0.26 × 0.21 × 0.14	0.17 × 0.16 × 0.14	0.35 × 0.14 × 0.09
**Radiation (λ [Å])**	Cu Kα (λ = 1.54184)	Cu Kα (λ = 1.54184)	Cu Kα (λ = 1.54184)
**2Θ range for data [°]**	6.498 to 148.21	5.02 to 152.94	6.622 to 133.2
**hkl-index ranges**	$-16 \leq h \leq 19$, $-13 \leq k \leq 19$, $-25 \leq l \leq 26$	$-22 \leq h \leq 23$, $-16 \leq k \leq 16$, $-19 \leq l \leq 19$	$-12 \leq h \leq 12$, $-57 \leq k \leq 57$, $-16 \leq l \leq 16$
**Reflections collected**	35117	100066	113815
**Independent reflections**	17417 [R_{int} = 0.0252, R_{sigma} = 0.0309]	7638 [R_{int} = 0.0760, R_{sigma} = 0.0241]	12792 [R_{int} = 0.0888, R_{sigma} = 0.0395]
**Reflections with *I* > 2*σ(I)***	15218	6600	8324
**Data/restraints/parameters**	17417/0/891	7638/12/593	12792/528/952
***GoF* on F^2**	1.017	1.055	1.025
**R_1/wR_2 [$I > 2\sigma(I)$]**	R_1 = 0.0378, wR_2 = 0.0964	R_1 = 0.0262, wR_2 = 0.0664	R_1 = 0.0649, wR_2 = 0.1764
**R_{ind} [all data]/wR_2**	R_1 = 0.0437, wR_2 = 0.1002	R_1 = 0.0323, wR_2 = 0.0699	R_1 = 0.0995, wR_2 = 0.2070
**ρ_{fin} (max/min) / [e $Å^{-3}$]**	0.78/−0.45	0.15/−0.17	0.24/−0.25
**CCDC number**	2471669	24716677	24716670

Table S4. Crystallographic details of **4c**, **5a**, and **11**-*p*-Ter.

	**4c(1.5 C_6H_{14})**	**5a**	**11**-*p*-Ter**(1.5 C_6H_6)**
**Empirical formula**	$C_{56}H_{74}N_2$	$C_{45}H_{51}N_2$	$C_{45}H_{48}N_2$
**Formula weight**	775.17	619.92	616.85
**Temperature [K]**	100.0(1)	100.0(1)	100.0(1)
**Crystal system**	monoclinic	triclinic	triclinic
**Space group**	$P2_1/c$	P-1	P-1
**a [Å]**	19.0530(7)	11.6089(6)	8.8505(2)
**b [Å]**	16.4593(6)	13.0703(5)	14.4494(6)
**c [Å]**	15.2155(5)	14.7334(7)	15.9236(7)
**α [°]**	90	98.346(4)	66.603(4)
**β [°]**	100.383(3)	111.514(5)	76.428(3)
**γ [°]**	90	112.441(4)	75.238(3)
**Volume [Å^3]**	4693.4(3)	1813.5(2)	1786.69(13)
Z	4	2	2
**ρ_{calc} [g/cm^3]**	1.097	1.135	1.147
**μ [mm^{-1}]**	0.463	0.488	0.495
**F(000)**	1696.0	671.9	664.0
**Crystal size [mm^3]**	0.244 × 0.13 × 0.06	0.12 × 0.1 × 0.05	0.25 × 0.1 × 0.05
**Radiation (λ [Å])**	Cu Kα (λ = 1.54184)	Cu Kα (λ = 1.54184)	Cu Kα (λ = 1.54184)
**2Θ range for data [°]**	7.148 to 136.502	6.84 to 147.8	6.118 to 152.458
**hkl-index ranges**	$-22 \leq h \leq 22$, $-19 \leq k \leq 19$, $-18 \leq l \leq 18$	$-12 \leq h \leq 14$, $-15 \leq k \leq 10$, $-18 \leq l \leq 18$	$-11 \leq h \leq 11$, $-17 \leq k \leq 18$, $-20 \leq l \leq 20$
**Reflections collected**	66460	13303	31512
**Independent reflections**	8590 [R_{int} = 0.0988, R_{sigma} = 0.0466]	7113 [Rint = 0.0357, Rsigma = 0.0504]	7326 [R_{int} = 0.0287, R_{sigma} = 0.0221]
**Reflections with *I* > 2*σ(I)***	5515	5458	6091
**Data/restraints/parameters**	8590/0/452	7113/18/641	7326/0/432
***GoF* on F^2**	0.987	1.044	1.029
**R_1/wR_2 [$I > 2\sigma(I)$]**	R_1 = 0.0517, wR_2 = 0.1331	R1 = 0.0383, wR2 = 0.0851	R_1 = 0.0435, wR_2 = 0.1108
**R_{ind} [all data]/wR_2**	R_1 = 0.0817, wR_2 = 0.1496	R1 = 0.0561, wR2 = 0.0933	R_1 = 0.0537, wR_2 = 0.1184
**ρ_{fin} (max/min) / [e Å^{-3}]**	0.19/−0.18	0.30/−0.25	0.28/−0.18
**CCDC number**	24716671	24716672	24716673

Table S5. Crystallographic details of **11**-*m*-Ter and [**2a**][Co(CO)$_4$].

	**11**-*m*-Ter	[**2a**][Co(CO)$_4$] **(1.5 C_6H_6)**
**Empirical formula**	$C_{45}H_{48}N_2$	$C_{58}H_{60}CoN_2O_4$
**Formula weight**	616.85	908.01
**Temperature [K]**	100.0(1)	100.0(1)
**Crystal system**	orthorhombic	triclinic
**Space group**	Pna2$_1$	P-1
**a [Å]**	33.6074(4)	13.0624(6)
**b [Å]**	10.27122(11)	14.1812(6)
**c [Å]**	10.22648(10)	14.8419(5)
**α [°]**	90	89.129(3)
**β [°]**	90	70.928(4)
**γ [°]**	90	74.900(4)
**Volume [Å^3]**	3530.07(7)	2501.32(19)
Z	4	2
**ρ_{calc} [g/cm^3]**	1.161	1.206
**μ [mm^{-1}]**	0.501	0.390
**F(000)**	1328.0	962.0
**Crystal size [mm^3]**	0.32 × 0.18 × 0.11	0.4 × 0.15 × 0.08
**Radiation (λ [Å])**	Cu Kα (λ = 1.54184)	Mo Kα (λ = 0.71073)
**2Θ range for data [°]**	5.26 to 153.18	6.34 to 58.26
**hkl-index ranges**	$-42 \le h \le 40$, $-12 \le k \le 12$, $-12 \le l \le 12$	$-17 \le h \le 17$, $-19 \le k \le 19$, $-20 \le l \le 20$
**Reflections collected**	60710	81652
**Independent reflections**	7367 [R_{int} = 0.0414, R_{sigma} = 0.0195]	13450 [R_{int} = 0.0731, R_{sigma} = 0.0454]
**Reflections with *I* > 2σ(*I*)**	7109	10641
**Data/restraints/parameters**	7367/1/432	13450/0/594
***GoF* on F^2**	1.070	1.042
**R_1/wR_2 [$I > 2\sigma(I)$]**	R_1 = 0.0310, wR_2 = 0.0751	R_1 = 0.0584, wR_2 = 0.1395
**R_{ind} [all data]/wR_2**	R_1 = 0.0330, wR_2 = 0.0772	R_1 = 0.0760, wR_2 = 0.1502
**ρ_{fin} (max/min) / [e Å^{-3}]**	0.19/−0.17	1.17/−0.49
**CCDC number**	24716674	24716675

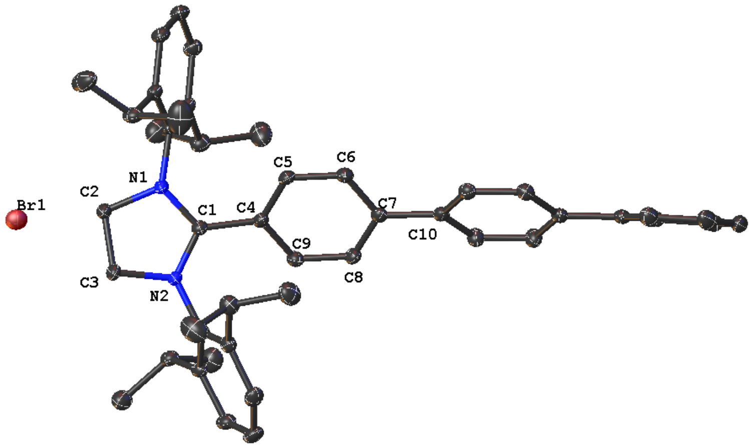

Figure S58. Solid-state molecular structure of **2a**. Hydrogen atoms were omitted for clarity. Thermal displacement ellipsoids are drawn at the 50% probability level. Selected bond lengths (Å), angles and torsions angles (°): N1–C1 1.334(2), N1–C2 1.489(2), N2–C1 1.334(2), N2–C3 1.486(2), C1–C4 1.475(2), C2–C3 1.522(2), C4–C5 1.396(2), C4–C9 1.403(2), C5–C6 1.389(2), C6–C7 1.399(2), C7–C8 1.398(2), C7–C10 1.485(2), C8–C9 1.381(2), C13–C16 1.490(2); N2–C1–N1 111.1(1), C1–N1–C2 109.8(1), C1–N2–C3 110.2(1), N1–C1–C4 126.0(1), N1–C2–C3 101.9(1); N1–C1–C4–C5 37.1(3),C6–C7–C10–C11 26.4(2), C12–C13–C16–C17 26.8(2).

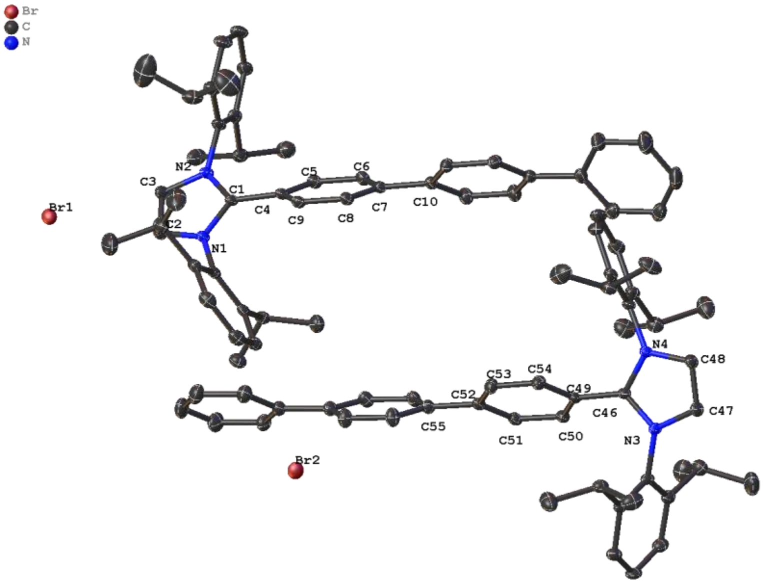

Figure S59. Solid-state molecular structure of **2b**. Hydrogen atoms were omitted for clarity. Thermal displacement ellipsoids are drawn at the 50% probability level. Selected bond lengths (Å), angles and torsion angles (°): N1–C1 1.345(2), N1–C2 1.387(2), N2–C1 1.354(2), N2–C3 1.378(2), C1–C4 1.465(2), C2–C3 1.348(2), C4–C5 1.400(2), C4–C9 1.401(2), C5–C6 1.385(2), C6–C7 1.402(2), C7–C8 1.406(2), C7–C10 1.478(2), C8–C9 1.382(2), C10–C11 1.394(2); C1–N1–C2 109.2(1), C1–N2–C3 109.2(1), N1–C1–N2 107.0(1), C3–C2–N1 107.1(1), C2–C3–N2 107.5(1), C6–C7–C10 120.9(1); C6–C7–C10–C11 35.4(2).

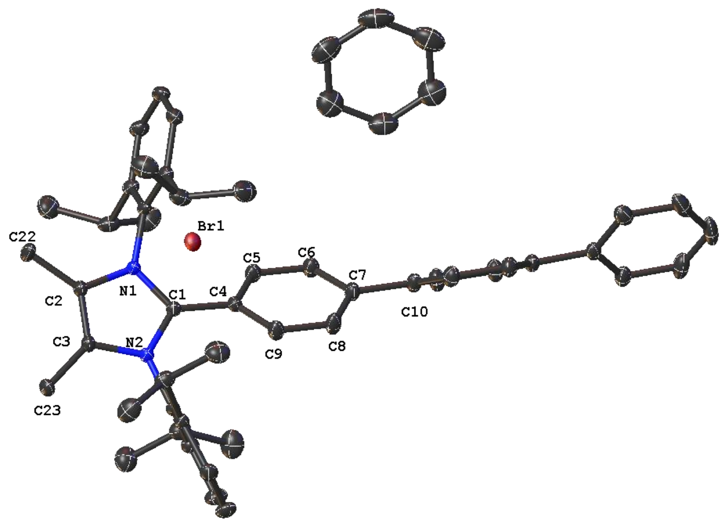

Figure S60. Solid-state molecular structure of **2c**. Hydrogen atoms were omitted for clarity. Thermal displacement ellipsoids are drawn at the 50% probability level. Selected bond lengths (Å), angles and torsion angles (°): N1–C1 1.349(1), N1–C2 1.400(1), N2–C1 1.346(1), N2–C3 1.395(1), C1–C4 1.466(2), C2–C3 1.358(2), C4–C5 1.399(2), C4–C9 1.396(2), C5–C6 1.387(2), C6–C7 1.399 (2), C7–C8 1.395(2), C7–C10 1.482(2), C8–C9 1.384(2), C10–C11 1.398(2); C1–N1–C2 109.6(1), C1–N2–C3 109.9(1), N1–C1–C4 126.6(1), N2–C1–N1 106.9(1), N2–C1–C4 126.5(1), C3–C2–N1 106.7(1), C11–C10–C7 121.7(1); C6–C7–C10–C11 36.3(2). C12–C13–C16–C17 35.2(2).

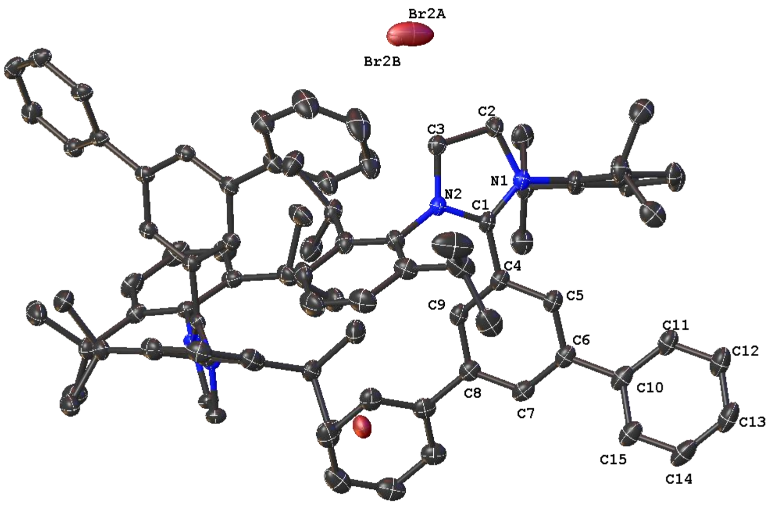

Figure S61. Solid-state molecular structure of **3a**. Hydrogen atoms were omitted for clarity. Thermal displacement ellipsoids are drawn at the 50% probability level. Selected bond lengths (Å) and angles (°): N1–C1 1.324(2), N1–C2 1.482(2), N2–C1 1.333(2), N2–C3 1.484(2), C1–C4 1.471(3), C2–C3 1.530(2), C4–C5 1.397(2), C4–C9 1.391(3), C5–C6 1.396(3), C6–C7 1.401(3), C6–C10 1.489(3), C7–C8 1.403(3), C8–C9 1.396(3), C8–C16 1.487(3), C10–C11 1.396(3); C11–C12 1.388(3); C1–N1–C2 111.1(1), C1–N2–C3 110.9(1), N1–C1–N2 111.8(2), C5–C6–C7 118.5(2), C5–C6–C10 119.7(2); N1–C1–C4–C5 37.2(3), C5–C6–C10–C11 27.1(3).

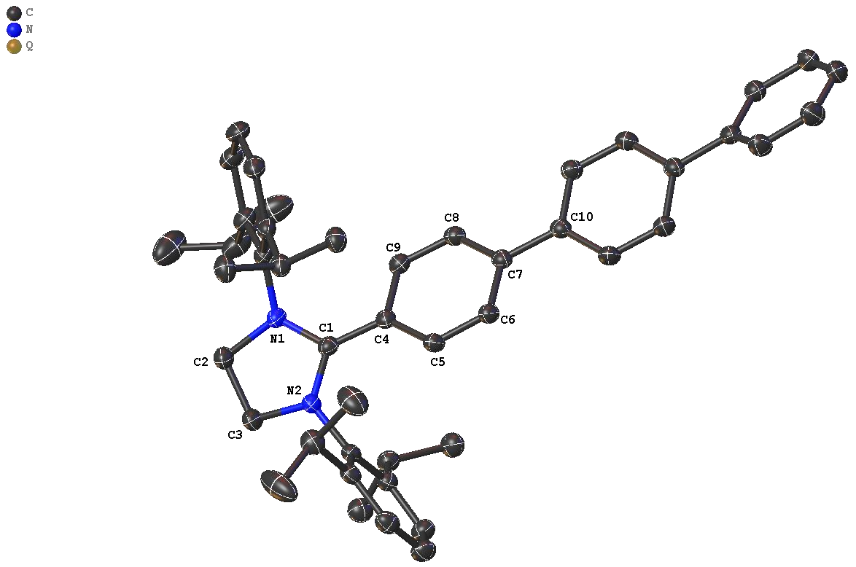

Figure S62. Solid-state molecular structure of **4a**. Hydrogen atoms were omitted for clarity. Thermal displacement ellipsoids are drawn at the 50% probability level. Selected bond lengths (Å), angles and torsion angles (°): N1–C1 1.402(1), N1–C2 1.472(1), N2–C1 1.393(1), N2–C3 1.464(1), C1–C4 1.406(2), C2–C3 1.519(2), C4–C5 1.430(2), C4–C9 1.431(2), C5–C6 1.377(2), C6–C7 1.414(2), C7–C8 1.415(2), C7–C10 1.467(2), C8–C9 1.378(2), C10–C11 1.407(2); C13–C16 1.479(1), N2–C1–N1 108.3(1), C1–N1–C2 109.3(1), C1–C4–C5 123.2(1), C11–C10–C7 121.5(1); N1–C1–C4–C5 165.4(1) [14.6(1)], C6–C7–C10–C11 8.5(1), C12–C13–C16–C17 31.0(1).

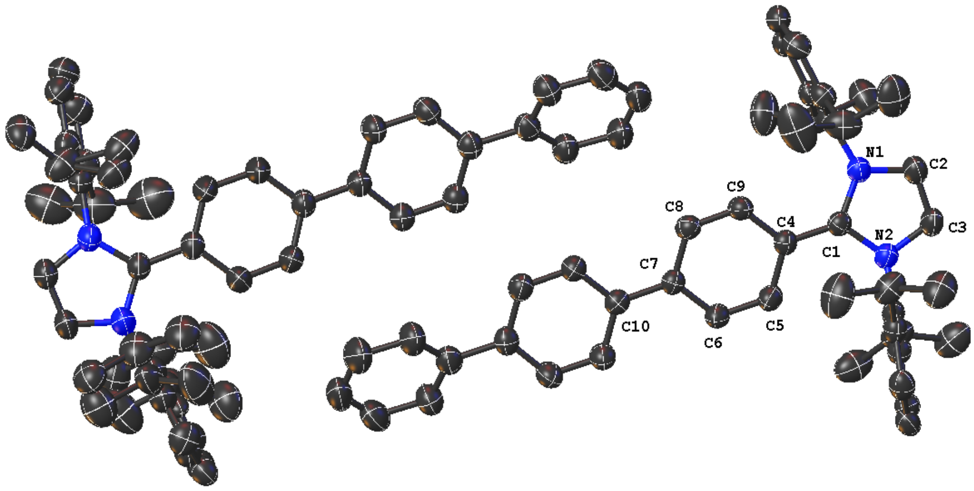

Figure S63. Solid-state molecular structure of **4b**. Hydrogen atoms were omitted for clarity. Thermal displacement ellipsoids are drawn at the 50% probability level. Selected bond lengths (Å), angles and torsion angles (°): N1–C1 1.399(3), N1–C2 1.389(3), N2–C1 1.402(3), N2–C3 1.390(3), C1–C4 1.404(3), C2–C3 1.339(4), C4–C5 1.431(3), C4–C9 1.428(3), C5–C6 1.364(4), C6–C7 1.414(3), C7–C8 1.415(3), C7–C10 1.454(3), C8–C9 1.362(4), C10–C11 1.404(4); N1–C1–N2 104.6(2), C1–C4–C5 122.3(2), C11–C10–C7 122.5(2); N1–C1–C4–C5 167.4(2) [2.6(2)], C6–C7–C10–C11 0.3(3), C12–C13–C16–C17 32.2 (4).

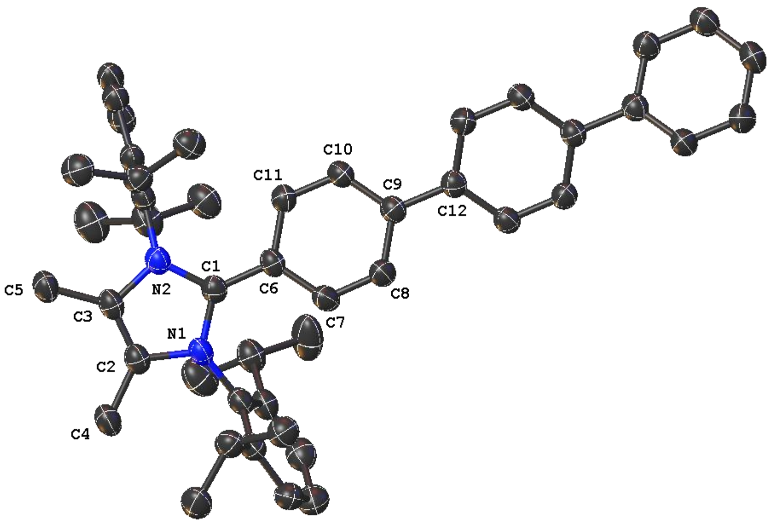

Figure S64. Solid-state molecular structure of **4c**. Hydrogen atoms were omitted for clarity. Thermal displacement ellipsoids are drawn at the 50% probability level. Selected bond lengths (Å), angles and torsion angles (°): N1–C1 1.400(2), N1–C2 1.411(2), N2–C1 1.394(2), N2–C3 1.408(2), C1–C6 1.412(3), C2–C3 1.326(3), C2–C4 1.489(3), C3–C5 1.494(3), C6–C7 1.426(3), C6–C11 1.435(3), C7–C8 1.364(3), C8–C9 1.419(3), C9–C10 1.407(3), C9–C12 1.460(3), C10–C11 1.372(3); C1–N1–C2 109.0(2), N1–C1–C6 126.6(2), C1–C6–C7 122.4(2), C13–C12–C9 122.1(2); N1–C1–C6–C7 7.3(3), C10–C9–C12–C17 13.8(3), C14–C15–C18–C19 24.6(3).

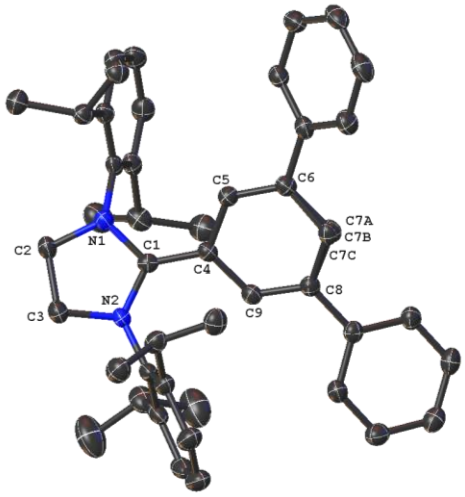

Figure S65. Solid-state molecular structure of **5a**. Hydrogen atoms were omitted for clarity. Thermal displacement ellipsoids are drawn at the 50% probability level. Selected bond lengths (Å), angles and torsion angles (°): N1–C1 1.398(2), N1–C2 1.466(2), N2–C1 1.384(2), N2–C3 1.466(2), C1–C4 1.394(2), C2–C3 1.527(2), C4–C5 1.435(2), C4–C9 1.434(2), C5–C6 1.363(2), C6–C7A 1.46(4), C6–C7B 1.49(5), C6–C7C 1.46(3), C6–C10 1.484(2), C7A–C8 1.45(5), C7B–C8 1.47(5), C7C–C8 1.50(3), C8–C9 1.368(2), C8–C16 1.484(2); N2–C1–N1 108.5(1), C4–C1–N1 124.9(1); N1–C1–C4–C5 18.2(2), C5–C6–C10–C11 39.0(2), C9–C8–C16–C21 30.3(2).

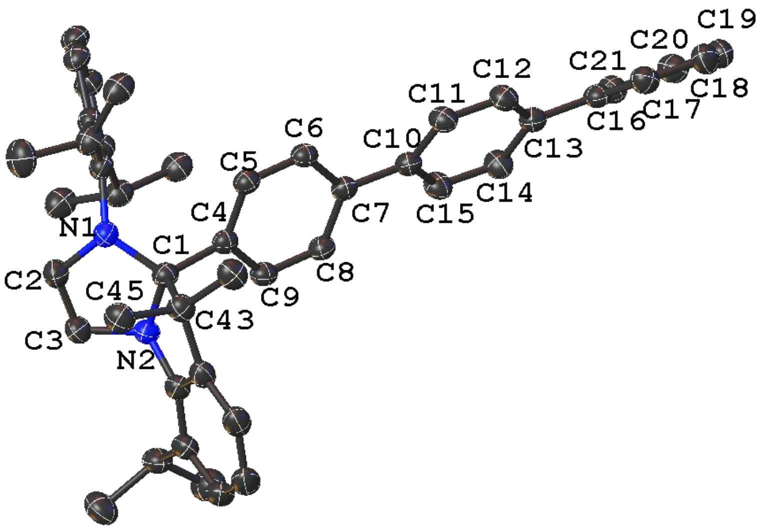

Figure S66. Solid-state molecular structure of **11**-*p*-Ter. Hydrogen atoms were omitted for clarity. Thermal displacement ellipsoids are drawn at the 50% probability level. Selected bond lengths (Å), angles and torsion angles (°): N1–C1 1.494(2), N1–C2 1.418(2), N2–C1 1.510(2), N2–C3 1.426(2), C1–C43 1.610 (2), C2–C3 1.323(2), C4–C5 1.400 (2), C4–C9 1.394(2), C5–C6 1.385(2), C6–C7 1.399(2), C7–C8 1.400(2), C7–C10 1.487(2), C8–C9 1.383(2), C10–C11 1.399(2); N1–C1–N2 103.3(1), N1–C1–C4 115.0(1), N1–C1–C43 114.0(1), C4–C1–C43 110.4(1); C6–C7–C10–C11 22.2(2), C12–C13–C16–C17 33.4(2).

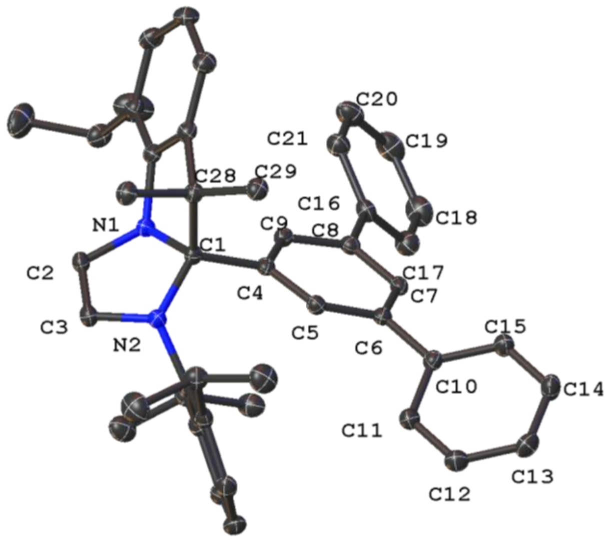

Figure S67. Solid-state molecular structure of **11**-*m*-Ter. Hydrogen atoms were omitted for clarity. Thermal displacement ellipsoids are drawn at the 50% probability level. Selected bond lengths (Å), angles and torsion angles (°): N1–C1 1.504(2), N1–C2 1.422(2), N2–C1 1.492(2), N2–C3 1.425(2), C1–C4 1.529(2), C1–C28 1.595(2), C2–C3 1.329(3), C4–C5 1.397(2), C4–C9 1.394(2), C5–C6 1.401(2), C6–C7 1.397(2), C6–C10 1.484(2), C7–C8 1.398(2), C8–C9 1.401(2), C8–C16 1.489(2); N2–C1–N1 103.5(1), N1–C1–C4 109.3(1), N1–C1–C28 102.7(1), C4–C1–C28–111.1(1); C2–N1–C1–C28 103.3(1); C7–C8–C16–C17 37.7(2), C5–C6–C10–C11 36.7(2).

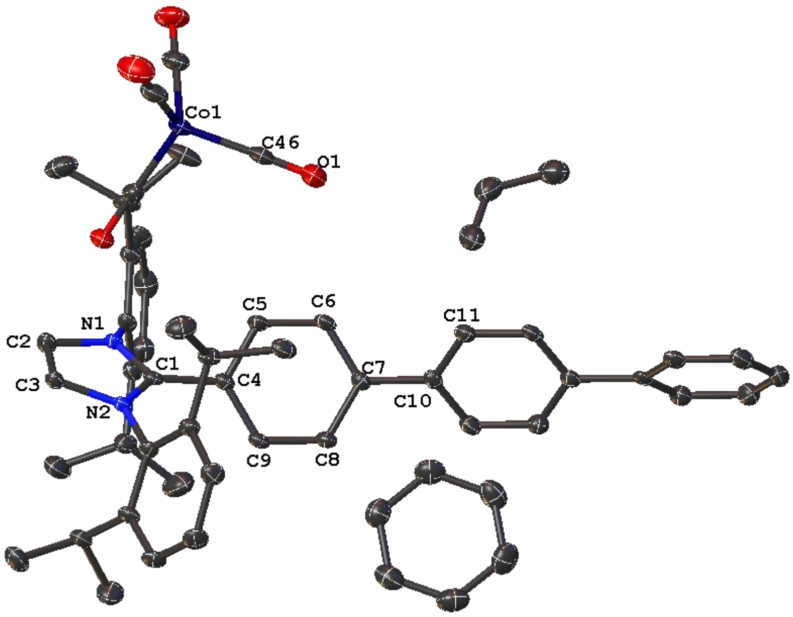

Figure S68. Solid-state molecular structure of [**2a**][Co(CO)$_4$]. Hydrogen atoms were omitted for clarity. Thermal displacement ellipsoids are drawn at the 50% probability level. Selected bond lengths (Å), angles and torsion angles (°): Co1–C46 1.775(3), O1–C46 1.154(3), N1–C1 1.333(2), N1–C2 1.481(2), N2–C1 1.327(2), N2–C3 1.484(2), C1–C4 1.474(3), C2–C3 1.538(3), C4–C5 1.401(2), C4–C9 1.400(3), C5–C6 1.383(3), C6–C7 1.400(3), C7–C8 1.406(3), C7–C10 1.488(3), C8–C9 1.386(3), C10–C11 1.399(3); O1–C46–Co1 178.7(2), N2–C1–N1 112.1(2), N1–C1–C4 123.0(2); N1–C1–C4–C5 48.7(3), C6–C7–C10–C11 41.9(3), C12–C13–C16–C17 32.5(3).

Table S6. Comparison of sc-XRD structural parameters of **2a**, **3a**, **4a**, and **5a**.

	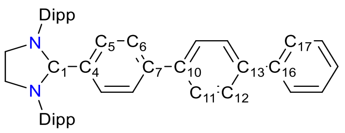		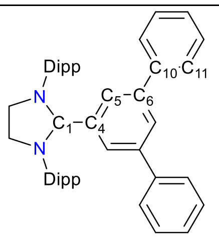	
Bond length [Å] or anlge [°] or torsion angle [°]	[(SIPr)*p*-Ter]Br (**2a**)	[(SIPr)*p*-Ter]Br (**4a**)	[(SIPr)*m*-Ter]Br (**3a**)	[(SIPr)*m*-Ter]Br (**5a**)
C1–C4	1.475(2)	1.406(2)	1.471(3)	1.394(2)
C1−N1	1.334(2)	1.402(1)	1.324(2)	1.398(2)
C1−N2	1.334(2)	1.393(1),	1.333(2)	1.384(2)
C2–C3	1.522(2)	1.519(2)	1.530(2)	1.527(2)
C4–C5	1.396(2)	1.430(2)	1.397(2)	1.435(2)
C5–C6	1.389(2)	1.377(2)	1.396(3)	1.363(2)
C6–C10	-	-	1.489(3)	1.484(2)
C7–C10	1.485(2)	1.467(2)	-	-
N–C1–N	111.1(1)	108.3(1)	111.8(2)	108.5(1)
N–C1–C4–C5	37.1(3)	165.4(1) [14.6(1)]	37.2(3)	18.2(2)
C6–C7–C10–C11	26.4(2)	8.5(1)	-	-
C12–C13–C16–C17	26.8(2)	31.0(1)	-	-
C5–C6–C10–C11	-	-	27.1(3)	39.0(2)

Computational Details

All calculations, unless stated otherwise, were performed using Orca 6.0.1 software package,[15]. Molecular structures of the cations [**2a**]$^{+}$ and [**3a**]$^{+}$, the radicals **4a** and **5a**, and the anions [**6a**]$^{-}$ and [**7a**]$^{-}$ were optimized at the PBEh-3c level of theory.[16] For this purpose, electronic states corresponding to the lowest energy doublet (**D**), closed shell singlet (**CS**), and triplet (**T**) electronic solutions were obtained using unrestricted (UKS) and restricted (RKS) Kohn-Sham formalism. All species under investigations were modeled as isolated molecules. As starting approximations, the geometries from the available experimental crystal structures (sc-XRD) were taken. Otherwise, they were modeled manually. The target convergence in geometry optimizations was set to *TightOpt*. In all calculations, we used the settings *TightSCF* and *DefGrid3*, as well as the RIJCOSX accelerating approximation.[17] The lowest energy **CS** electronic solutions were tested for the stability, which appeared to be stable. The parameters for the electronic solutions are given in Table S7. The optimized structures with only slight deviation (due to finite convergence criteria) from symmetry were additionally symmetrized. The obtained molecular structures of compounds are shown in Figures S69–S74. At the next stage of the theoretical investigation, DFT calculations were performed at the PBE0/def2-TZVP level.[18] Spin-density plots for **4a** and **5a** are shown in Figure S75 and S76, respectively. Fractional occupation weighted density (FOD)[19] calculations were performed at the PBE0/def2-TZVP (T_{el} = 10000 K) level of theory for structures with the lowest energy. Resulting N_{FOD} values are listed in Table S7. Corresponding FOD plots are shown in Figures S77–S82.

Natural bond orbital (NBO) analyses[20] were performed using NBO 7.0.10 program.[21] The wavefunctions for these calculations were taken from the PBE0/def2-TZVP single point calculations. The results are collected in Tables S8–13.

TD-DFT calculations were done at the PBE0/def2-TZVP level using TDA approximation and CPCM solution model of toluene. Selected transitions are listed in Tables S14–S15. Selected frontier molecular orbitals (FMOs) from these calculations are collected in Tables S16–S21.

Table S7. Electronic structure parameters of the calculated molecules.

Molecule	*E*(CS)[a]	*E*(D or T)[a]	<S^2>(D or T)[b]	N_{FOD}[c]
[**2a**]$^+$	0	58.5	2.053	1.19
[**3a**]$^+$	0	66.0	2.069	1.15
**4a**	n.a.	0	0.802	1.76
**5a**	n.a.	0	0.801	1.77
[**6a**]$^-$	0	20.6	2.037	2.44
[**7a**]$^-$	0	10.4	2.044	2.49

[a] Relative adiabatic energy in kcal/mol (PBEh-3c).
[b] Expectation value of the spin-squared operator.
[c] Number of “hot” electrons obtained as the result of FOD calculation (FT-PBE0/def2-TZVP *T* = 10000 K).

Table S8. Natural charges (*q*) and Wiberg bond indices (WBI) for selected atoms and bonds for [**2a**]$^+$ as obtained from NBO analysis.

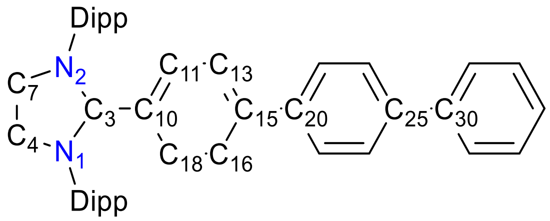

Atom	*q*	Bond	WBI
N1	−0.36	N1–C3	1.30
N2	−0.36	N1–C4	0.94
C3	0.58	N2–C3	1.30
C4	−0.23	N2–C7	0.94
C7	−0.23	C3–C10	1.07
C10	−0.17	C4–C7	1.00
C11	−0.16	C10–C11	1.35
C13	−0.20	C10–C18	1.35
C15	0.02	C11–C13	1.47
C16	−0.20	C13–C15	1.36
C18	−0.16	C15–C16	1.36
C20	−0.09	C15–C20	1.07
C25	−0.01	C16–C18	1.47
C30	−0.06	C25–C30	1.06

Table S9. Natural charges (*q*) and Wiberg bond indices (WBI) for selected atoms and bonds for [**3a**]$^+$ as obtained from NBO analysis.

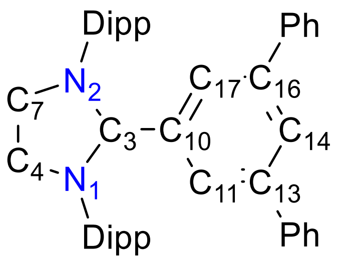

Atom	*q*	Bond	WBI
N1	−0.35	N1–C3	1.31
N2	−0.35	N1–C4	0.94
C3	0.58	N2–C3	1.31
C4	−0.23	N2–C7	0.94
C7	−0.23	C3–C10	1.06
C10	−0.15	C4–C7	1.00
C11	−0.16	C10–C11	1.37
C13	−0.03	C10–C17	1.37
C14	−0.15	C11–C13	1.39
C16	−0.03	C13–C14	1.39
C17	−0.16	C14–C16	1.39
		C16–C17	1.39

Table S10. Natural charges (*q*), spin densities (*s*), and Wiberg bond indices (WBI) for selected atoms and bonds for **4a** as obtained from NBO analysis.

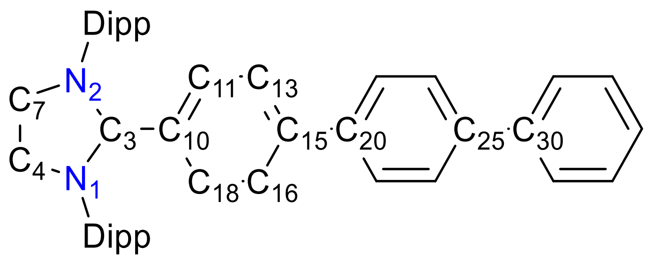

**Atom**	***q***	***s***	**Bond**	**WBI**
N1	−0.43	0.08	N1−C3	1.10
N2	−0.43	0.08	N1−C4	0.96
C3	0.40	0.35	N2−C3	1.10
C4	−0.22	−0.003	N2−C7	0.96
C7	−0.22	−0.003	C3−C10	1.30
C10	−0.19	−0.04	C4−C7	1.00
C11	−0.21	0.18	C10−C11	1.23
C13	−0.20	−0.09	C10−C18	1.23
C15	−0.09	0.24	C11−C13	1.54
C16	−0.20	−0.09	C13−C15	1.31
C18	−0.21	0.18	C15−C16	1.31
C20	−0.04	−0.04	C15−C20	1.10
C25	−0.07	0.06	C16−C18	1.54
C30	−0.04	−0.01	C25−C30	1.08

Table S11. Natural charges (*q*), spin densities (*s*), and Wiberg bond indices (WBI) for selected atoms and bonds for **5a** as obtained from NBO analysis.

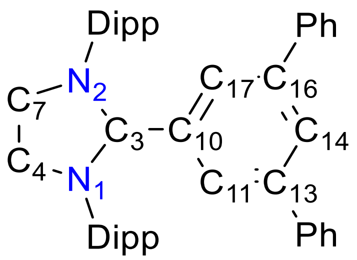

Atom	*q*	*s*	Bond	WBI
N1	−0.43	0.08	N1–C3	1.10
N2	−0.43	0.08	N1–C4	0.96
C3	0.39	0.39	N2–C3	1.10
C4	−0.22	−0.003	N2–C7	0.96
C7	−0.22	−0.003	C3–C10	1.28
C10	−0.17	−0.05	C4–C7	1.00
C11	−0.21	0.20	C10–C11	1.25
C13	−0.05	−0.09	C10–C17	1.25
C14	−0.24	0.26	C11–C13	1.45
C16	−0.05	−0.09	C13–C14	1.33
C17	−0.21	0.20	C14–C16	1.33
			C16–C17	1.45

Table S12. Natural charges (*q*) and Wiberg bond indices (WBI) for selected atoms and bonds for [**6a**]$^{-}$ as obtained from NBO analysis.

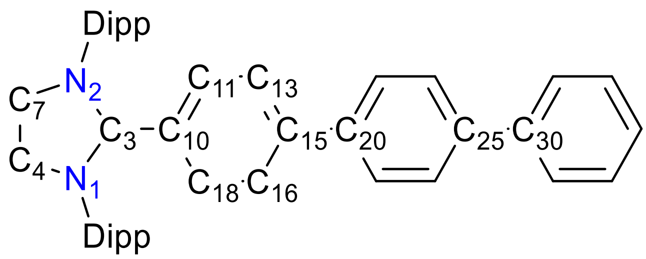

Atom	*q*	Bond	WBI
N1	−0.46	N1–C3	0.99
N2	−0.46	N1–C4	0.98
C3	0.27	N2–C3	0.99
C4	−0.22	N2–C7	0.98
C7	−0.22	C3–C10	1.50
C10	−0.19	C4–C7	1.00
C11	−0.28	C10–C11	1.13
C13	−0.18	C10–C18	1.13
C15	−0.21	C11–C13	1.65
C16	−0.18	C13–C15	1.21
C18	−0.28	C15–C16	1.21
C20	−0.02	C15–C20	1.24
C25	−0.16	C16–C18	1.65
C30	−0.02	C25–C30	1.11

Table S13. Natural charges (*q*) and Wiberg bond indices (WBI) for selected atoms and bonds for [**7a**]$^{-}$ as obtained from NBO analysis.

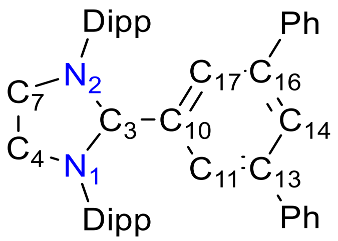

Atom	*q*	Bond	WBI
N1	−0.47	N1–C3	0.96
N2	−0.46	N1–C4	0.98
C3	0.18	N2–C3	0.97
C4	−0.22	N2–C7	0.98
C7	−0.21	C3–C10	1.48
C10	−0.16	C4–C7	1.00
C11	−0.30	C10–C11	1.15
C13	−0.05	C10–C17	1.15
C14	−0.40	C11–C13	1.48
C16	−0.05	C13–C14	1.30
C17	−0.27	C14–C16	1.37
		C16–C17	1.51

Table S14. Selected transitions (vertical energy differences) in the TD-DFT calculation of **4a**. Wavelengths λ in nm, oscillator strengths f via transition electric dipole moments and assignments are listed.

λ, nm	f	Assignment
616	0.45	91 % αHOMO → αLUMO
369	0.62	58 % βHOMO → βLUMO, 10 % αHOMO−1 → αLUMO
327	0.28	19 % αHOMO−1 → αLUMO,13 % βHOMO → βLUMO+1, 12 % βHOMO−2 → βLUMO, 11 % βHOMO → βLUMO, 11 % αHOMO → αLUMO+8

Table S15. Selected transitions transitions (vertical energy differences) in TD-DFT calculation of **5a**. Wavelengths λ in nm, oscillator strengths f via transition electric dipole moments and assignments are listed.

λ, nm	f	Assignment
802	$2x10^{-4}$	97 % αHOMO → αLUMO
557	0.08	78 % αHOMO → αLUMO+1, 12 % αHOMO → αLUMO+2
288	0.10	80 % βHOMO−2 → βLUMO
259	0.32	46 % αHOMO−1 → αLUMO, 37 % βHOMO → βLUMO+1

Table S16. Selected molecular orbitals (isosurfaces 0.05 a.u.) and respective energies (eV) in PBE0/def2-TZVP calculation for [**2a**]$^{+}$.

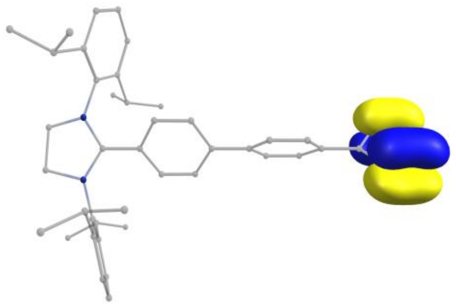 HOMO−1 (−8.9066)	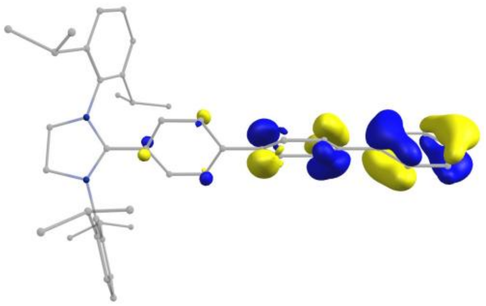 HOMO (−8.3459)
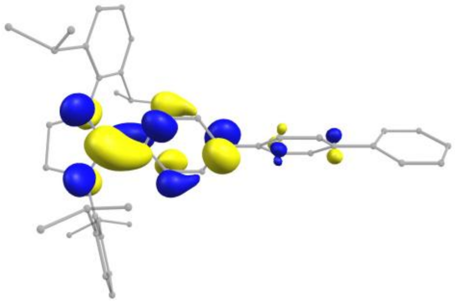 LUMO (−4.6475)	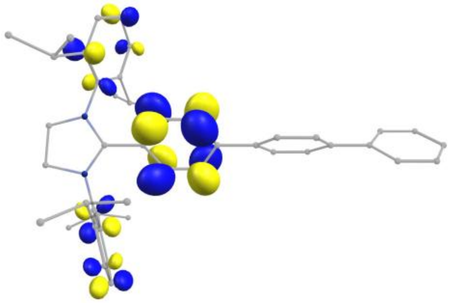 LUMO+1 (−3.3700)

Table S17. Selected molecular orbitals (isosurfaces 0.05 a.u.) and respective energies (eV) in PBE0/def2-TZVP calculation for [**3a**]$^{+}$.

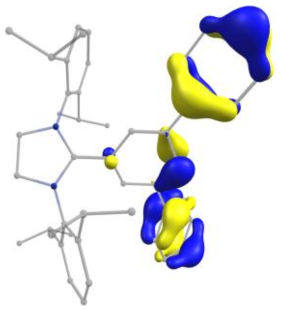 HOMO−1 (−9.2182)	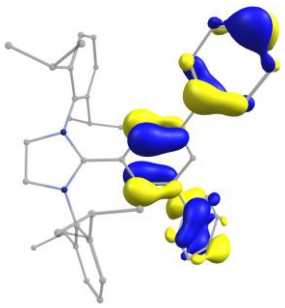 HOMO (−9.0367)
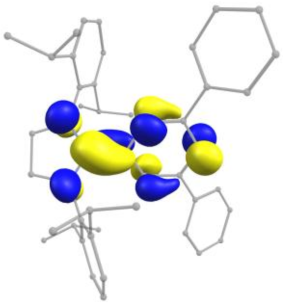 LUMO (−4.5872)	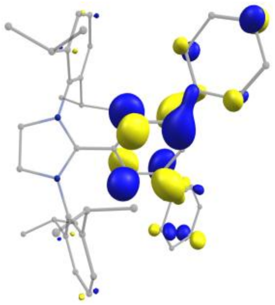 LUMO+1 (−3.7011)

Table S18. Selected molecular orbitals (isosurfaces 0.05 a.u.) and respective energies (eV) in PBE0/def2-TZVP calculation for **4a**.

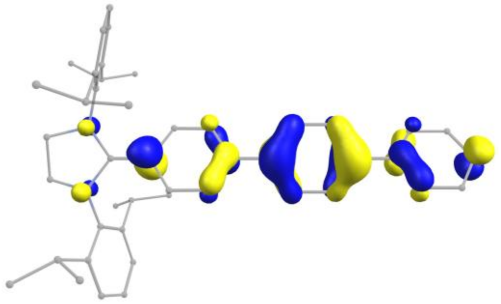 αHOMO−1 (−6.0949)	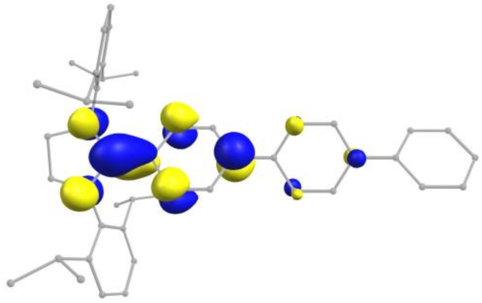 αHOMO (−3.4385)
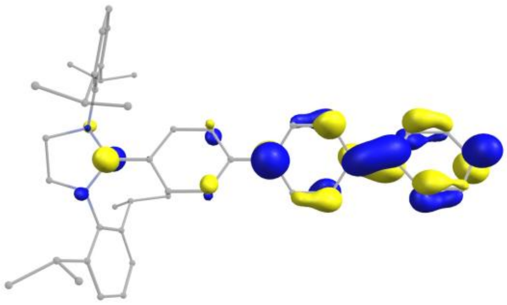 αLUMO (−0.7620)	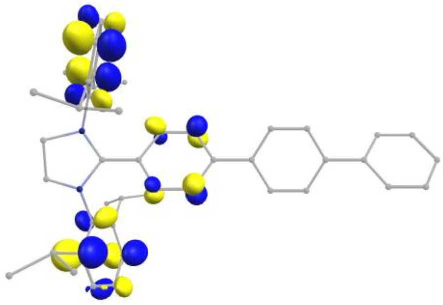 αLUMO+1 (−0.3752)
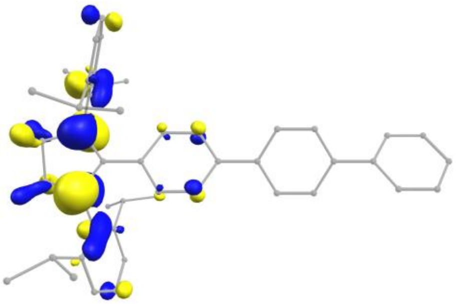 βHOMO−1 (−6.5950)	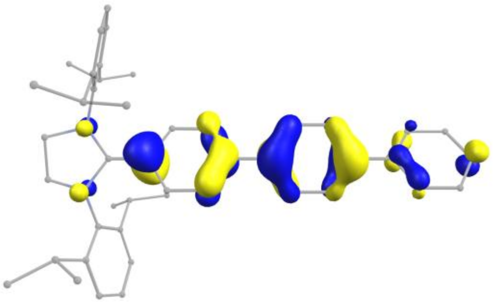 βHOMO (−5.9556)
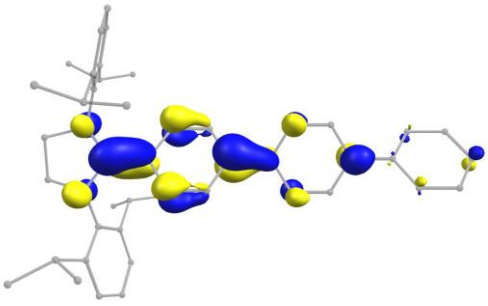 βLUMO (−1.4981)	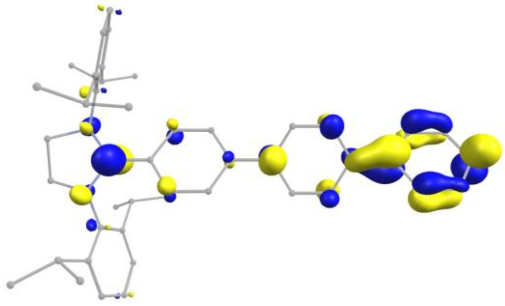 βLUMO+1 (−0.5003)

Table S19. Frontier molecular orbitals (isosurfaces 0.05 a.u.) and respective energies (eV) in PBE0/def2-TZVP calculation for **5a**.

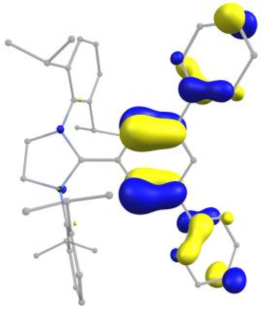 αHOMO−1 (−6.3221)	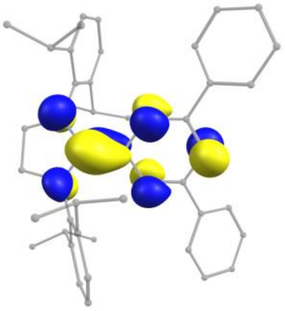 αHOMO (−3.4264)
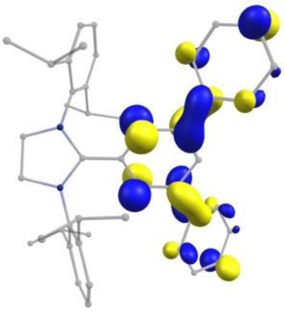 αLUMO (−0.8431)	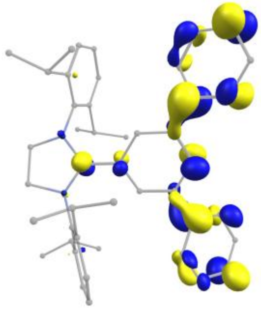 αLUMO+1 (−0.3611)
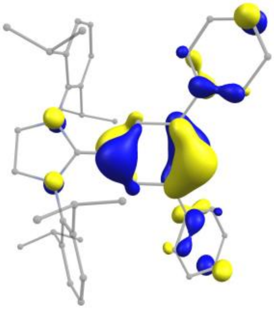 βHOMO−1 (−6.3229)	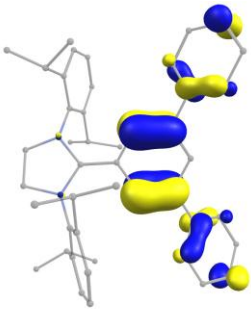 βHOMO (−6.1019)
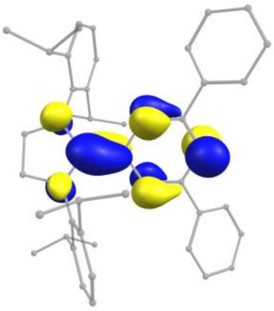 βLUMO (−1.1365)	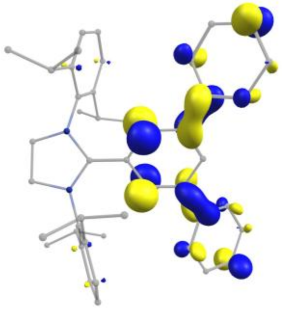 βLUMO+1 (−0.7271)

Table S20. Selected molecular orbitals (isosurfaces 0.05 a.u.) and respective energies (eV) in PBE0/def2-TZVP calculation for [**6a**]$^{-}$.

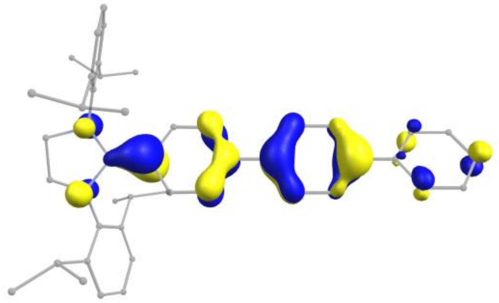 HOMO−1 (−4.2589) 4	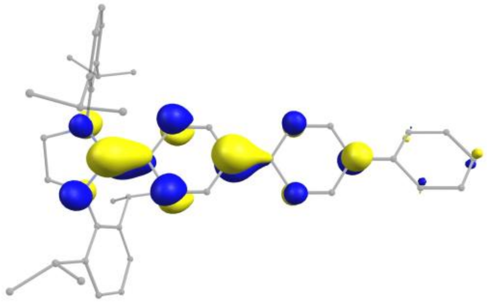 HOMO (−1.6607)
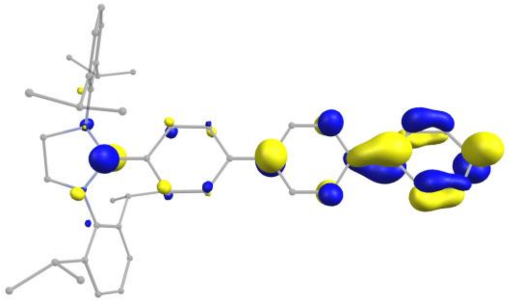 LUMO (0.4992)	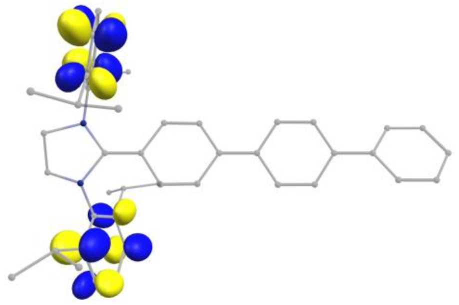 LUMO+1 (0.7763)

Table S21. Selected molecular orbitals (isosurfaces 0.05 a.u.) and respective energies (eV) in PBE0/def2-TZVP calculation for [**7a**]$^{-}$.

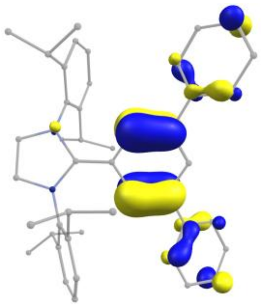 HOMO−1 (−4.5285)	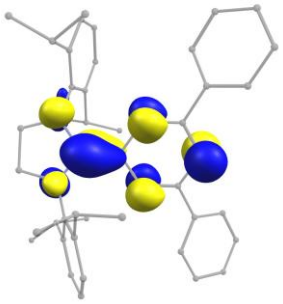 HOMO (−1.2998)
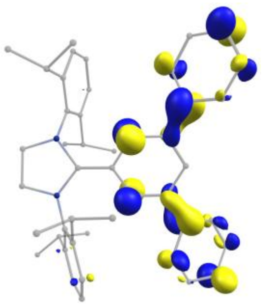 LUMO (0.6891)	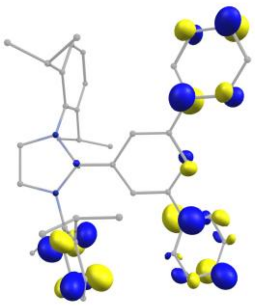 LUMO+1 (0.9832)

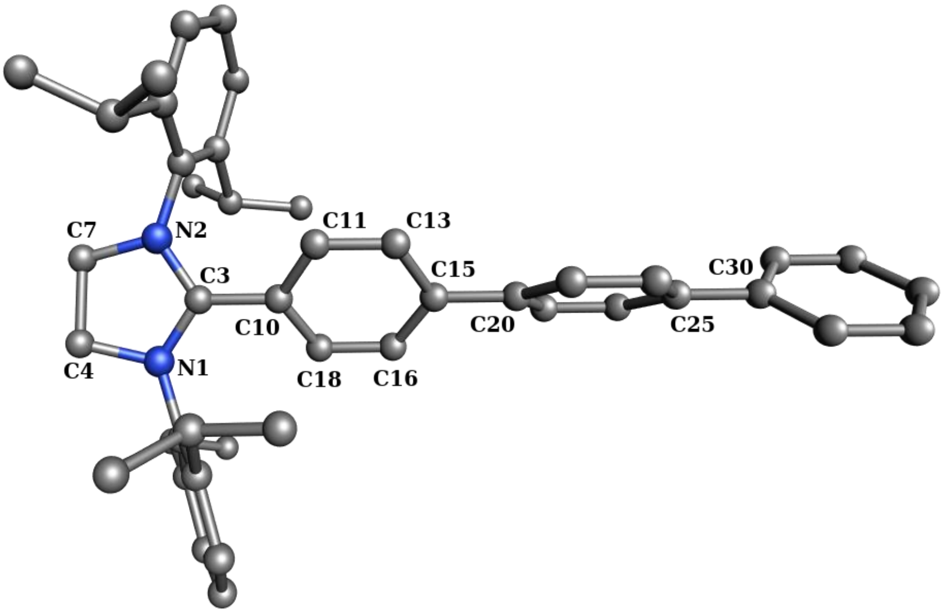

Figure S69. Optimized molecular structure of free (**2a**)$^+$ (PBEh-3c, RKS-Singlet, C2 symmetry). Hydrogen atoms are omitted for clarity. Internal working numeration of selected atoms is shown. Selected equilibrium parameters (Å, degrees) are: r(C3–N1)=1.326, r(C3–N2)=1.326, r(C4–N1)=1.462, r(C7–N2)=1.462, r(C7–C4)=1.525, r(C10–C3)=1.456, r(C11–C10)=1.395, r(C13–C11)=1.379, r(C15–C13)=1.397, r(C16–C15)=1.397, r(C18–C10)=1.395, r(C18–C16)=1.379, r(C20–C15)=1.468, r(C30–C25)=1.473, a(N1–C3–N2)=111.3, a(C3–N1–C4)=110.6, a(N1–C3–C10)=124.4, a(C3–N2–C7)=110.6, a(N2–C3–C10)=124.4, a(N1–C4–C7)=102.4, a(N2–C7–C4)=102.4, a(C3–C10–C11)=120.5, a(C3–C10–C18)=120.5, a(C10–C11–C13)=120.3, a(C11–C10–C18)=119.0, a(C11–C13–C15)=121.2, a(C13–C15–C16)=117.9, a(C13–C15–C20)=121.0, a(C15–C16–C18)=121.2, a(C16–C15–C20)=121.0, a(C10–C18–C16)=120.3, t(C4–N1–C3–N2)= –5.8, t(N1–C3–N2–C7)= –5.8, t(C3–N1–C4–C7)=14.0, t(C10–C3–N1–C4)=174.2, t(N1–C3–C10–C11)=38.7, t(N1–C3–C10–C18)= –141.3, t(C3–N2–C7–C4)=14.0, t(C10–C3–N2–C7)=174.2, t(N2–C3–C10–C11)= –141.3, t(N2–C3–C10–C18)=38.7, t(N1–C4–C7–N2)= –15.9, t(C3–C10–C11–C13)=179.7, t(C3–C10–C18–C16)=179.7, t(C10–C11–C13–C15)=0.7, t(C18–C10–C11–C13)= –0.3, t(C11–C10–C18–C16)= –0.3, t(C11–C13–C15–C16)= –0.3, t(C11–C13–C15–C20)=179.7, t(C13–C15–C16–C18)= –0.3, t(C15–C16–C18–C10)=0.7, t(C20–C15–C16–C18)=179.7, o(C20–C15<C13/C16)=0.0.

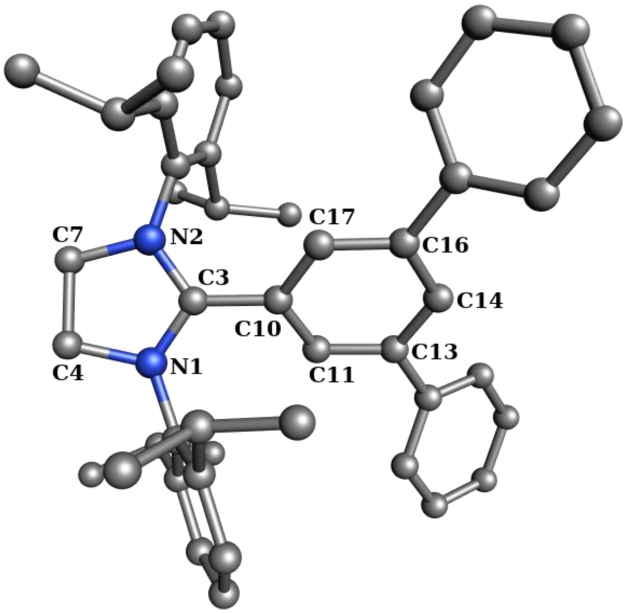

Figure S70. Optimized molecular structure of free (**3a**)$^+$ (PBEh-3c, RKS-Singlet, C2 symmetry). Hydrogen atoms are omitted for clarity. Internal working numeration of selected atoms is shown. Selected equilibrium parameters (Å, degrees) are: r(C3–N1)=1.324, r(C3–N2)=1.324, r(C4–N1)=1.463, r(C7–N2)=1.463, r(C7–C4)=1.527, r(C10–C3)=1.458, r(C11–C10)=1.389, r(C13–C11)=1.389, r(C14–C13)=1.393, r(C16–C14)=1.393, r(C17–C10)=1.389, r(C17–C16)=1.389, r(C30-C16)=1.474 A, a(N1–C3–N2)=111.6, a(C3–N1–C4)=110.5, a(N1–C3–C10)=124.2, a(C3–N2–C7)=110.5, a(N2–C3–C10)=124.2, a(N1–C4–C7)=102.5, a(N2–C7–C4)=102.5, a(C3–C10–C11)=119.8, a(C3–C10–C17)=119.8, a(C10–C11–C13)=120.3, a(C11–C10–C17)=120.4, a(C11–C13–C14)=118.5, a(C13–C14–C16)=121.9, a(C14–C16–C17)=118.5, a(C10–C17–C16)=120.3, t(C4–N1–C3–N2)= –5.5, t(N1–C3–N2–C7)= –5.5, t(C3–N1–C4–C7)=13.3, t(C10–C3–N1–C4)=174.5, t(N1–C3–C10–C11)=39.5, t(N1–C3–C10–C17)= –140.5, t(C3–N2–C7–C4)=13.3, t(C10–C3–N2–C7)=174.5, t(N2–C3–C10–C11)= –140.5, t(N2–C3–C10–C17)=39.5, t(N1–C4–C7–N2)= –15.1, t(C3–C10–C11–C13)= –179.6, t(C3–C10–C17–C16)= –179.6, t(C10–C11–C13–C14)= –0.8, t(C17–C10–C11–C13)=0.4, t(C11–C10–C17–C16)=0.4, t(C11–C13–C14–C16)=0.4, t(C13–C14–C16–C17)=0.4, t(C14–C16–C17–C10)= –0.8.

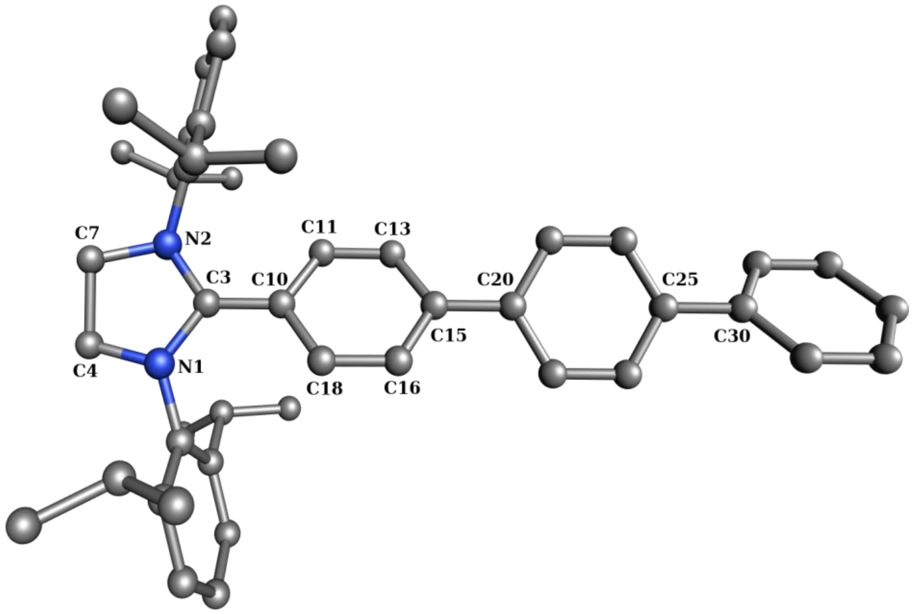

Figure S71. Optimized molecular structure of **4a** (PBEh-3c, UKS-Doublet, C_2 symmetry). Hydrogen atoms are omitted for clarity. Internal working numeration of selected atoms is shown. Selected equilibrium parameters (Å, degrees) are: r(C3–N1)=1.387, r(C3–N2)=1.387, r(C4–N1)=1.453, r(C7–N2)=1.453, r(C7–C4)=1.515, r(C10–C3)=1.401, r(C11–C10)=1.424, r(C13–C11)=1.371, r(C15–C13)=1.405, r(C16–C15)=1.405, r(C18–C10)=1.424, r(C18–C16)=1.371, r(C20–C15)=1.462, r(C30–C25)=1.473, a(N1–C3–N2)=107.7, a(C3–N1–C4)=109.8, a(N1–C3–C10)=126.1, a(C3–N2–C7)=109.8, a(N2–C3–C10)=126.1, a(N1–C4–C7)=102.4, a(N2–C7–C4)=102.4, a(C3–C10–C11)=122.1, a(C3–C10–C18)=122.1, a(C10–C11–C13)=121.6, a(C11–C10–C18)=115.8, a(C11–C13–C15)=122.3, a(C13–C15–C16)=116.4, a(C13–C15–C20)=121.8, a(C15–C16–C18)=122.3, a(C16–C15–C20)=121.8, a(C10–C18–C16)=121.6, t(C4–N1–C3–N2)=9.5, t(N1–C3–N2–C7)=9.5, t(C3–N1–C4–C7)= −23.2, t(C10–C3–N1–C4)= −170.5, t(N1–C3–C10–C11)=161.6, t(N1–C3–C10–C18)= −18.4, t(C3–N2–C7–C4)= −23.2, t(C10–C3–N2–C7)= –170.5, t(N2–C3–C10–C11)= −18.4, t(N2–C3–C10–C18)=161.6, t(N1–C4–C7–N2)=27.1, t(C3–C10–C11–C13)= −180.0, t(C3–C10–C18–C16)= −180.0, t(C10–C11–C13–C15)=−0.0, t(C18–C10–C11–C13)=0.0, t(C11–C10–C18–C16)=0.0, t(C11–C13–C15–C16)=0.0, t(C11–C13–C15–C20)= −180.0, t(C13–C15–C16–C18)=0.0, t(C15–C16–C18–C10)=0.0, t(C20−C15−C16−C18)= −180.0, o(C20–C15<C13/C16)=0.0.

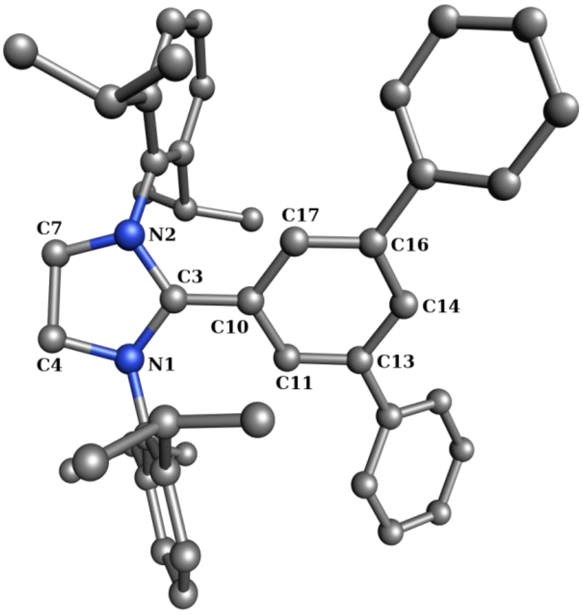

Figure S72. Optimized molecular structure of **5a** (PBEh-3c, UKS-Doublet, C_2 symmetry). Hydrogen atoms are omitted for clarity. Internal working numeration of selected atoms is shown. Selected equilibrium parameters (Å, degrees) are: r(C3–N1)=1.389, r(C3–N2)=1.389, r(C4–N1)=1.453, r(C7–N2)=1.453, r(C7–C4)=1.517, r(C10–C3)=1.404, r(C11–C10)=1.417, r(C13–C11)=1.382, r(C14–C13)=1.399, r(C16–C14)=1.399, r(C17–C10)=1.417, r(C17–C16)=1.382, r(C19-C13)=1.476 A, a(N1–C3–N2)=108.1, a(C3–N1–C4)=109.5, a(N1–C3–C10)=125.9, a(C3–N2–C7)=109.5, a(N2–C3–C10)=125.9, a(N1–C4–C7)=102.5, a(N2–C7–C4)=102.5, a(C3–C10–C11)=121.6, a(C3–C10–C17)=121.6, a(C10–C11–C13)=121.8, a(C11–C10–C17)=116.8, a(C11–C13–C14)=119.8, a(C13–C14–C16)=119.9, a(C14–C16–C17)=119.8, a(C10–C17–C16)=121.8, t(C4–N1–C3–N2)= –9.5, t(N1–C3–N2–C7)= –9.5, t(C3–N1–C4–C7)=23.4, t(C10–C3–N1–C4)=170.5, t(N1–C3–C10–C11)=19.6, t(N1–C3–C10–C17)= –160.4, t(C3–N2–C7–C4)=23.4, t(C10–C3–N2–C7)=170.5, t(N2–C3–C10–C11)= –160.4, t(N2–C3–C10–C17)=19.6, t(N1–C4–C7–N2)= –27.5, t(C3–C10–C11–C13)= –179.4, t(C3–C10–C17–C16)= –179.4, t(C10–C11–C13–C14)= –1.3, t(C17–C10–C11–C13)=0.6, t(C11–C10–C17–C16)=0.6, t(C11–C13–C14–C16)=0.6, t(C13–C14–C16–C17)=0.6, t(C14–C16–C17–C10)= –1.3.

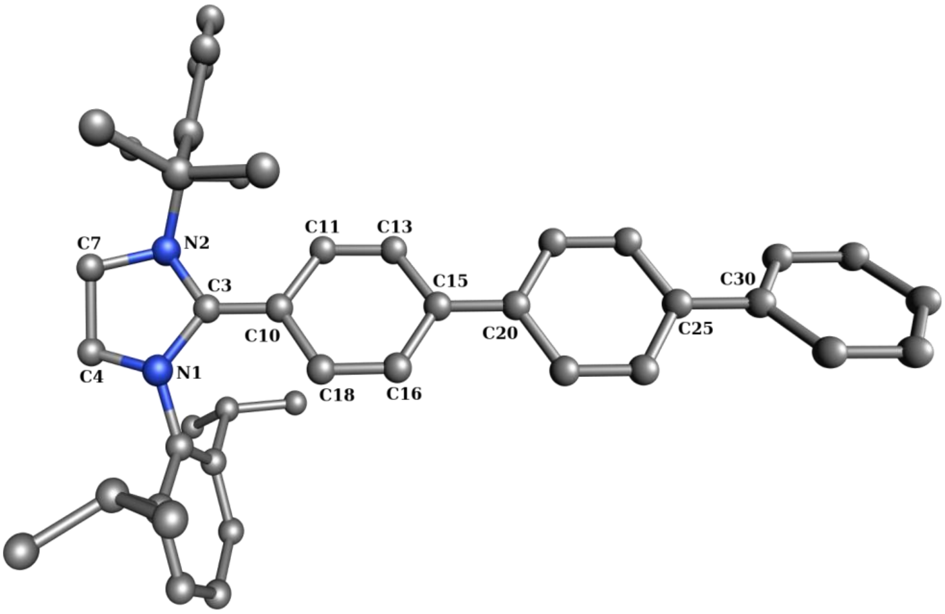

Figure S73. Optimized molecular structure of free [**6a**]$^{-}$ (PBEh-3c, RKS-Singlet, C_2 symmetry). Hydrogen atoms are omitted for clarity. Internal working numeration of selected atoms is shown. Selected equilibrium parameters (Å, degrees) are: r(C3–N1)=1.424, r(C3–N2)=1.424, r(C4–N1)=1.449, r(C7–N2)=1.449, r(C7–C4)=1.512, r(C10–C3)=1.362, r(C11–C10)=1.453, r(C13–C11)=1.359, r(C15–C13)=1.424, r(C16–C15)=1.424, r(C18–C10)=1.453, r(C18–C16)=1.359, r(C20–C15)=1.425, r(C30–C25)=1.458, a(N1–C3–N2)=106.0, a(C3–N1–C4)=109.3, a(N1–C3–C10)=127.0, a(C3–N2–C7)=109.3, a(N2–C3–C10)=127.0, a(N1–C4–C7)=102.5, a(N2–C7–C4)=102.5, a(C3–C10–C11)=123.4, a(C3–C10–C18)=123.4, a(C10–C11–C13)=122.5, a(C11–C10–C18)=113.2, a(C11–C13–C15)=124.1, a(C13–C15–C16)=113.6, a(C13–C15–C20)=123.2, a(C15–C16–C18)=124.1, a(C16–C15–C20)=123.2, a(C10–C18–C16)=122.5, t(C4–N1–C3–N2)=10.8, t(N1–C3–N2–C7)=10.8, t(C3–N1–C4–C7)= –26.8, t(C10–C3–N1–C4)= –169.2, t(N1–C3–C10–C11)=170.1, t(N1–C3–C10–C18)= –9.9, t(C3–N2–C7–C4)= –26.8, t(C10–C3–N2–C7)= –169.2, t(N2–C3–C10–C11)= –9.9, t(N2–C3–C10–C18)=170.1, t(N1–C4–C7–N2)=31.7, t(C3–C10–C11–C13)= –179.8, t(C3–C10–C18–C16)= –179.8, t(C10–C11–C13–C15)= –0.3, t(C18–C10–C11–C13)=0.2, t(C11–C10–C18–C16)=0.2, t(C11–C13–C15–C16)=0.2, t(C11–C13–C15–C20)= –179.8, t(C13–C15–C16–C18)=0.2, t(C15–C16–C18–C10)= –0.3, t(C20–C15–C16–C18)= –180.0, o(C20–C15<C13/C16)=0.0.

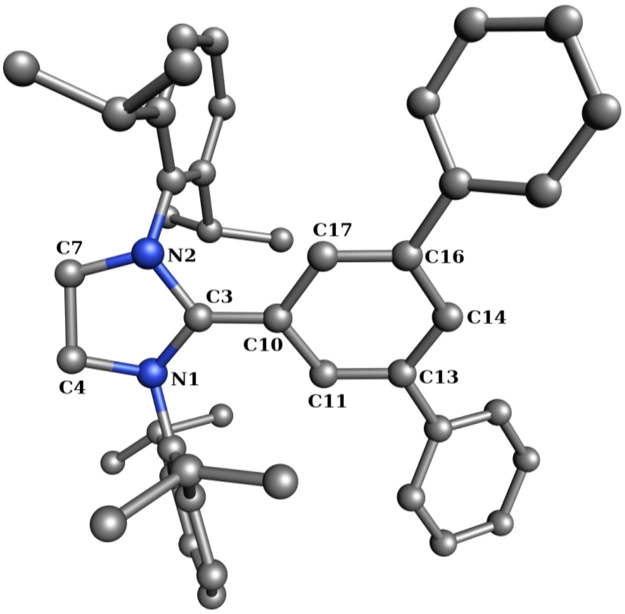

Figure S74. Optimized molecular structure of free [**7a**]$^{-}$ (PBEh-3c, RKS-Singlet, C_1 symmetry). Hydrogen atoms are omitted for clarity. Internal working numeration of selected atoms is shown. Selected equilibrium parameters (Å, degrees) are: r(C3–N1)=1.438, r(C3–N2)=1.432, r(C4–N1)=1.450, r(C7–N2)=1.449, r(C7–C4)=1.517, r(C10–C3)=1.364, r(C11–C10)=1.447, r(C13–C11)=1.379, r(C14–C13)=1.403, r(C16–C14)=1.408, r(C17–C10)=1.446, r(C17–C16)=1.373, a(N1–C3–N2)=106.2, a(C3–N1–C4)=106.9, a(N1–C3–C10)=128.2, a(C3–N2–C7)=109.4, a(N2–C3–C10)=125.4, a(N1–C4–C7)=102.6, a(N2–C7–C4)=103.1, a(C3–C10–C11)=124.1, a(C3–C10–C17)=122.1, a(C10–C11–C13)=122.6, a(C11–C10–C17)=113.7, a(C11–C13–C14)=121.7, a(C13–C14–C16)=117.5, a(C14–C16–C17)=121.6, a(C10–C17–C16)=122.8, t(C4–N1–C3–N2)= –22.8, t(N1–C3–N2–C7)=1.6, t(C3–N1–C4–C7)=33.8, t(C10–C3–N1–C4)=162.0, t(N1–C3–C10–C11)=7.8, t(N1–C3–C10–C17)= –173.7, t(C3–N2–C7–C4)=18.9, t(C10–C3–N2–C7)=176.9, t(N2–C3–C10–C11)= –166.6, t(N2–C3–C10–C17)=11.9, t(N1–C4–C7–N2)= –31.7, t(C3–C10–C11–C13)= –179.7, t(C3–C10–C17–C16)= –179.9, t(C10–C11–C13–C14)= –0.8, t(C17–C10–C11–C13)=1.7, t(C11–C10–C17–C16)= –1.3, t(C11–C13–C14–C16)= –0.7, t(C13–C14–C16–C17)=1.1, t(C14–C16–C17–C10)=0.0.

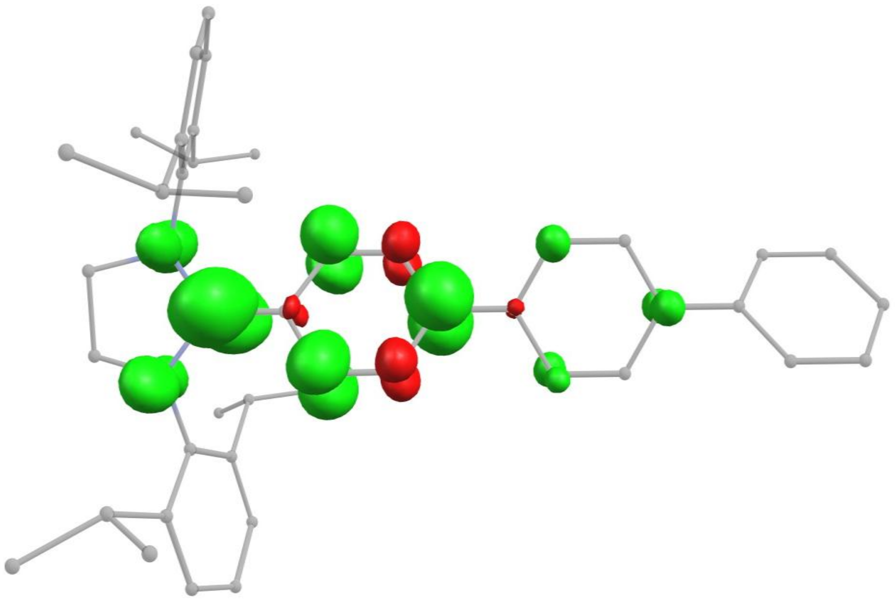

Figure S75. Spin density (isosurfaces ±0.005 a.e.) of **4a** in PBE0/def2-TZVP calculation. Hydrogen atoms are omitted for clarity.

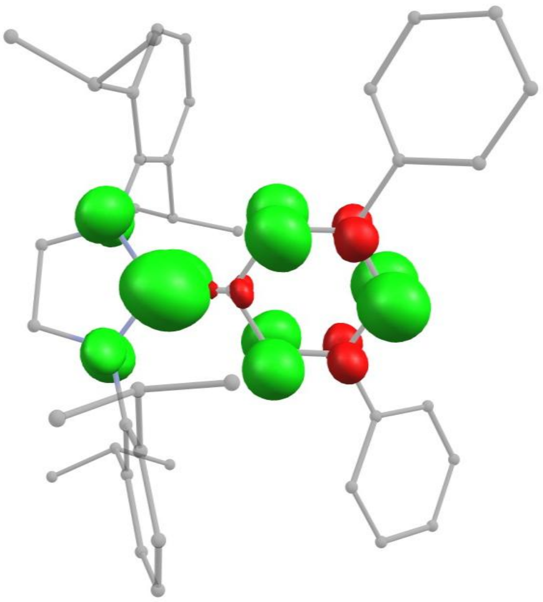

Figure S76. Spin density (isosurfaces ±0.005 a.e.) of **5a** in PBE0/def2-TZVP calculation. Hydrogen atoms are omitted for clarity.

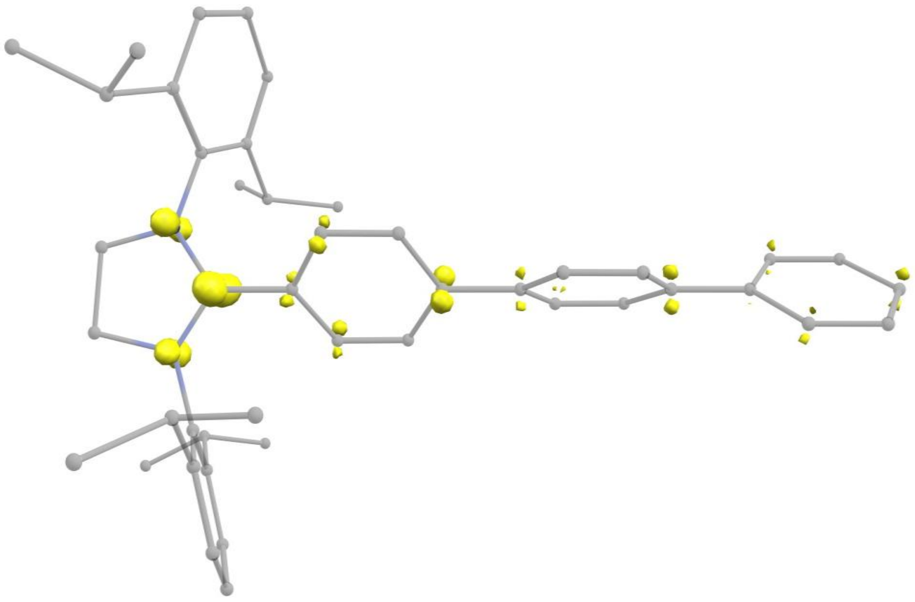

Figure S77. FOD plot (isosurfaces 0.005 a.e. in yellow) of [**2a**]$^{+}$. Hydrogen atoms are omitted for clarity.

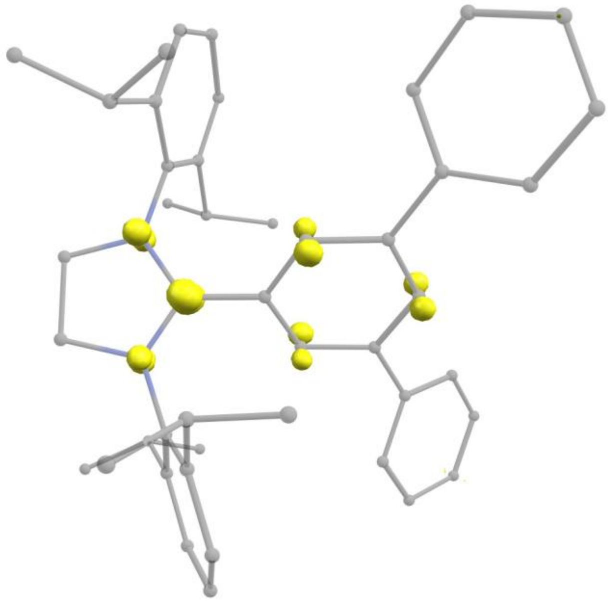

Figure S78. FOD plot (isosurfaces 0.005 a.e. in yellow) of [**3a**]$^{+}$. Hydrogen atoms are omitted for clarity.

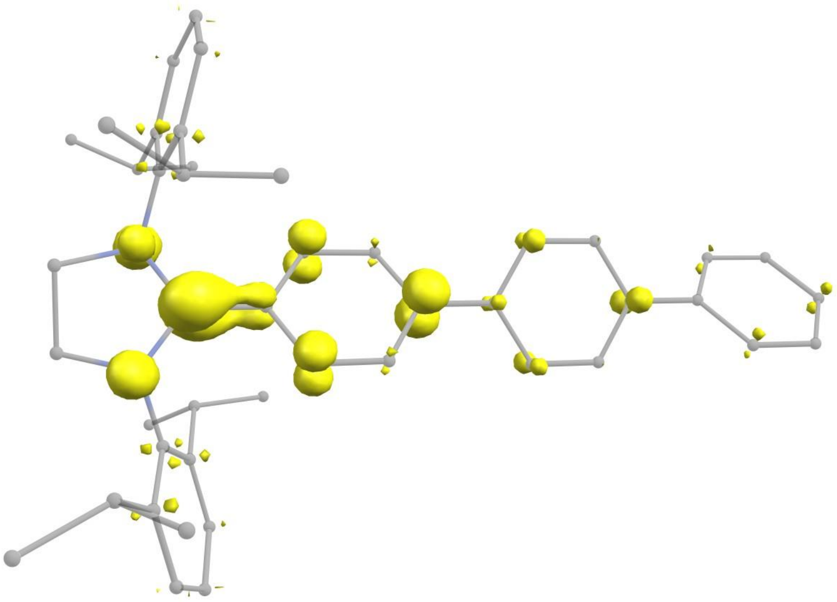

Figure S79. FOD plot (isosurfaces 0.005 a.e. in yellow) of **4a**. Hydrogen atoms are omitted for clarity.

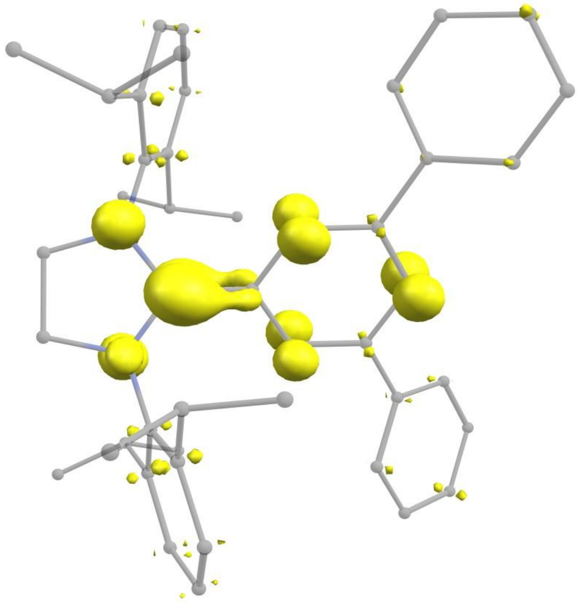

Figure S80. FOD plot (isosurfaces 0.005 a.e. in yellow) of **5a**. Hydrogen atoms are omitted for clarity.

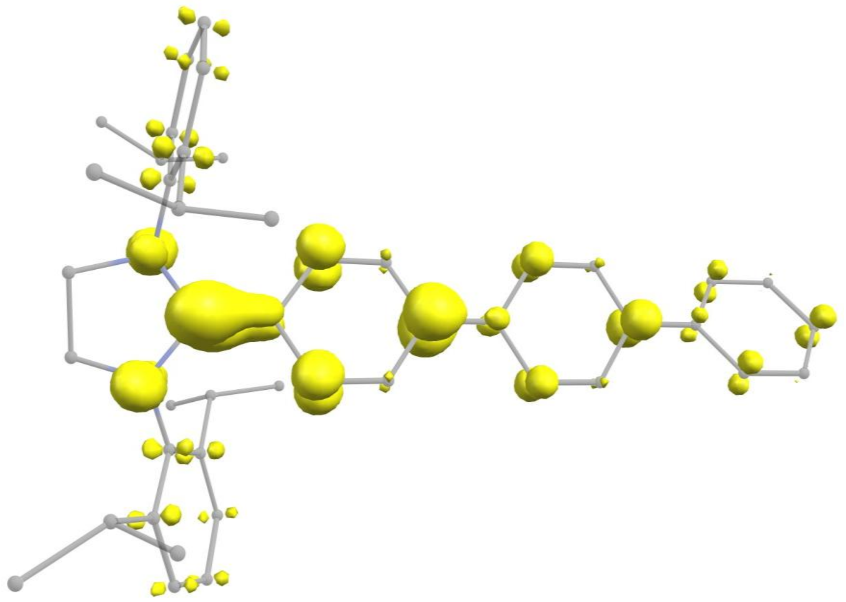

Figure S81. FOD plot (isosurfaces 0.005 a.e. in yellow) of [**6a**]$^{-}$. Hydrogen atoms are omitted for clarity.

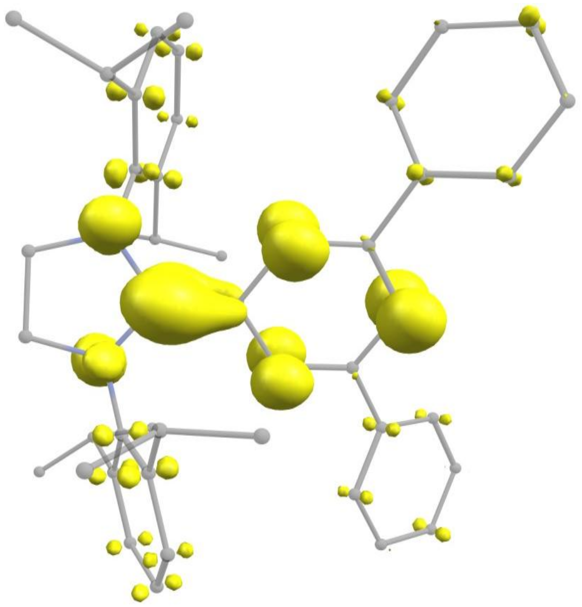

Figure S82. FOD plot (isosurfaces 0.005 a.e. in yellow) of]**7a**]$^{-}$. Hydrogen atoms are omitted for clarity.

References

[1] G. R. Fulmer, A. J. M. Miller, N. H. Sherden, H. E. Gottlieb, A. Nudelman, B. M. Stoltz, J. E. Bercaw, K. I. Goldberg, *Organometallics* **2010**, *29*, 2176–2179.

[2] a) A. J. Arduengo, R. Krafczyk, R. Schmutzler, H. A. Craig, J. R. Goerlich, W. J. Marshall, M. Unverzagt, *Tetrahedron* **1999**, *55*, 14523–14534; b) L. Hintermann, *Beilstein J. Org. Chem.* **2007**, *3*, 22.

[3] a) J. W. Wielandt, D. Ruckerbauer, T. Zell, U. Radius, *Inorg. Synth.* **2010**, *35*, 120–125; b) A. J. Sicard, R. T. Baker, *Org Process Res Dev* **2020**, *24*, 2950–2952.

[4] H. Podall, W. E. Foster, A. P. Giraitis, *J. Org. Chem.* **1958**, *23*, 82–85.

[5] N. A. Giffin, M. Makramalla, A. D. Hendsbee, K. N. Robertson, C. Sherren, C. C. Pye, J. D. Masuda, J. A. C. Clyburne, *Organic & Biomolecular Chemistry* **2011**, *9*, 3672–3680.

[6] a) A. Merschel, D. Rottschäfer, B. Neumann, H.-G. Stammler, R. S. Ghadwal, *Organometallics* **2020**, *39*, 1719–1729; b) D. Rottschäfer, T. Glodde, B. Neumann, H.-G. Stammler, R. S. Ghadwal, *Chem. Commun.* **2020**, *56*, 2027–2030.

[7] N. G. Connelly, W. E. Geiger, *Chem. Rev.* **1996**, *96*, 877–910.

[8] S. Stoll, A. Schweiger, *J. Magn. Reson.* **2006**, *178*, 42–55.

[9] cwEPR – MATLAB Central File Exchange: https://www.mathworks.com/matlabcentral/fileexchange/73292–cwepr.

[10] O. V. Dolomanov, L. J. Bourhis, R. J. Gildea, J. A. K. Howard, H. Puschmann, *J. Appl. Cryst.* **2009**, *42*, 339–341.

[11] G. Sheldrick, *Acta Cryst. A* **2015**, *71*, 3–8.

[12] G. Sheldrick, *Acta Cryst. C* **2015**, *71*, 3–8.

[13] L. J. Bourhis, O. V. Dolomanov, R. J. Gildea, J. A. K. Howard, H. Puschmann, *Acta Cryst. A* **2015**, *71*, 59–75.

[14] F. Kleemiss, O. V. Dolomanov, M. Bodensteiner, N. Peyerimhoff, L. Midgley, L. J. Bourhis, A. Genoni, L. A. Malaspina, D. Jayatilaka, J. L. Spencer, F. White, B. Grundkötter-Stock, S. Steinhauer, D. Lentz, H. Puschmann, S. Grabowsky, *Chem. Sci.* **2021**, *12*, 1675–1692.

[15] F. Neese, *WIREs Comp. Mol. Sci.* **2022**, *12*, e1606.

[16] S. Grimme, J. G. Brandenburg, C. Bannwarth, A. Hansen, *J. Chem. Phys.* **2015**, *143*, 054107.

[17] F. Neese, F. Wennmohs, A. Hansen, U. Becker, *Chem. Phys.* **2009**, *356*, 98–109.

[18] a) C. Adamo, V. Barone, *J. Chem. Phys.* **1999**, *110*, 6158–6170; b) F. Weigend, R. Ahlrichs, *Phys. Chem. Chem. Phys.* **2005**, *7*, 3297–3305.

[19] C. A. Bauer, A. Hansen, S. Grimme, *Chem. Eur. J.* **2017**, *23*, 6150–6164.

[20] C. R. Landis, F. Weinhold, *Valency and Bonding: A Natural Bond Orbital Donor-Acceptor Perspective*, Cambridge University Press, Cambridge, **2005**.

[21] E. D. Glendening, J. K. Badenhoop, A. E. Reed, J. E. Carpenter, J. A. Bohmann, C. M. Morales, P. Karafiloglou, C. R. Landis, F. Weinhold, *NBO 7.0*, Theoretical Chemistry Institute, University of Wisconsin, Madison, **2018**.
